# Evolution of Class I cytokine receptors

**DOI:** 10.1186/1471-2148-7-120

**Published:** 2007-07-18

**Authors:** Clifford Liongue, Alister C Ward

**Affiliations:** 1 School of Medicine, Deakin University, Geelong, Victoria, Australia

## Abstract

**Background:**

The Class I cytokine receptors have a wide range of actions, including a major role in the development and function of immune and blood cells. However, the evolution of the genes encoding them remains poorly understood. To address this we have used bioinformatics to analyze the Class I receptor repertoire in sea squirt (*Ciona intestinalis*) and zebrafish (*Danio rerio*).

**Results:**

Only two Class I receptors were identified in sea squirt, one with homology to the archetypal GP130 receptor, and the other with high conservation with the divergent orphan receptor CLF-3. In contrast, 36 Class I cytokine receptors were present in zebrafish, including representative members for each of the five structural groups found in mammals. This allowed the identification of 27 core receptors belonging to the last common ancestor of teleosts and mammals.

**Conclusion:**

This study suggests that the majority of diversification of this receptor family occurred after the divergence of urochordates and vertebrates approximately 794 million years ago (MYA), but before the divergence of ray-finned from lobe-finned fishes around 476 MYA. Since then, only relatively limited lineage-specific diversification within the different Class I receptor structural groups has occurred.

## Background

Cytokines are a class of proteins that includes interleukins (ILs), interferons (IFNs), colony-stimulating factors (CSFs), and tumor necrosis factors (TNFs). These polypeptides are produced and secreted by cells in response to many stimuli and mediate their effects by binding to specific receptors on the surface of target cells [1,2]. Class I helical cytokines represent the largest group of cytokines and utilize a family of cell-surface receptors that are structurally divergent from those employed by other cytokines, such as the TNF receptor family and receptor tyrosine kinases [3]. The receptors for Class I helical cytokines consist of various receptor chains that associate in higher order homo- and heterotypic complexes. Signaling via these receptors has a myriad of roles, including a major influence on immunity and hematopoiesis [4-6]. There is considerable functional redundancy amongst Class I helical cytokine receptors. This is partially due to some cytokines binding to multiple receptor complexes, multiple cytokines binding to the same receptor complex, and the sharing of common signal transducing receptor chains – and so downstream signaling pathways – by different receptor complexes [7].

Class I helical cytokine receptors share little primary sequence homology [8]. Although individual Class I helical cytokine receptor chains vary in overall topology, they all posses a conserved 200 amino acid extracellular region that is required for ligand-receptor interactions [9]. This is known variously as the cytokine receptor homology domain (CHD), or the D200 [8,10]. The CHD consists of two tandem fibronectin type III (FBN) folds and contain distinctive elements that distinguish Class I from the Class II family [8]. The Class I receptor CHD contains two pairs of conserved cysteines linked via disulfide bonds and arranged in a CX-_(9–10)_-CXWX-_(26–32)_-CX-_(10–15)_-C motif within the first FBN fold. The second FBN fold contains a highly conserved, although slightly variable such as in GHR, WSXWS motif at its C-terminus [8,11]. In addition to the CHD, Class I cytokine receptor chains consist of a range of other modules, including extracellular immunoglobulin (Ig)-like and FBN domains, a transmembrane domain, and conserved intracellular motifs, including Box 1 and Box 2 motifs that are associated with Janus Kinase (Jak) docking [1,2,8].

**Figure 1:**
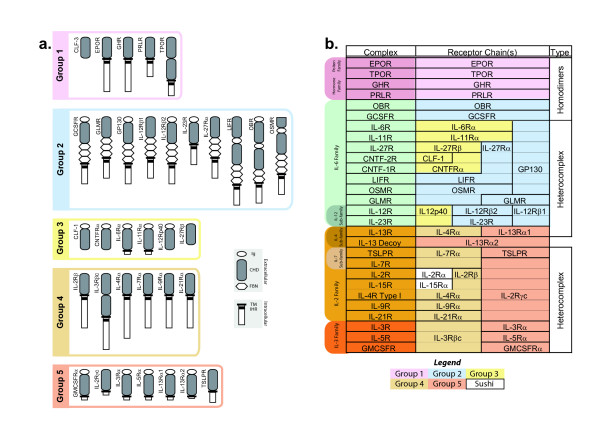
**Human Class I receptor chains and complexes**. **a**) Topological representation of the five structural groups of Class I receptor chains adapted from Boulay *et al*, 2003 [10]. Depicted are the Immunoglobulin-like (Ig) domains, Cytokine receptor Homology Domains (CHDs), including conserved cysteines (thin bands) and WSXWS motifs (thick band), Fibronectin type III (FBN) domains, Transmembrane (TM) regions, and Intracellular Homology Region (IHR) sequences. Full receptor names can be found in the list of abbreviations. **b**) Assembly of Class I receptor chains into functional receptor complexes. Individual receptor chains form either homodimers or various heterocomplexes that bind to specific ligands. For receptor complexes that bind to multiple cytokines, only one receptor complex is listed. Although constituents of receptor complexes of the IL-2R functional family, the IL-2Rα and IL-15Rα receptor chains are not members of the Class I family of receptors, but instead contain distinctive 'sushi domain' structures [55]. The orphan receptor chain CLF-3 was not included as its arrangement into a receptor complex is yet to be established.

Class I cytokine receptor complexes have traditionally been divided into families based on the use of common signal transducing chains within a receptor complex [3]. However, more recently researchers have placed the individual receptor chains into five groups. This is based on sequence and structural homology of the receptor and its cytokine ligand (Figure 1), which is potentially more reflective of evolutionary relationships [10]. Group 1 receptor chains have an extracellular domain that consists solely of a CHD. This group contains the erythropoietin receptor (EPOR), thrombopoietin receptor (TPOR), prolactin receptor (PRLR), and growth hormone receptor (GHR) chains that each form homodimers in the presence of their respective ligands [10,12], as well as an orphan receptor, CLF-3, of unknown function [10]. Group 2 receptors are the most numerous and are structurally related to the archetypal glycoprotein 130 (GP130). Typically, Group 2 receptors chains have an N-terminal Ig-domain and FBN modules between their CHD and transmembrane domains [10,13]. Group 3 receptor chains also generally possess an N-terminal Ig domain in addition to the CHD, and are either soluble or have short intracellular regions [10]. Receptor chains from Groups 2 and 3 collectively constitute the large IL-6R family of receptor complexes that often share GP130 as a common signal transducer [13]. Group 4 receptors typically consist solely of an extracellular CHD domain and long intracellular domains, whereas Group 5 receptors often possess extracellular Ig domains in addition to the CHD, and have short intracellular regions [10]. Group 4 and 5 receptor chains associate to form receptor complexes of the IL-2R and IL-3R families, with IL-2Rγ_c _and IL-3Rβ_c _being the shared chains respectively [3,7,10,11].

The Class I cytokine receptors are collectively responsible for a great diversity of cytokine-mediated responses. To further our understanding of the evolution of this complex signaling system, we have explored the repertoire of Class I receptor chains in sea squirt and zebrafish. This has revealed a period of massive expansion of Class I receptors between the divergence of urochordates and vertebrates around 794 million years ago (MYA), and divergence of ray-finned and lobe-finned fishes some 476 MYA. In contrast, more moderate lineage-specific expansion of these receptors has occurred since that time.

## Results

### Identification of putative sea squirt Class I cytokine receptor chains

**Figure 2:**
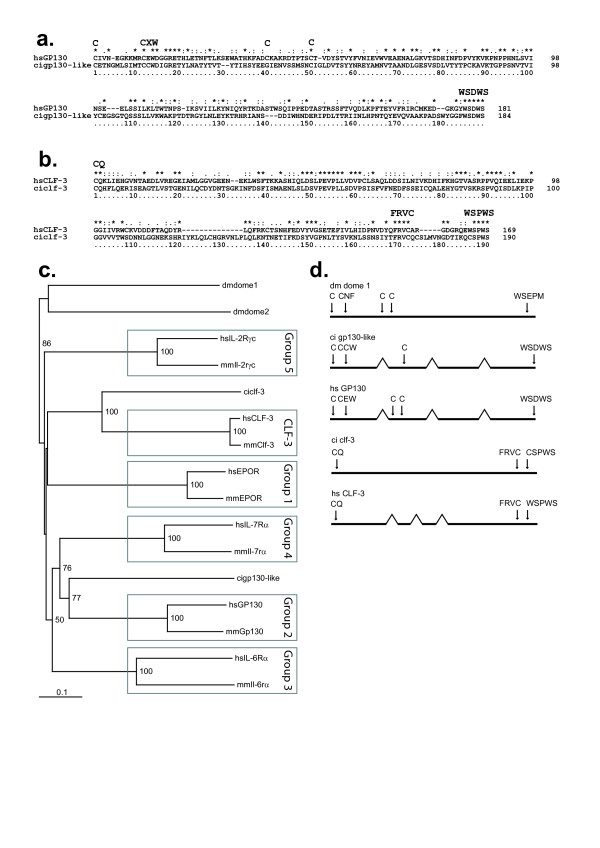
**Sea squirt Class I receptor chains**. **a-b**) Sequence alignments. Shown are alignments of the CHD of human (hs) and seasquirt (ci) GP130 (**a**) and CLF-3 (**b**) related sequences, with key residues annotated. **c**) Phylogenetic analysis of sea squirt receptors with representative sequences from each structural group of mammalian receptors and the two fruit fly dome receptors, using the Neighbourhood-Joining algorithm. Bootstrap values are indicated on branches as a percentage of 1000 replicates. **d**) Splice-site analysis. Schematic representation of the splice structure for the CHD of the above sequences in comparison to the fruit fly (dm) dome. Exons indicated with thick lines and introns with thin connecting lines. Specific residues indicated by standard one letter code.

Exhaustive analysis of sea squirt genomic databases revealed the presence of just two genes encoding putative Class I cytokine receptor chains. The presence of corresponding ESTs verified expression of both genes (Table 1). One of these showed broad sequence homology and conserved topology, including signature CHD motifs, with the archetypal GP130 of vertebrates and was designated cigp130-like (Figure 2a). The other receptor chain showed high conservation with sequences available for the orphan receptor, CLF-3, which essentially consists of just a CHD domain (Figure 2b). Phylogenetic analysis of the respective CHDs confirmed these designations, and revealed that each was equally divergent from the dome receptor sequences from *Drosophila melanogaster *(Figure 2c), despite the topological similarity between the dome sequences and cigp130-like. Furthermore, the ciclf-3 is encoded by a single exon, like the CHD of dmdome proteins (Figure 2d). In contrast, cigp130-like has a complex splice structure resembling that of GP130, that is largely conserved in other Class I receptors, with the exception of the vertebrate CLF-3 sequences that have a completely different splice pattern. No CLF-3-related sequences were found in *D. melanogaster *or *Anopheles gambiae*, suggesting that ciclf-3 was derived from at least a partial duplication of an ancestral dome-like sequence. There were no conserved syntenic relationships between either *D. melanogaster *or *C. intestinalis *cytokine receptors and their vertebrate counterparts (Additional file 1).

**Table 1:** Homology and expression analysis of Class I cytokine receptor genes from sea squirt and zebrafish^1^

				Related tetraodon sequence		Putative human homology				Expression		
										
		Receptor Sequence	Accession Number							RT-PCR		
					
				Receptor Sequence	Accession Number	Most similar	Percent Similarity (Identity)	Topology	Synteny	24 hpf	72 hpf	EST coverage of coding sequence (aa)
Sea Squrit		clf-3	BN000968	tnCRFA2	AAR25665	clf-3	30.5	clf-3	N/A	N/A	N/A	**1–463**
		gp130-like	BN000969	tnCRFA26	AAR25689	gp130	28.0	gp130	N/A	N/A	N/A	662–**1124**
Zebrafish	Group 1	clf-3	AM233512	tnCRFA2	AAR25665	CLF-3	66.4	CLF-3	CLF-3	+	+	**1–444**
		epor	BN000857	tnCRFA9	AAR25672	EPOR	25.3	EPOR	EPOR	+	+	353–464
		tpor	BN000861	tnCRFA22	AAR25685	TPOR	17.5	TPOR	-	-	+	67–244
		ghr.a	BN000777	tnCRFA5	AAR25668	GHR	29.0	GHR	GHR	+	+	**1–554**
		ghr.b	BN000776	tnCRFA6	AAR25669	GHR	33.1	GHR	-	+	+	**1**–436, 467–**570**
		prlr.a	AY375318	tnCRFA7	AAR25670	PRLR	24.8	PRLR	PRLR^	+	+	312–**602**
		prlr.b	BN000805	tnCRFA8	AAR25671	PRLR	33.3	PRLR	-	+	+	36–144
		crfa4	BN000914	tnCRFA4	AAR25667	GHR	(35.1)	GHR/PRLR	-	+	+	**1**–117, 205–364
	Group 2	obr	BN000731	tnCRFA30	AAR25693	OBR	22.4	OBR	OBR	+	+	245–444, 943–1100
		gcsfr	AM157796	-	-	GCSFR	25.6	GCSFR	GCSFR^^	+	+	-
		gp130	BN000730	tnCRFA26	AAR25689	GP130	31	GP130	GP130	+	+	-
		lifr.a	BN000768	tnCRFA29	AAR25692	OSMR	23	OSMR	LIFR	+	+	**1**–173, 246–425
		lifr.b	BN000769	tnCRFA29	AAR25692	OSMR	20.6	OSMR	LIFR	+	+	**1**–182, 452–**956**
		osmr	BN001082	tnCRFA28	AAR25691	LIFR	((32.2))	OSMR	OSMR	+	+	-
		il-12rβ2.a	BN000858	tnCRFA25	AAR25688	IL-12Rβ2	21.5	IL-12Rβ2	IL-12Rβ2	+	+	-
		il-12rβ2.b	BN000972	tnCRFA25	AAR25688	IL-12Rβ2	17.4	IL-12Rβ2	-	-	-	**1**–146, 445–549
		il-23r	BN000859	tnCRFA24	AAR25687	IL-23R	15.1	IL-12Rβ2	IL-23R	-	+	-
	Group 3	il-6rα	BN000832	tnCRFA21	AAR25684	IL-6Rα	17.3	IL-6Rα	IL-6Rα	+	+	463–**580**
		il-11rα	BN000772	tnCRFA17	AAR25680	IL-11Rα	26.9	IL-11Rα	-	+	+	**1**–248, 276–**402**
		il-27rβ	BN000734	tnCRFA3	AAR25666	IL-27Rβ	23.4	IL-27Rβ	-	+	+	**1–302**
		cntfr	BN000926	tnCRFA16	AAR25679	CNTFR	49.3	CNTFR	-	+	+	**1–357**
		clf-1.a	BN000719	tnCRFA1	AAR25664	CLF-1	61.7	CLF-1	-	+	+	**1**–287, 322–**389**
		clf-1.b	BN000970	tnCRFA1	AAR25664	CLF-1	54.7	CLF-1	CLF-1	+	+	**1**–270
		il-12p40.a	BN000854	tnCRFA14	AAR25677	IL-12p40	21.4	IL-12p40	IL-12p40	-	+	**1**–231
		il-12p40.b	BN000860	tnCRFA15	AAR25678	IL-12p40	20.7	IL-12p40	IL-12p40	-	+	72–**281**
	Group 4	il-2rβ	BN000818	tnCRFA12	AAR25675	IL-2Rβ	21.7	Group 4	IL-2Rβ(?)	-	+	**1**–138, 390–501
		il-4rα	BN000884	-	-	IL-4Rα	13.3	IL-4Rα	IL-4Rα	+	+	**1**–314, 356–**624**
		il-7rα	BN000775	tnCRFA11	AAR25674	IL-7Rα	20.5	IL-7Rα	IL-7Rα	-	+	**1–372**
		il-21rα.a	BN000773	tnCRFA13	AAR25676	IL-21Rα	23.8	IL-21Rα	-	+	+	**1–508**
		il-21rα.b	BN000971	tnCRFA20	AAR25683	IL-21Rα	(19.5)	-	-	-	-	-
		il-3rβc	BN000973	tnCRFA23	AAR25686	IL-3Rβc	14.1	IL-3Rβc	IL-3Rβc	-	-	**1**–193
	Group 5	il-2rγ c.a	BN000831	tnCRFA10	AAR25673	IL-2Rγ c	21.6	IL-2Rγ c	-	-	+	**1**–211, 314–**362**
		il-2rγ c.b	BN000817	tnCRFA10	AAR25673	IL-2Rγ c	18.0	IL-2Rγ c	IL-2Rγ c	+	+	-
		tslpr	-	-	-	TSLPR iso2	17.6	-	TSLPR	+	+	-
		il-13rα1	BN000774	tnCRFA19	AAR25682	IL-13Rα1	16.7	Group 5	IL-13Rα1	+	+	**1–411**
		il-13rα2	BN000679	tnCRFA18	AAR25681	IL-13Rα2	30.2	IL-13Rα2	IL-13Rα2	+	+	**1–405**

^1^Class I receptor chains are listed (and ordered by structural groups in the case of zebrafish) along with their Accession Number of the nucleotides (zebrafish) or protein (*T. nigroviridis*), closest human homologue based on percentage identity of the full-length protein, topology (Figure 1), synteny (Additional file 3) and expression status. Expression of zebrafish receptor chains was analyzed by RT-PCR on RNA extracted from either 24 hpf or 72 hpf, with the presence of an appropriately sized product following agarose gel electrophoresis indicated with a plus (+), or its absence with a minus (-). The existence of corresponding EST(s) is indicated for all sequences, including those from sea squirt. The numbers indicates amino acid coverage; bolded numbers represent either coverage of the start or stop codons, and absence with a minus (-). Brackets indicate sequences for which full-length sequence was unavailable, and so the CHD only was used, with double brackets indicating availability of only a partial CHD sequence. ^ indicates synteny with *T. nigroviridis *and ^^ indicates synteny with *T. rubripes*. Two additional receptors were omitted from the table and the final tally of receptors because these sequence was partial (tpor (GenBank accession no. BN001083)), have silent and missense mutations (il-4rα (GenBank accession no. BN000885)).

### Identification of zebrafish genes encoding class I cytokine receptor chains

**Figure 3:**
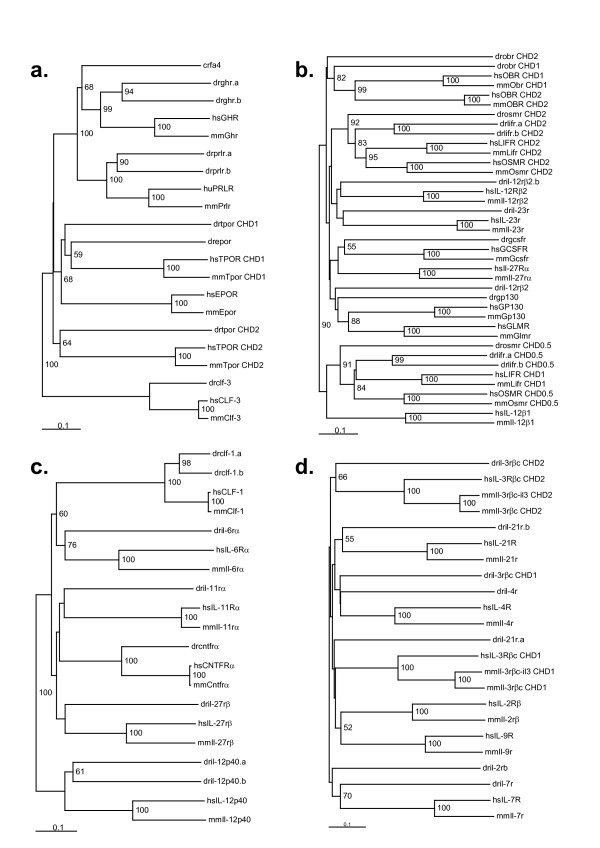
**Phylogenetic analysis of zebrafish Class I receptor chains**. Phylogenetic trees were created for each of the five structural groups of Class I receptor chains with sequences from zebrafish (dr) along with those from human (hs) and mouse (mm): Group 1 (**a**), Group 2 (**b**), Group 3 (**c**), Group 4 (**d**). Trees were calculated on the basis of multiple alignments of the CHD domains (Additional files 4, 5, 6, 7).

A total of 36 genes encoding putative Class I cytokine receptors chains were identified in zebrafish, including at least one representative from each structural group (Table 1, Figures 3 and 4). To both confirm that these genes were transcribed, as well as to commence their functional characterization, RT-PCR was performed on total RNA extracted from whole zebrafish embryos 24 hpf and 72 hpf using primers specific for each. This yielded appropriately sized products at one or both time points for all but three contigs. The presence of an EST corresponding to the putative gene provided alternate confirmation of expression for two of these, dril-3rβ_c _and dril-12rβ_2_.b. The presence of equivalent open reading frames in *Takifugu rubripes *or *T. nigroviridis *for the majority of zebrafish receptors provided additional support for this assertion (data not shown). Combined with the presence of long open reading frames in the contigs, this represented compelling evidence that the vast majority of contigs identified were coding genes and not pseudogenes. Only dril-21rα.b lacked any supporting evidence of expression, although a *T. nigroviridis *orthologue was present (data not shown).

Each of the Group 1 receptor chains found in mammals was represented at least once in zebrafish as determined by sequence, topology, and synteny conservation (Table 1), and confirmed by phylogenetic analysis (Figure 3a, Additional file 4). There were single zebrafish orthologues for CLF-3 EPOR, and TPOR. In contrast, the GHR and PRLR subfamily was expanded in zebrafish, with two clear homologues for PRLR (prlr.a, prlr.b), a single GHR, and an additional somatolactin receptor (slr) (Additional file 2). Furthermore an additional receptor was identified as belonging to the GHR and PRLR subfamily. Phylogenic analysis of the CHD domain grouped this receptor with GHR, it also has the presence of a typical WSXWS motif seen in the CHD of PRLR, but not GHR and SLR. Due to the lack of comparable tetrapod sequences it was named after the *T. nigroviridis *receptor, CRFA4, and is possibly a teleost specific receptor.

**Figure 5:**
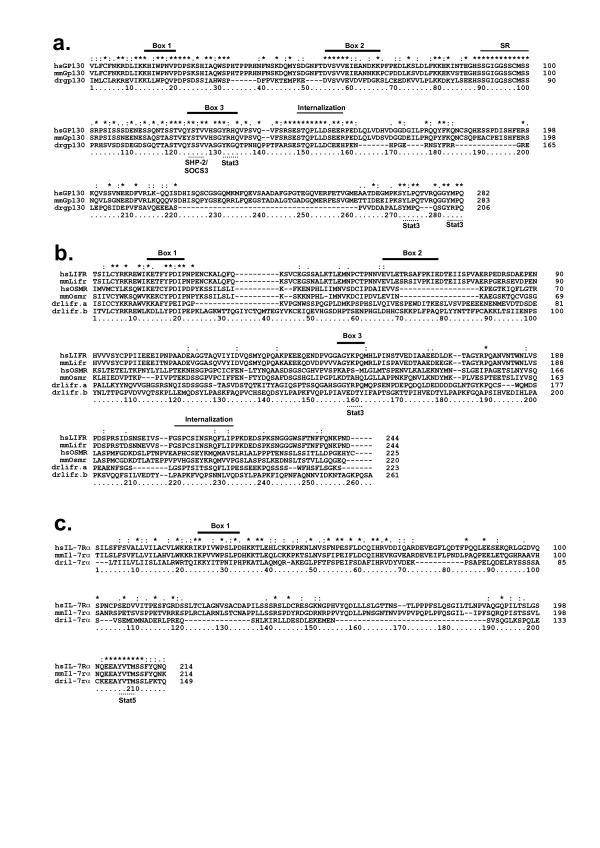
**Conservation of crucial intracellular motifs in zebrafish Class I receptor chains**. Multiple sequence alignments of the intracellular regions of homologues of GP130 (**a**), LIFR and OSMR (**b**), and IL-7Rα (**c**) from zebrafish, human and mouse. The solid lines above the alignments indicate key regions of conservation: Box 1, Box 2, Box 3, serine rich (SR), and the internalization motifs. The dashed line under the consensus sequence represents conserved docking sites for the signaling molecules SHP-2/Socs3, Stat3, and Stat5.

Of the ten mammalian Group 2 receptors, potential orthologues for eight were found in zebrafish (Table 1). This included one for the archetypal member of the group, GP130, which is the common shared component of many IL-6R family receptors. Interestingly, the zebrafish gp130 sequences did not form a distinct clade with the tetrapod GP130 sequences using CHD only strategies (Figure 3b, Additional file 5). However, multiple sequence alignment of the full-length protein revealed areas of significant homology throughout the protein that continued into functionally important areas of the intracellular domain, including Box 1 and Box 2 (Jak docking), a serine rich region, Box 3, a STQPLLDXEEX internalization motif, and tyrosine residues essential for docking of Stat3 (YXXQ) and SHP-2/SOCS3 (YXXV) [14-16] (Figure 5a). A zebrafish orthologue was also found for the closely-related GCSFR, which has the same overall topology to GP130 [10]. Synteny analysis suggested that there was no conserved relationship between humans and zebrafish (Additional file 3). However, there was conserved synteny between human and *T. rubripes*, thus providing further evidence that the zebrafish sequence is indeed a GCSFR orthologue. Mammalian IL-12Rβ_2 _also exhibits the same topology as GP130 and is syntenic to IL-23R with which it also shows high homology [17], although the latter lacks the FBN domains [10]. The zebrafish orthologues also maintain conserved synteny (Additional file 3), although zebrafish il-23r possesses FBN domains as does IL-12Rβ_2_, but lacks a transmembrane domain.

Several Group 2 receptors have a duplication of the CHD, partially in OSMR, fully in the closely related LIFR, and including a duplicated N-terminal Ig domain in the more divergent OBR [13]. Zebrafish have three related receptors that show similar sequence identity to both OSMR and LIFR. All sequences showed topological similarity to OSMR, having only a partial duplication of the N-terminal CHD, termed "CHD 0.5". Two of these sequences, lifr.a and lifr.b, are grouped with LIFR on the basis of phylogenic analysis of the CHD. One, lifr.a, showed extensive conservation of intracellular homology motifs with LIFR, including Box 1, Box 2, Box 3, multiple Stat3 binding sites and a C-terminal GSPXIXSXQFLIP internalization motif, absent in drlifr.b and OSMR [18] (Figure 5b). Although the intracellular region of drosmr was unable to be determined, the extracellular region showed strong sequence and synteny conservation with OSMR (Additional file 3). In contrast, a clear OBR homologue was identified in zebrafish, drobr, which maintains the same topology as mammalian OBR. No zebrafish homologues were found for the remaining Group 2 receptors – IL-12Rβ_1_, IL-27Rα, and GLMR – with their unique topology apparently specific to tetrapods.

Zebrafish homologues were found for all six mammalian members of Group 3 (Table 1). Single fish orthologues were identified for IL-27Rβ, IL-6Rα, CNTFRα, and IL-11Rα, while zebrafish possessed two homologues for both CLF-1 (drclf-1.a and drclf-1.b) and IL-12p40 (dril-12p40.a and dril-12p40.b). In each case, there was conserved topology and clades formed with high bootstrap values (Figure 3c, Additional file 6).

**Figure 6:**
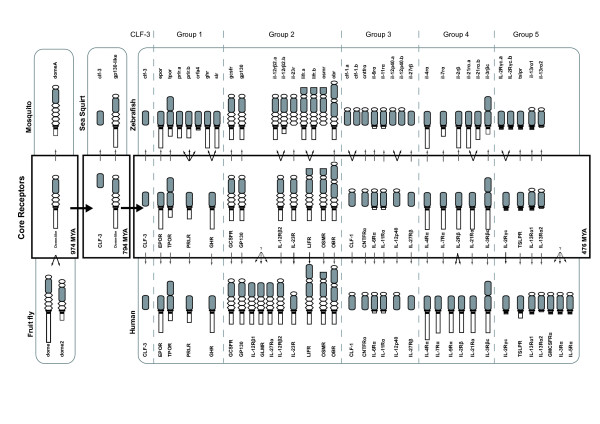
**Evolution of Class I cytokine receptors**. Class I cytokine receptors are depicted as in Figure 1. The rounded rectangles display all Class I cytokine receptors identified from fruit fly, mosquito, sea squirt, zebrafish, and humans. The bolded rectangles represent the hypothetical receptors present at the time of divergence of protostomes from deuterostomes, urochordates from vertebrates, and ray-finned from lobe-finned fishes, respectively. The vertebrate Class I receptor chains have been further divided into structural groups as described, with the exception of CLF-3 that has been considered separately on the basis of its distinct evolutionary history. Estimation of the times of the key evolutionary events, expressed in millions of years ago (MYA), are based on molecular genomic approaches [56]. Arrows represent presumed evolutionary relationships.

Mammalian Group 4 consists of six receptor chains in humans and seven in mice, the latter with an additional copy of IL-3Rβ_c _[11]. No zebrafish orthologues were found for either the additional IL-3Rβ_c _or for IL-9Rα, but each of the other five members was represented (Figure 3d, Additional file 7), albeit with weak homology. The identity of these marginal orthologues was confirmed by conserved topology for IL-3Rβ_c _(Figure 6), conserved synteny for IL-2Rβ, IL-4Rα, and IL-7Rα (Additional file 3). Further evidence for the IL-7Rα orthologues was aided by the conserved intracellular motifs, including Box 1 and a Stat5 docking site (Figure 5c) [19]. In contrast, there were two paralogues of IL-21Rα found, although neither displayed conserved synteny with human IL-21Rα.

**Figure 4:**
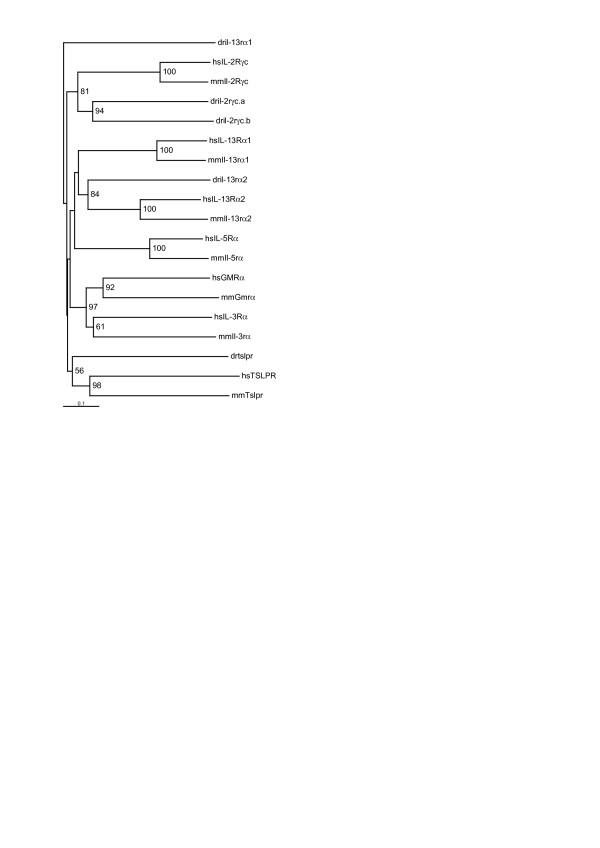
**Phylogenetic analysis of zebrafish Class I receptor chains**. A phylogenetic tree was created for structural Group 5 of Class I receptor chains with sequences from zebrafish (dr) along with those from human (hs) and mouse (mm). Trees were calculated on the basis of multiple alignments of the CHD domains (Additional file 8).

Finally only four of the seven mammalian Group 5 receptor chains were represented in zebrafish (Figure 4, Additional file 8). A single orthologue was identified for IL-13Rα2 and two zebrafish sequences were found that were most closely related to IL-2Rγ_c_, dril-2γ_c_.a, and dril-2γ_c_.b. Conserved synteny provided evidence for the identification of the zebrafish orthologues of TSLPR and IL-13Rα1, with the latter displaying only limited sequence homology. Additionally both the zebrafish tslpr identified and the second isoform of human TSLPR (Genbank accession no. NP_001012288) lack the first half of the CHD. No zebrafish orthologues were found for IL-3Rα, GMCSFRα, and IL-5R.

## Discussion

The aim of this study was to further our understanding of the evolution of class I cytokine signaling. Since Class I cytokines share little primary sequence [8], analysis of their receptors provides the best means to of achieving this aim. We therefore employed bioinformatic approaches to characterize the Class I cytokine receptor repertoire within the sea squirt and zebrafish genomes. However, the divergence of class I cytokine receptors, means that phylogenetic trees and alignments are sometimes unreliable. Therefore, the identification of receptor sequences was additionally guided by overall receptor topology and, in particular, conservation of synteny. This robust methodology successfully identified two representatives in sea squirt and 36 representatives in zebrafish. Comparison with the equivalent receptors in insects and mammals has yielded considerable insight into the evolution of this important family of receptors.

### Molecular details

Sea squirt possess a classical Class I receptor with the signature Ig-CHD-FBN-TM-Box 1 topology found in *D. melanogaster *dome and vertebrate GP130 proteins (including that of zebrafish). The presence of a single Class I receptor related to dome/GP130 in *Anopheles gambiae *confirmed that an archetypal Class I receptor of similar topology existed at 974 MYA, when protostomes and deuterostomes diverged, and from which all other receptors were derived (Figure 6). This was supported by the splicing pattern of cigp130-like that was largely conserved in all vertebrate Class I receptors, except CLF-3. In higher vertebrates, GP130 functions as part of heteromeric receptor complexes. However, the absence of other compatible receptor chains suggests that cigp130-like signals in a homodimeric manner, presumably similar to the dome receptors of *D. melanogaster*.

The second sea squirt receptor showed unequivocal homology to CLF-3 and, like the CHD of dome, was the product of a single coding exon. This suggests that the CLF-3 precursor arose from a duplication of at least the CHD of an archetypal dome-like receptor, and that it subsequently developed an independent splicing pattern in the evolution of vertebrate CLF-3 genes. The high conservation of CLF-3 despite its ancient origins are indicative of an essential conserved function for this protein. However, despite the presence of a WSXWS motif, CLF-3 proteins lack the signature CHD CX-_(9–10)_-CXWX-_(26–32)_-CX-_(10–15)_-C motif, and possibly a leader sequence, indicating an altered function compared to other class I cytokine receptors. This suggests that CLF-3 may be best considered as a unique protein despite its shared evolutionary origins to the other class I cytokine receptors. This has some similarities to Tissue Factor, which has evolved a divergent biochemical function in blood coagulation despite sharing origins with the class II cytokine receptors [20].

Therefore, by the time of the last common ancestor of vertebrates and urochordates 794 MYA, two different receptor topologies (and the WSXWS motif) had been generated, only one of which would ultimately generate the great diversity of Class I cytokine receptor chains seen in higher vertebrates, although the exact details remain elusive (Figure 2c).

Comparison of the zebrafish and mammalian Class I cytokine receptor repertoires suggest that between 794 MYA and 476 MYA there was a considerable expansion of both the number of receptor topologies (combinations of structural subdomains) as well as the total number of receptor chains. The result was eight different topologies for the 27 core receptor chains that were likely to be present in the common ancestor of fish and mammals (Figure 6). During this period it has been hypothesized that two whole genome duplications have occurred [21]. These events would explain some of the increase in the diversity of receptor chains. However, only one CLF-3-like receptor is present in both humans and zebrafish. Thus the two whole genome duplications alone theoretically account for only four GP130-related receptors. Therefore, other processes, such as tandem and *en bloc *duplication have likely driven this expansion.

Since their last common ancestor, the number of cytokine receptor chains have increased to 36 receptors in zebrafish and 36 in humans, although the cause of this expansion is likely different in each lineage. The teleost lineage is believed to have experienced a further whole genome duplication event [22], which is probably responsible for several novel teleosts receptors, such as the paralogues of the ghr, prlr, lifr, il-12rβ_2_, clf-1, il-12p40, and il-2rγ_c_. However, evidence of *en bloc *duplications is also apparent in the il-21rα paralogues, as well as crfa4. In contrast, receptor repertoire expansion within mammals appears primarily to be the result of tandem or *en bloc *duplications. This is typified in the generation of GLMR, IL-12Rβ1 and IL-27Rα within Group 2, and of the Group 5 receptors that lie relatively adjacent on chromosome X [10]. Similar duplication apparently explain the ongoing cytokine receptor (and ligand) evolution in *D. melanogaster *[10].

### Functional considerations

Some Class I receptor chains have maintained a one:one (1:1) homologue relationship between mammals and teleosts, suggesting conserved functions. For example, a single GP130 orthologue was also present in teleosts with conserved intracellular Jak and Stat3 docking motifs [14-16]. Moreover, many of the Group 3 receptor chains that form complexes with GP130 also largely showed a 1:1 homologue relationship. Other orthologous 1:1 receptor genes found were EPOR, CLF-3, GCSFR, IL-23Rα, OBR, CNTFRα, IL-6Rα, IL-11Rα, IL-27Rβ, IL-7Rα, IL-3Rβ_c_, IL-13Rα1, IL-13Rα2, and TSLPR. Many of these also showed the highest percent similarity and conserved topology, further attesting to their likely conserved roles. An obvious exception was IL-23Rα, which showed different topology in teleosts and tetrapods, lacking a transmembrane domain in the former and FBN repeats in the latter. However, conserved synteny and sequence within the CHD strongly suggest that the zebrafish sequence is an il-23rα orthologue.

In contrast, lineage-specific expansion was seen in both teleosts and mammals probably representing a diversification of function. In support of this, expansion of Group 1 receptor chains within teleosts that has produced multiple paralogues for the PRLR/GHR subgroup. Prolactin signaling in fish plays a drastically different function with diverse roles such as pigment cell function and osmoregulation [23,24], contrasting to its role in mammary gland development and lactation in mammals [25]. It remains speculative as to whether prolactin binds to prlr.a or prlr.b, to both prlr.a and prlr.b, or whether there may be more than one prolactin. Fish also have a unique cytokine related to both growth hormone and prolactin, somatolactin, which has roles in background adaptation, stress response and acid-base regulation [26]. Indeed zebrafish has recently been shown to have two somatolactins, slα and slβ, a growth hormone, and a prolactin [27]. Zebrafish has a clear orthologue of salmonid slr, although whether slα or slβ, or both bind to zebrafish slr needs to be established. The zebrafish ghr is less problematic, clearly orthologues to the single ghr in salmonids (see additional file 2) [28,29]. The remaining more divergent homologue of PRLR/GHR, crfa4, also does not have a direct mammalian orthologue, and is most closely related sequence to the orphan *T. nigroviridis *receptor CRFA4. Other receptor genes expansions are limited to Group 3 (IL-12p40 and CLF-1) and Group 4 (IL-21Rα), although the duplication of CLF-1 appears to be specific for zebrafish rather than all teleosts, as they are absent in pufferfish (data not shown).

Mammalian lineage-specific expansion has occurred in other structural groups. Within Group 2 the IL-12Rβ_1_, IL-27Rα, and GLMR receptor chains are unique to tetrapods. These receptors are topologically similar lacking an N-terminal Ig-domain – a unique topology – and so are likely derived from the same ancestral receptor. IL-12Rβ_1 _a member of the IL-12R subfamily is a shared component of both IL-12R and IL-23R complexes promoting Th1 cell and memory T cell development respectively [30]. IL-27R is also involved in the regulation of Th1 cell differentiation [31]. It remains to be seen whether these higher order immune functions are conserved in fish [32]. In contrast, IL-9Rα likely arose from a duplication of IL-2Rβ. In mammals, IL-9Rα has been implicated in asthma and immune responses against parasites [33], the former property clearly not relevant in an aquatic environment. Lineage-specific expansion was most evident in Group 5 with no teleost homologues found for several mammalian receptors. Interestingly, this is largely limited to receptor chains that form complexes of the IL-3R functional family. Specifically, GMCSFRα, IL-3Rα, and IL-5Rα could have been formed via multiple rounds of duplication of either IL-13Rα1, IL-13Rα2, or TSLPR on chromosome X. The IL-3R functional family is involved in the development of eosinophils, granulocytes, macrophages, and monocytes. Of these, IL-5 signaling plays a specific role in eosinopoiesis [34]. Interestingly, teleosts have no clear analogue for this cell-type [35], and we have been unable to identify orthologues of a range of eosinophil-specific genes in zebrafish (data no shown). Thus, the IL-5R may have played a direct role in the evolutionary ontogeny of this cell type. The expansion of the IL-3R family has continued within mammalian lineages as IL-3Rβ_c _has been duplicated to generate, IL-3Rβ_c _and IL-3Rβ_IL-3 _in mouse [11], that latter of which is a pseudogene in humans.

## Conclusion

Innate immunity, including complement and Toll receptors are well developed in invertebrates, as characterized by *D. melanogaster *[36,37]. In this organism, the domeless receptors play diverse roles in oogenesis, eye and gut development, with a more minor role in the *D. melanogaster *immune system. Adaptive immunity, on the other hand, arose after sea squirt [38]. Our data suggest that a massive increase in the number of Class I cytokine receptors correlated with the development of acquired immunity and the refinement of innate immunity. This suggests that the expansion and subsequent specialization of dome/gp130-like receptors may have played a major role in the evolution of the immune system. In contrast, chemokines and their receptors, which are not present in sea squirt [39], probably only contributed at later stages of immune system evolution. Further data mining or functional studies of species between urochordates and teleosts, such as lampreys and hagfish, is required for additional insight into this interesting family of receptors, while it is anticipated that reverse genetics approaches in zebrafish may shed light on the function of CLF-3 receptors.

## Methods

### Data mining and sequence assembly

Searches were performed using Class I cytokine receptor sequences and a Class I CHD consensus motif [10]. These sequences were used to systematically interrogate the sea squirt genomic [40] and Expressed Sequence Tag (EST) databases [41] as well as the zebrafish EST, genomic, and whole genome shotgun (WGS) databases [42], using tBLASTn. All independent sequences possessing E values > 0.1 were extracted for further analysis. GenomeScan [43] was used to predict coding exons from sequences derived solely from WGS or genomic scaffolds, some of which were manually adjusted on the basis of known intron-exon boundaries in other organisms. Nucleotide sequences were assembled using Sequencher 4.1.4 (Gene Codes Corporation). Any apparently incomplete contigs were extended by iterative BLASTn searches using the relevant contig terminus until the entire putative coding sequence had been identified, with any remaining gaps closed by sequencing of appropriate reverse transcription-polymerase chain reaction (RT-PCR) product. The position of intron/exon boundaries was determined by alignment of cDNA and genomic sequences, applying the GT-AG splice rule where possible [44].

### Reverse Transcription-Polymerase Chain Reaction

Total RNA was extracted from zebrafish embryos at 24 and 72 hours post fertilization (hpf). This was converted into cDNA using oligo-dT primers (Roche) and reverse-transcriptase (Invitrogen), which was used as a template for PCR with Taq polymerase (Invitrogen) in an iCycler thermocycler (Biorad) and oligonucleotides designed to span at least one intron to eliminate the potential for amplifying genomic sequences. Negative control templates were water and samples in which the reverse transcriptase was omitted. If required for contig assembly, RT-PCR products were cloned into pGEM-T EASY (Promega) and subsequently sequenced using Big Dye Terminator (Applied Biosystems).

### Sequence analysis and nomenclature

The probable identity of each encoded receptor chain was determined by pBLAST searching with the respective conceptual translations. The initial BLAST search was followed by multiple sequence alignments using AlignX 9 (Invitrogen) and ClustalX 1.83 [45]. The latter were used to create bootstrapped phylogenetic tree of 1000 replicates with the Neighbor-Joining algorithm, formatted using njplot [46], and viewed in Treeview 1.6.6 [47]. Additional analysis using maximum parsimony [48] and maximum likelihood [49] algorithms was performed with phylo_win [50] and phylip [51] packages to confirm phylogenetic topologies.

Synteny analysis was performed on the putative receptor chains to further access the identity of these receptor chains. Ensembl [52] was used to perform synteny analysis using version 2 of the *C. intestinalis *(JGI 2) assembly and version 6 of the *D. rerio *(Zv6) assembly. Synteny data from either *C. intestinalis *or *D. rerio *was primarily compared to that of humans (NCBI 36). However, in certain cases *Tetraodon nigroviridis *(TETRAODON 7), *Takifugu rubripes *(FUGU 4.0), *Xenopus tropicalis *(JGI 4.1), *Gallus gallus *(WASHUC 1), and *Mus musculus *(NCBI m36) were also used in the synteny comparison.

The final assignment of identity was guided by conservation of topology, synteny, and overall sequence such as the conservation of functional domains, including intracellular sequences that mediate interactions with the key signal transduction pathway used by cytokine receptors, the Jak-Stat (Signal transducer and activator of transcription) pathway [53]. The nomenclature for the zebrafish genes followed the conventions of zebrafish information network (ZFIN) [54]. Sea squirt genes were named using similar criteria. All sequences were subsequently deposited in GenBank (Table 1) except zfprlr.a (GenBank accession no. AY375318).

## Abbreviations

BLAST Basic local alignment search tool

CHD Cytokine receptor homology domain

CLF Cytokine receptor like factor

CNTF Ciliary neurotrophic factor

CSF Colony-stimulating factor

CRFA Cytokine receptor family, Class I

EPOR Erythropoietin receptor

EST Expressed sequence tag

FBN Fibronectin

GCSF Granulocyte-CSF

GH Growth hormone

GLMR GP130-like monocyte receptor

GP130 Glycoprotein 130

HPF Hours post fertilization

IFN Interferon

IL Interleukin

JAK Janus Kinase

LIF Leukemia inhibitory factor

MYA Million years ago

OB obesity (leptin)

OSM Oncostatin M

PRL Prolactin

R Receptor

SL Somatolactin

STAT Signal transducers and activators of transcription

TM Transmembrane

TNF Tumor necrosis factor

TPO Thrombopoietin

TSLP Thymic stromal lymphopoietin

WGS Whole genome shotgun

## Authors' contributions

CL performed the bulk of the data mining, phylogenetic analysis, and expression studies, as well as drafting the manuscript. ACW conceived the project, participated in its design and analysis and helped to draft the manuscript. All authors read and approved the final manuscript.

## Supplementary Material

Additional file 1 **Synteny analysis of *C. intestinalis *and *D. melanogaster *cytokine receptor sequences**. Additional file is a pdf document that contains supplementary data about the synteny analysis performed in *C. intestinalis *and *D. melanogaster*. Grey boxes represents genes in humans, and grey outline boxes represent genes in zebrafish. Click here for file

Additional file 2 **Phylogenetic analysis of teleost ghr, prlr, and slr**. Additional file is a pdf document that contains supplementary data about the phylogenetic analysis performed for the gh, prl, and sl receptor family. The phylogenetic tree was calculated using full-length gh, prlr, and sl receptor sequences from zebrafish along with those from human (hs), mouse (mm), *T. nigroviridis *(tn), and *Oryzias latipes *(ol). Additionally mammalian CLF-3 and EPOR were used as outgroups. Click here for file

Additional file 3 **Synteny analysis of vertebrate Class I cytokine receptor sequences**. Additional file is a pdf document that contains supplementary data about the synteny analysis performed primarily comparing zebrafishand humans. Additionally *T. nigroviridis*, *T. rubripes*, *X. Tropicalis, G. gallus*, and *M. musculus *were also used in the synteny comparison where required. Grey boxes represents genes in humans, and grey outline boxes represent genes in zebrafish. The partial sequences (tpor), and a sequence with a missense mutation (il-4r) have also been included. Click here for file

Additional file 4 **Alignment of the CHD of group 1 receptors**. Additional file is a pdf document that contains supplementary data. Included is an alignment of group 1 Class I cytokine receptors that were used to calculate the phylogenetic tree in Figure 3a. Click here for file

Additional file 5 **Alignment of the CHD of group 2 receptors**. Additional file is a pdf document that contains supplementary data. Included is an alignment of group 2 Class I cytokine receptors that were used to calculate the phylogenetic tree in Figure 3b. Click here for file

Additional file 6 **Alignment of the CHD of group 3 receptors**. Additional file is a pdf document that contains supplementary data. Included is an alignment of group 3 Class I cytokine receptors that were used to calculate the phylogenetic tree in Figure 3c. Click here for file

Additional file 7 **Alignment of the CHD of group 4 receptors**. Additional file is a pdf document that contains supplementary data. Included is an alignment of group 4 Class I cytokine receptors that were used to calculate the phylogenetic tree in Figure 3d. Click here for file

Additional file 8 **Alignment of the CHD of group 5 receptors**. Additional file is a pdf document that contains supplementary data. Included is an alignment of group 5 Class I cytokine receptors that were used to calculate the phylogenetic tree in Figure 4. Click here for file
