# Supplementary figures and images for: Evolution of Class I cytokine receptors
Source: BMC Evol Biol. 2007 Jul 18;7:120. doi: 10.1186/1471-2148-7-120 (PMC1963337; doi:10.1186/1471-2148-7-120)

ciclf-3  
ch 3p

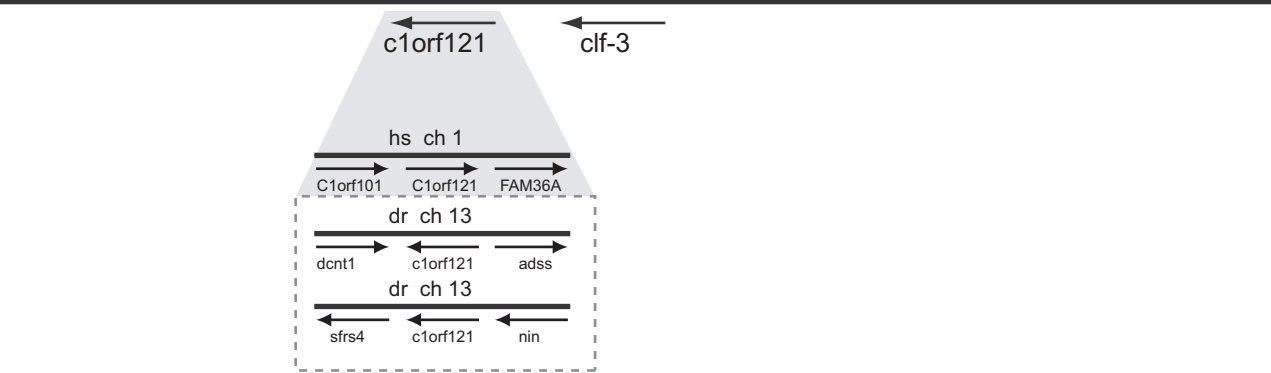

cigp130-like  
ch 5q

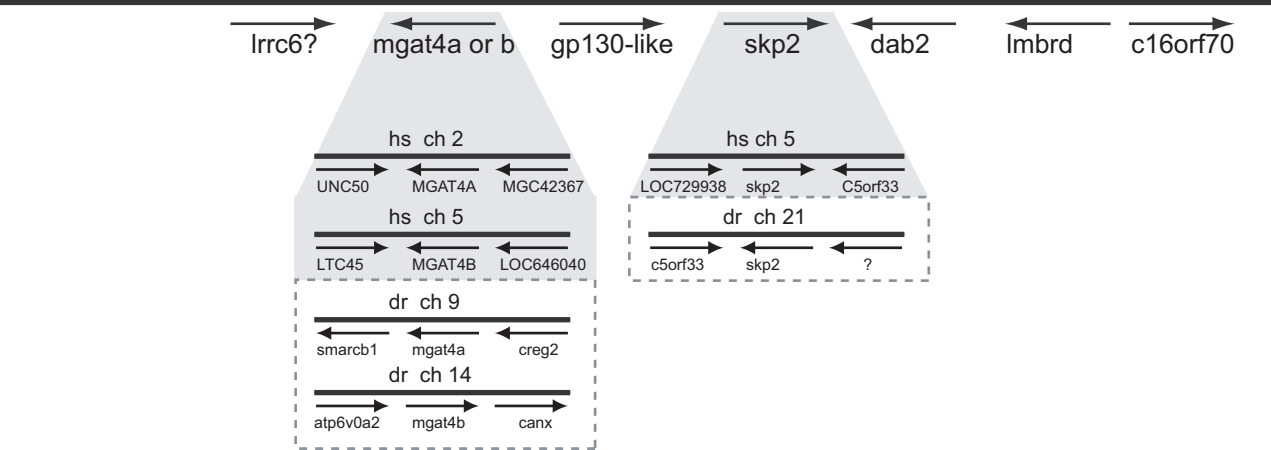

dmdome and dome\_A  
ch X

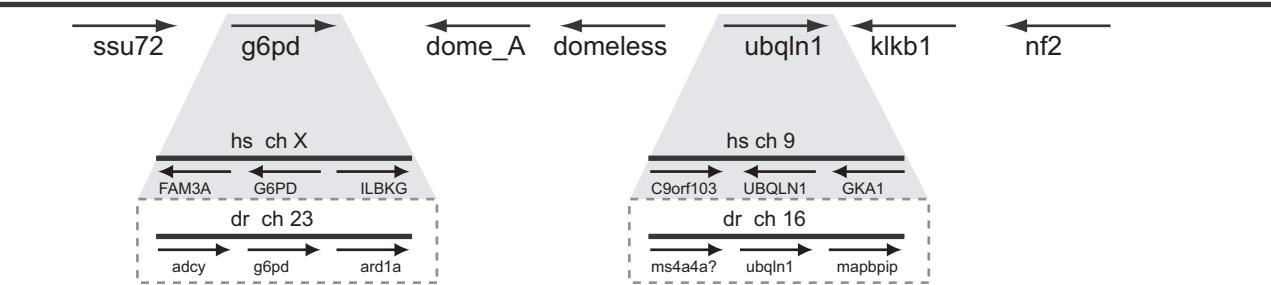

Supplement: Additional file 1 — Synteny analysis of C. intestinalis and D. melanogaster cytokine receptor sequences. Additional file is a pdf document that contains supplementary data about the synteny analysis performed in C. intestinalis and D. melanogaster. Grey boxes represents genes in humans, and grey outline boxes represent genes in zebrafish. [file 1471-2148-7-120-S1.pdf]

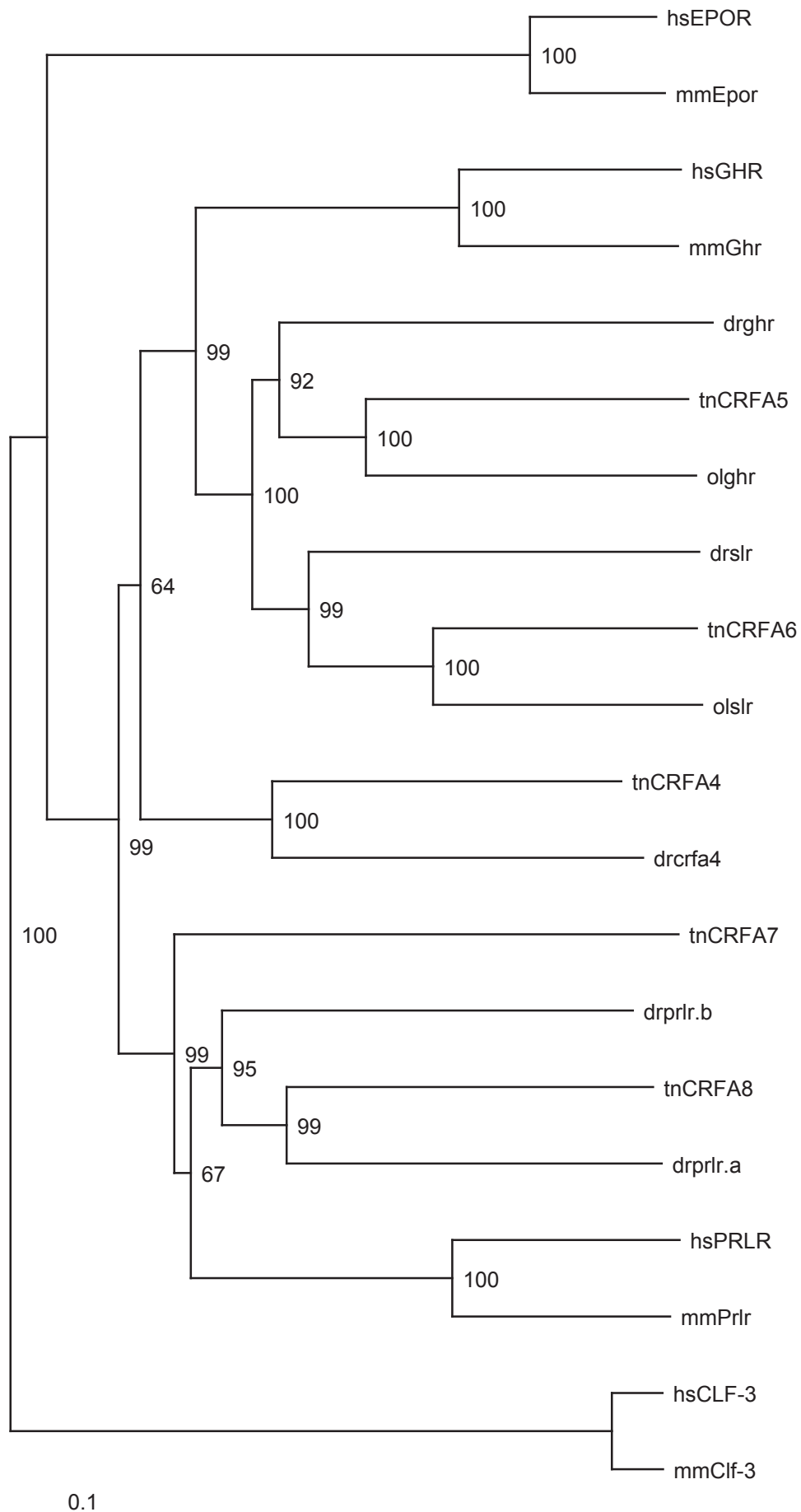

Supplement: Additional file 2 — Phylogenetic analysis of teleost ghr, prlr, and slr. Additional file is a pdf document that contains supplementary data about the phylogenetic analysis performed for the gh, prl, and sl receptor family. The phylogenetic tree was calculated using full-length gh, prlr, and sl receptor sequences from zebrafish along with those from human (hs), mouse (mm), T. nigroviridis (tn), and Oryzias latipes (ol). Additionally mammalian CLF-3 and EPOR were used as outgroups. [file 1471-2148-7-120-S2.pdf]
