# Supplementary material for: Evolution of Class I cytokine receptors
Source: BMC Evol Biol. 2007 Jul 18;7:120. doi: 10.1186/1471-2148-7-120 (PMC1963337; doi:10.1186/1471-2148-7-120)
Supplement: Additional file 3 — Synteny analysis of vertebrate Class I cytokine receptor sequences. Additional file is a pdf document that contains supplementary data about the synteny analysis performed primarily comparing zebrafishand humans. Additionally T. nigroviridis, T. rubripes, X. Tropicalis, G. gallus, and M. musculus were also used in the synteny comparison where required. Grey boxes represents genes in humans, and grey outline boxes represent genes in zebrafish. The partial sequences (tpor), and a sequence with a missense mutation (il-4r) have also been included. [file 1471-2148-7-120-S3.pdf]

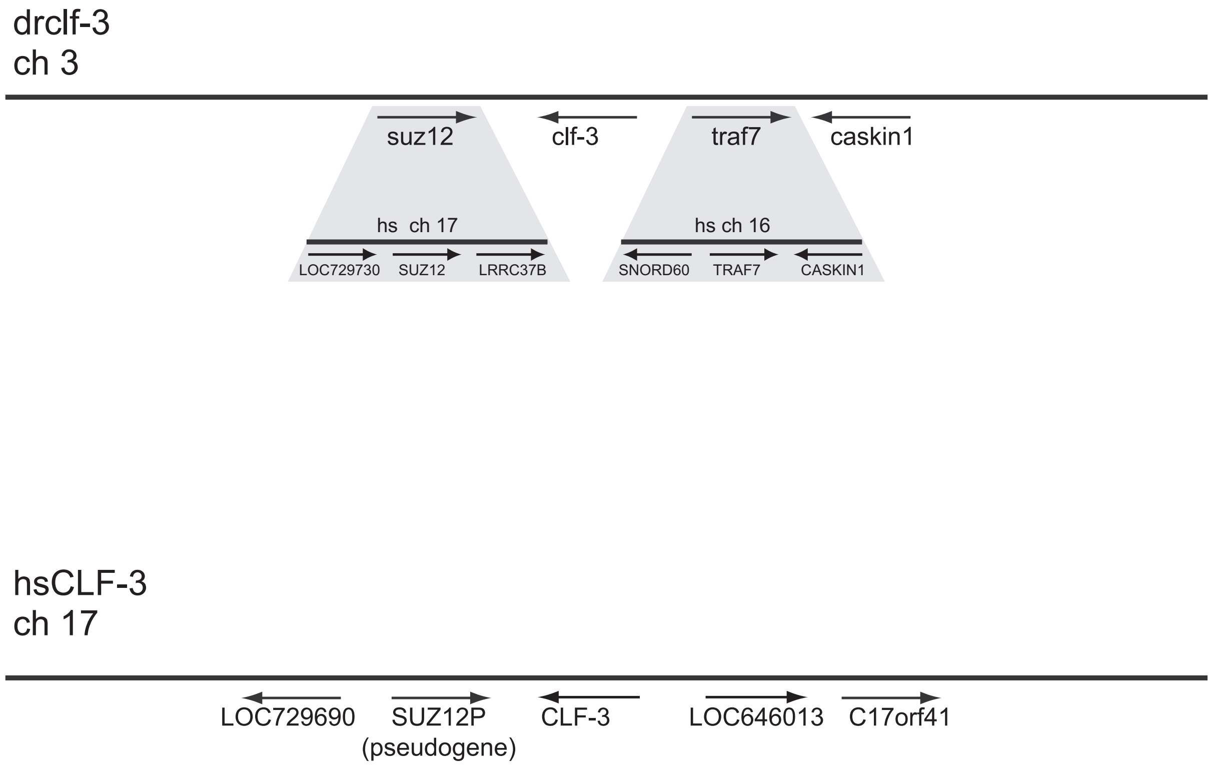
drclf-3
ch 3
suz12
clf-3
traf7
caskin1
hs ch 17
LOC729730
SUZ12
LRRC37B
hs ch 16
SNORD60
TRAF7
CASKIN1
hsCLF-3
ch 17
LOC729690
SUZ12P
(pseudogene)
CLF-3
LOC646013
C17orf41

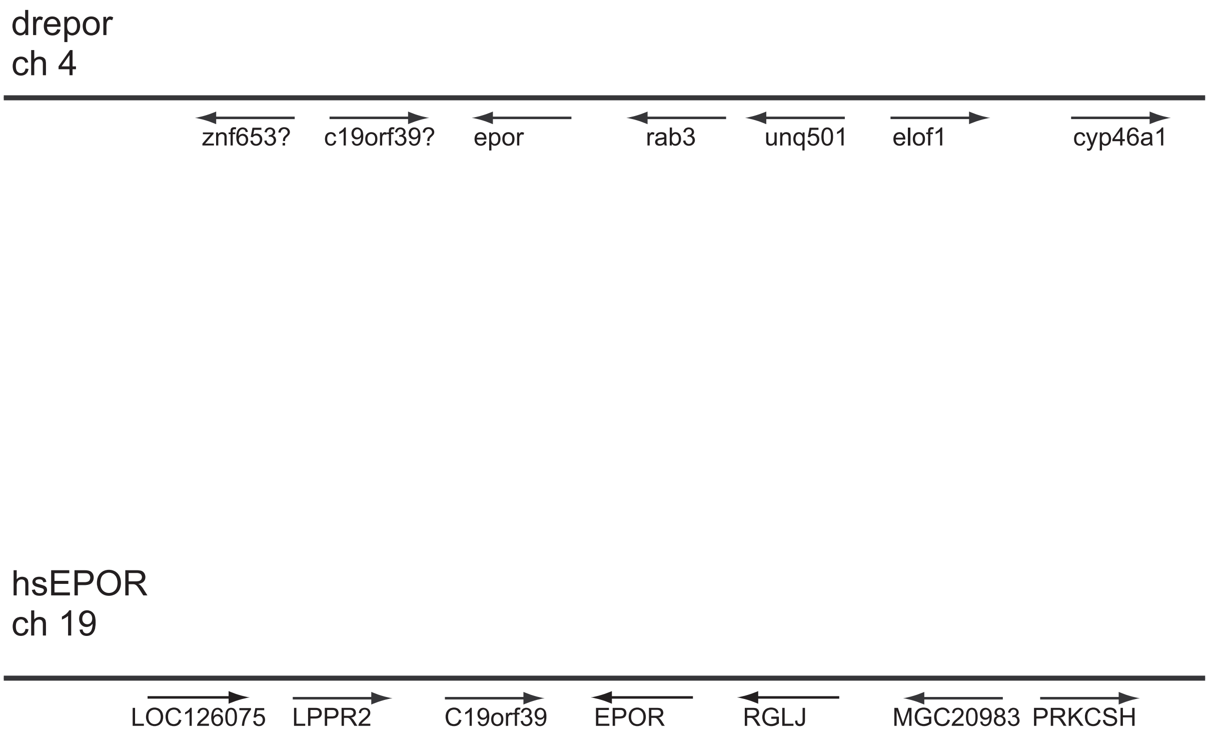
drepor
ch 4
znf653?
c19orf39?
epor
rab3
unq501
elof1
cyp46a1
hsEPOR
ch 19
LOC126075
LPPR2
C19orf39
EPOR
RGLJ
MGC20983
PRKCSH

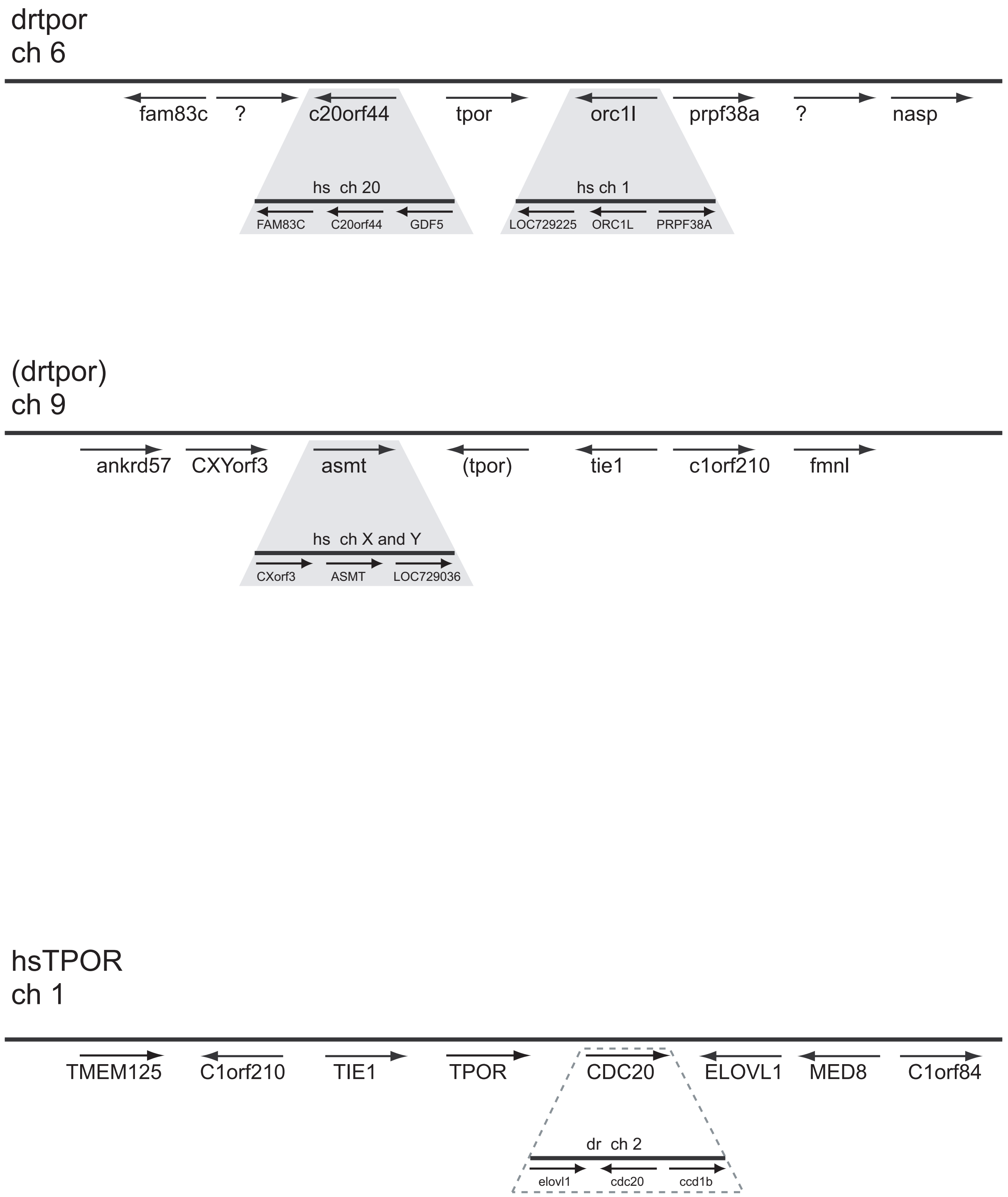
drtpor
ch 6
fam83c
?
c20orf44
tpor
orc1l
prpf38a
?
nasp
hs ch 20
FAM83C
C20orf44
GDF5
hs ch 1
LOC729225
ORC1L
PRPF38A
(drtpor)
ch 9
ankrd57
CXYorf3
asmt
(tpor)
tie1
c1orf210
fmnl
hs ch X and Y
CXorf3
ASMT
LOC729036
hsTPOR
ch 1
TMEM125
C1orf210
TIE1
TPOR
CDC20
ELOVL1
MED8
C1orf84
dr ch 2
elovl1
cdc20
ccd1b

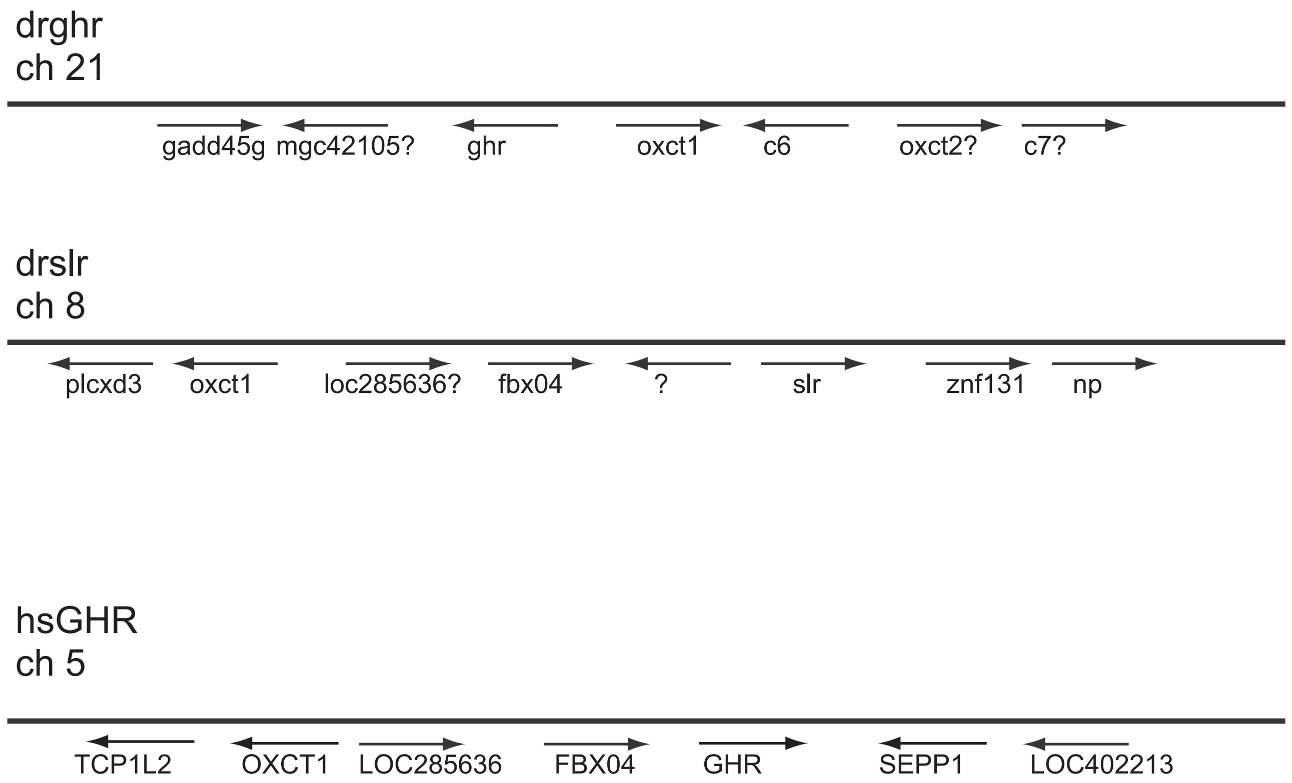
drghr
ch 21
gadd45g
mgc42105?
ghr
oxct1
c6
oxct2?
c7?
drslr
ch 8
plcxd3
oxct1
loc285636?
fbx04
?
slr
znf131
np
hsGHR
ch 5
TCP1L2
OXCT1
LOC285636
FBX04
GHR
SEPP1
LOC402213

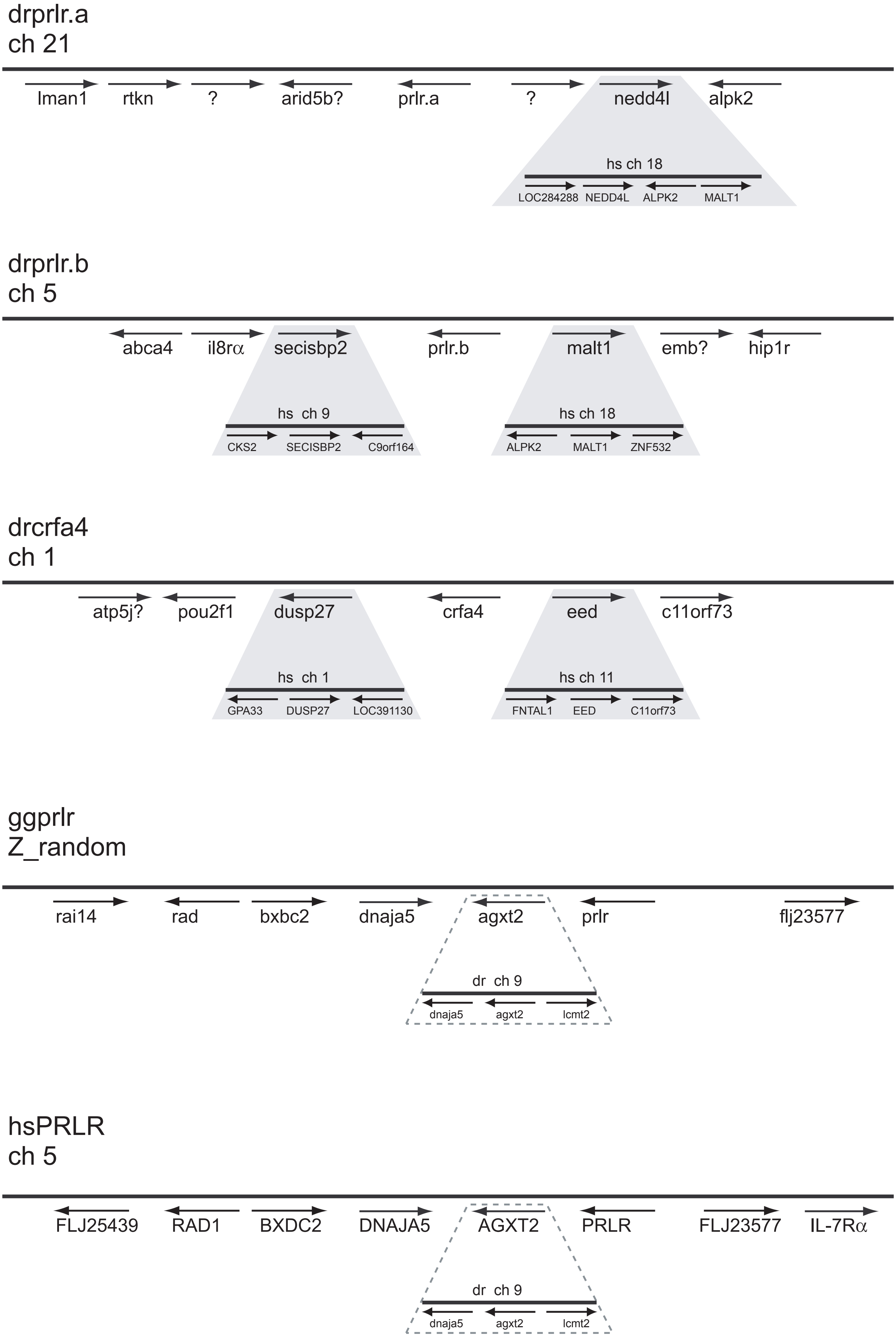
drprlr.a
ch 21
lman1
rtkn
?
arid5b?
prlr.a
?
nedd4l
alpk2
hs ch 18
LOC284288
NEDD4L
ALPK2
MALT1
drprlr.b
ch 5
abca4
il8rα
secisbp2
prlr.b
malt1
emb?
hip1r
hs ch 9
CKS2
SECISBP2
C9orf164
hs ch 18
ALPK2
MALT1
ZNF532
drcrfa4
ch 1
atp5j?
pou2f1
dusp27
crfa4
eed
c11orf73
hs ch 1
GPA33
DUSP27
LOC391130
hs ch 11
FNTAL1
EED
C11orf73
ggprlr
Z_random
rai14
rad
bxbc2
dnaja5
agxt2
prlr
flj23577
dr ch 9
dnaja5
agxt2
lcmt2
hsPRLR
ch 5
FLJ25439
RAD1
BXDC2
DNAJA5
AGXT2
PRLR
FLJ23577
IL-7Rα
dr ch 9
dnaja5
agxt2
lcmt2

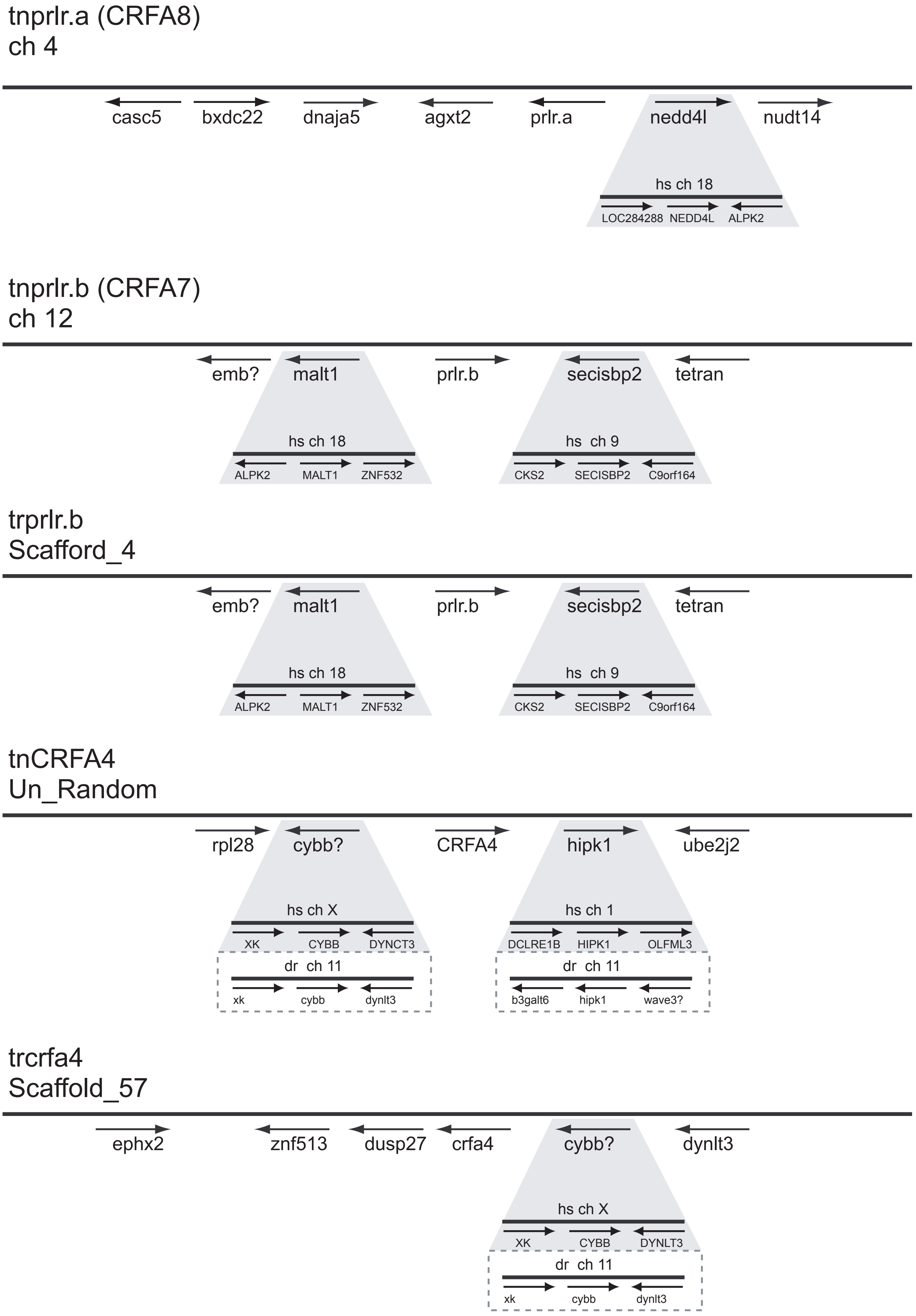
tnprlr.a (CRFA8)
ch 4
casc5
bxdc22
dnaja5
agxt2
prlr.a
nedd4l
nudt14
hs ch 18
LOC284288
NEDD4L
ALPK2
tnprlr.b (CRFA7)
ch 12
emb?
malt1
prlr.b
secisbp2
tetran
hs ch 18
ALPK2
MALT1
ZNF532
hs ch 9
CKS2
SECISBP2
C9orf164
trprlr.b
Scafford_4
emb?
malt1
prlr.b
secisbp2
tetran
hs ch 18
ALPK2
MALT1
ZNF532
hs ch 9
CKS2
SECISBP2
C9orf164
tnCRFA4
Un_Random
rpl28
cybb?
CRFA4
hipk1
ube2j2
hs ch X
XK
CYBB
DYNCT3
dr ch 11
xk
cybb
dynlt3
hs ch 1
DCLRE1B
HIPK1
OLFML3
dr ch 11
b3galt6
hipk1
wave3?
trcrfa4
Scaffold_57
ephx2
znf513
dusp27
crfa4
cybb?
dynlt3
hs ch X
XK
CYBB
DYNLT3
dr ch 11
xk
cybb
dynlt3

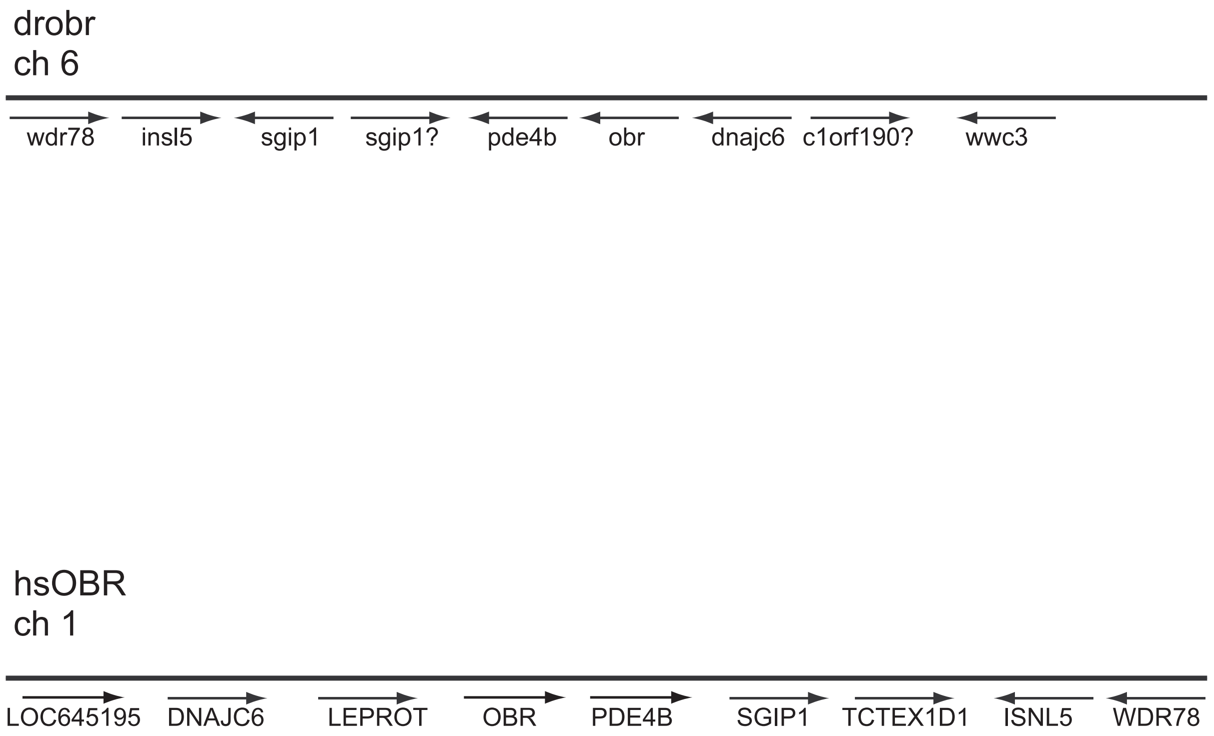
drobr
ch 6
wdr78
insl5
sgip1
sgip1?
pde4b
obr
dnajc6
c1orf190?
wwc3
hsOBR
ch 1
LOC645195
DNAJC6
LEPROT
OBR
PDE4B
SGIP1
TCTEX1D1
ISNL5
WDR78

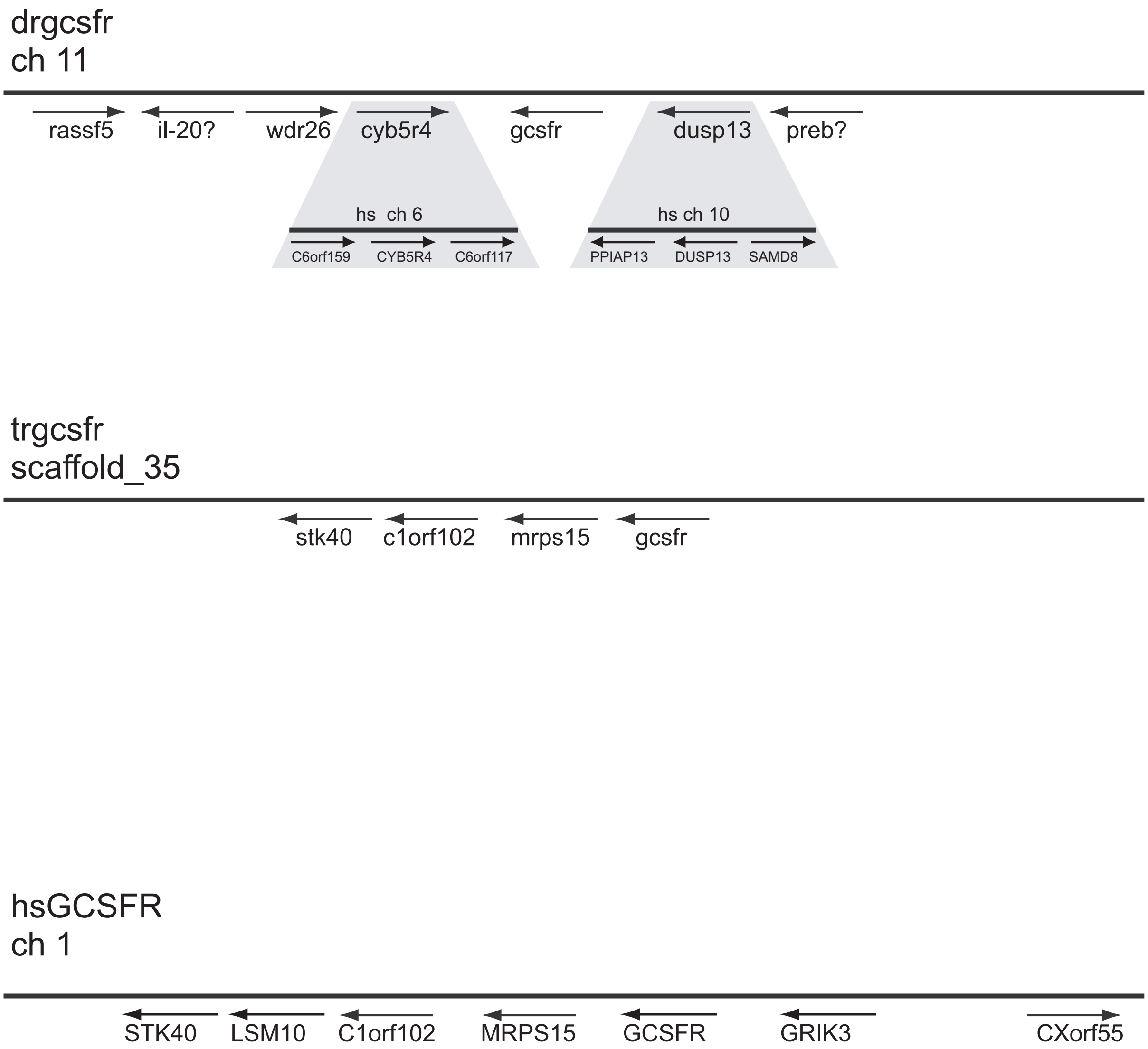
drgcsfr
ch 11
rassf5
il-20?
wdr26
cyb5r4
gcsfr
dusp13
preb?
hs ch 6
C6orf159
CYB5R4
C6orf117
hs ch 10
PPIAP13
DUSP13
SAMD8
trgcsfr
scaffold_35
stk40
c1orf102
mrps15
gcsfr
hsGCSFR
ch 1
STK40
LSM10
C1orf102
MRPS15
GCSFR
GRIK3
CXorf55

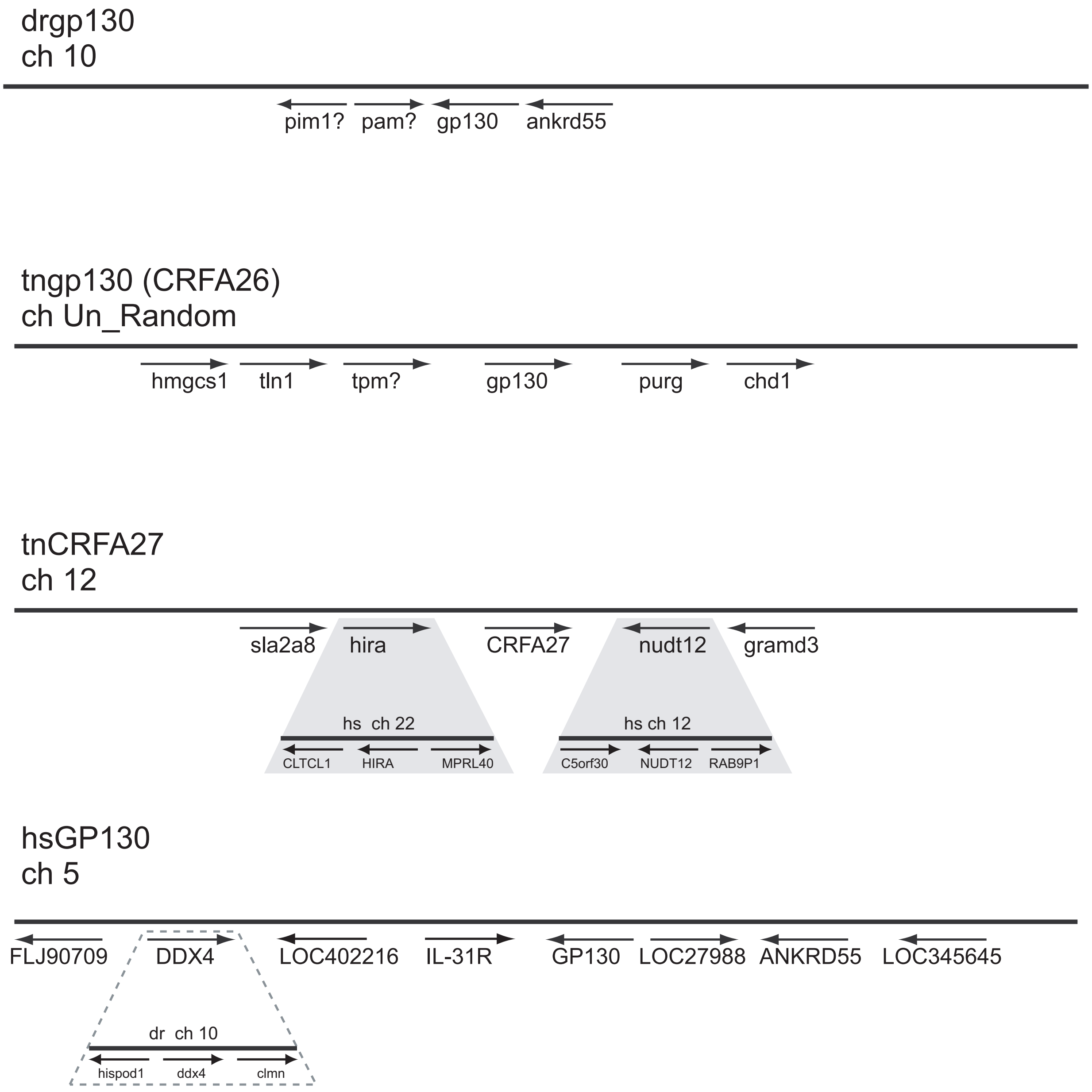
drgp130
ch 10
pim1?
pam?
gp130
ankrd55
tngp130 (CRFA26)
ch Un_Random
hmgcs1
tln1
tpm?
gp130
purg
chd1
tnCRFA27
ch 12
sla2a8
hira
CRFA27
nudt12
gramd3
hs ch 22
CLTCL1
HIRA
MPRL40
hs ch 12
C5orf30
NUDT12
RAB9P1
hsGP130
ch 5
FLJ90709
DDX4
LOC402216
IL-31R
GP130
LOC27988
ANKRD55
LOC345645
dr ch 10
hispod1
ddx4
clmn

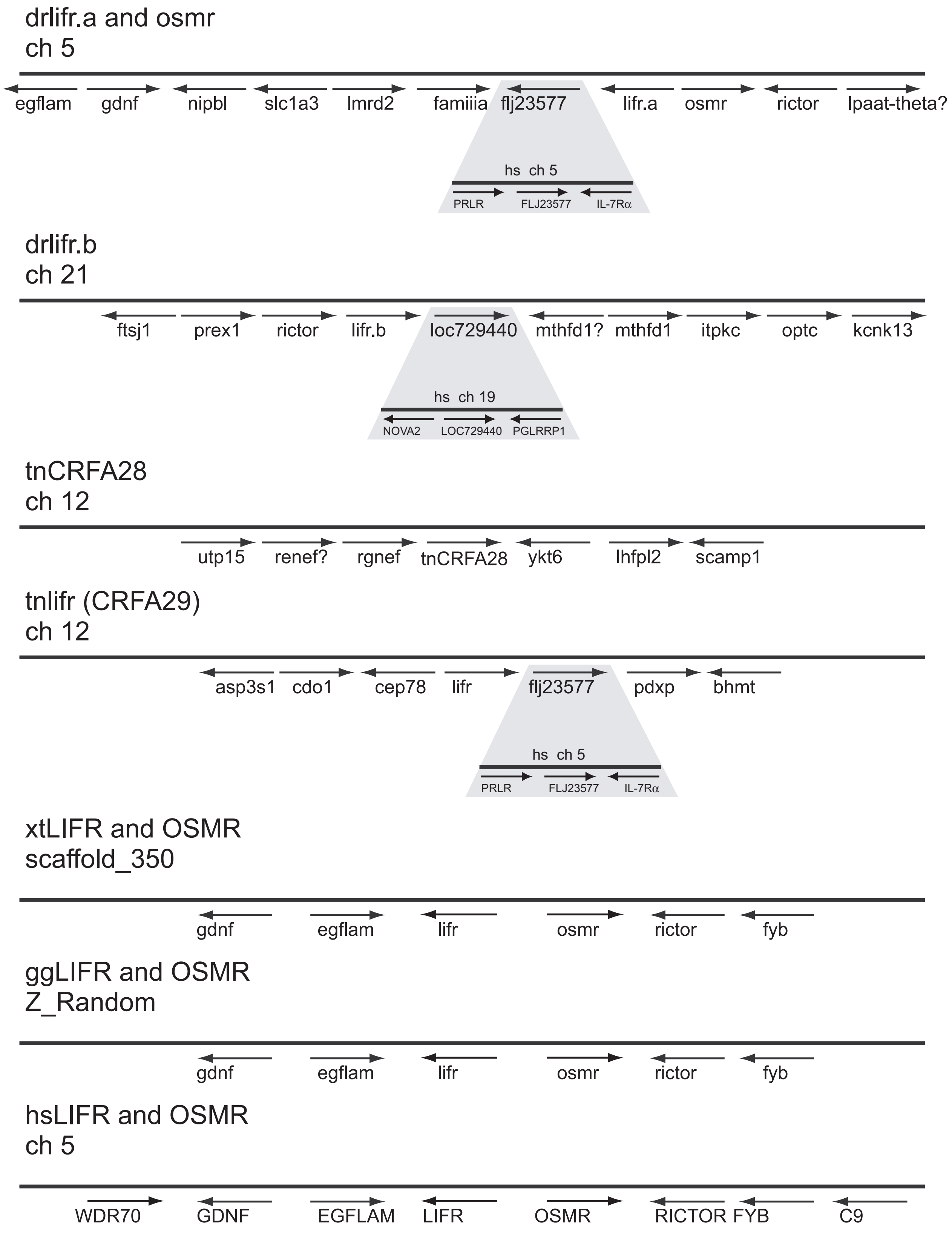
drlifr.a and osmr
ch 5
egflam
gdnf
nipbl
slc1a3
lmrd2
famiiia
flj23577
lifr.a
osmr
rictor
lpaat-theta?
hs ch 5
PRLR
FLJ23577
IL-7Rα
drlifr.b
ch 21
ftsj1
prex1
rictor
lifr.b
loc729440
mthfd1?
mthfd1
itpkc
optc
kcnk13
hs ch 19
NOVA2
LOC729440
PGLRRP1
tnCRFA28
ch 12
utp15
renef?
rgnef
tnCRFA28
ykt6
lhfpl2
scamp1
tnlifr (CRFA29)
ch 12
asp3s1
cdo1
cep78
lifr
flj23577
pdxp
bhmt
hs ch 5
PRLR
FLJ23577
IL-7Rα
xtLIFR and OSMR
scaffold_350
gdnf
egflam
lifr
osmr
rictor
fyb
ggLIFR and OSMR
Z_Random
gdnf
egflam
lifr
osmr
rictor
fyb
hsLIFR and OSMR
ch 5
WDR70
GDNF
EGFLAM
LIFR
OSMR
RICTOR
FYB
C9

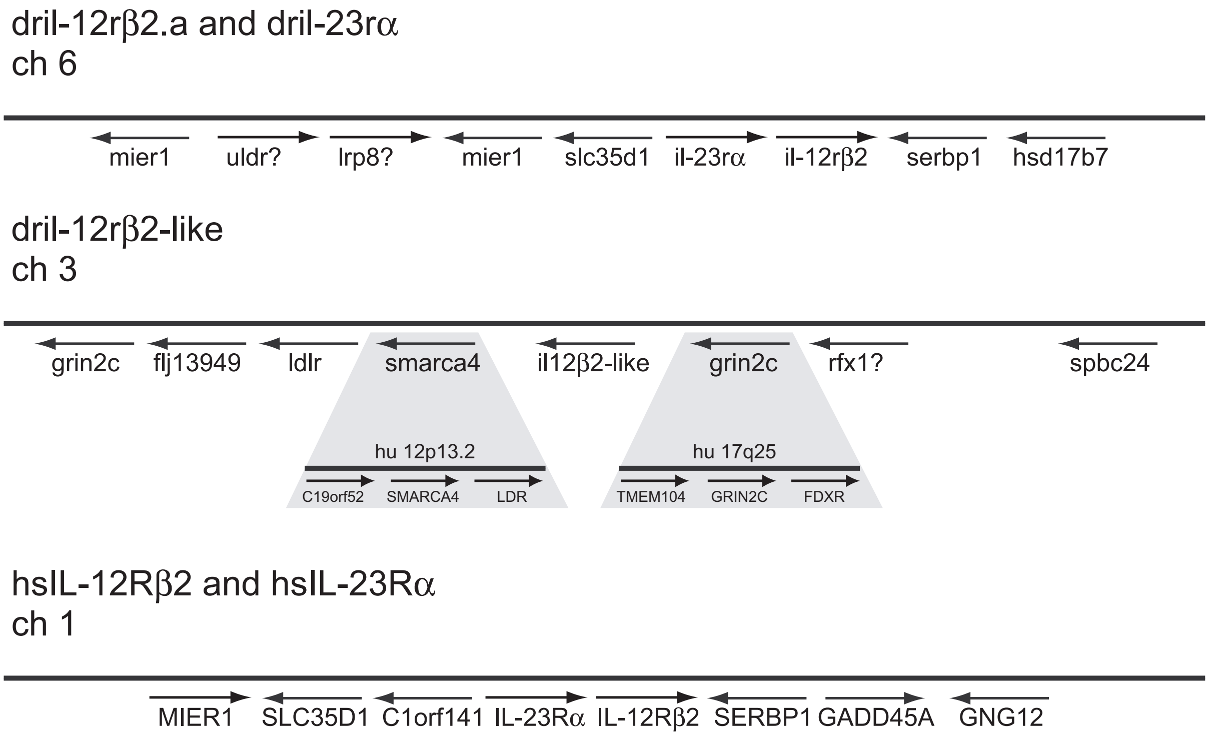
dril-12rβ2.a and dril-23rα
ch 6
mier1
uldr?
lrp8?
mier1
slc35d1
il-23rα
il-12rβ2
serbp1
hsd17b7
dril-12rβ2-like
ch 3
grin2c
flj13949
ldlr
smarca4
il12β2-like
grin2c
rfx1?
spbc24
hu 12p13.2
C19orf52
SMARCA4
LDR
hu 17q25
TMEM104
GRIN2C
FDXR
hsIL-12Rβ2 and hsIL-23Rα
ch 1
MIER1
SLC35D1
C1orf141
IL-23Rα
IL-12Rβ2
SERBP1
GADD45A
GNG12

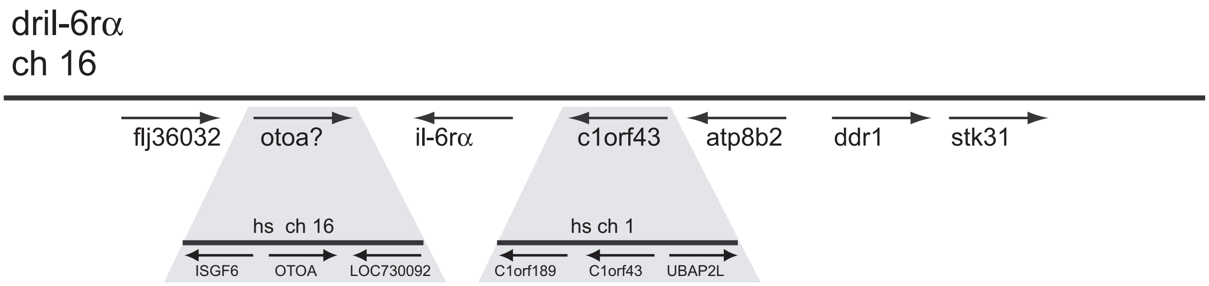
dril-6rα
ch 16
flj36032
otoa?
il-6rα
c1orf43
atp8b2
ddr1
stk31
hs ch 16
ISGF6
OTOA
LOC730092
hs ch 1
C1orf189
C1orf43
UBAP2L

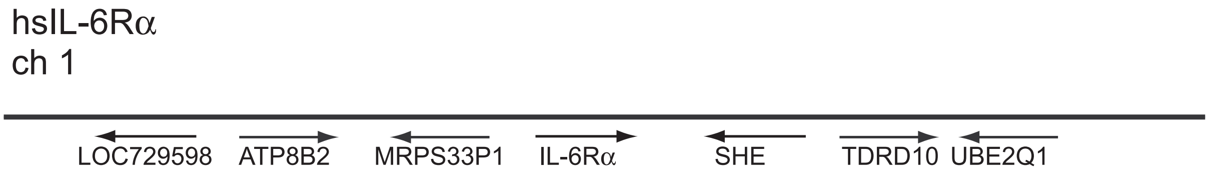
hsIL-6Rα
ch 1
LOC729598
ATP8B2
MRPS33P1
IL-6Rα
SHE
TDRD10
UBE2Q1

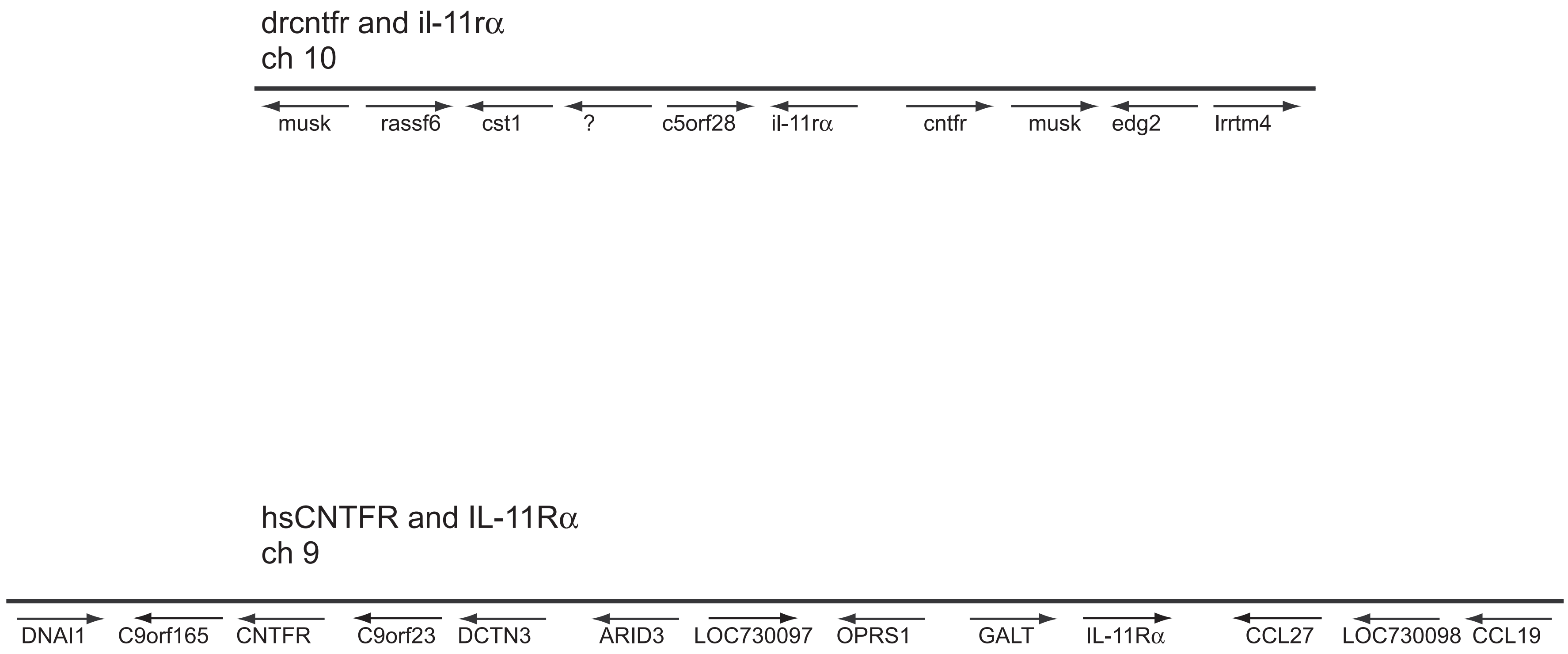
drcntfr and il-11rα
ch 10
musk
rassf6
cst1
?
c5orf28
il-11rα
cntfr
musk
edg2
lrrtm4
hsCNTFR and IL-11Rα
ch 9
DNAI1
C9orf165
CNTFR
C9orf23
DCTN3
ARID3
LOC730097
OPRS1
GALT
IL-11Rα
CCL27
LOC730098
CCL19

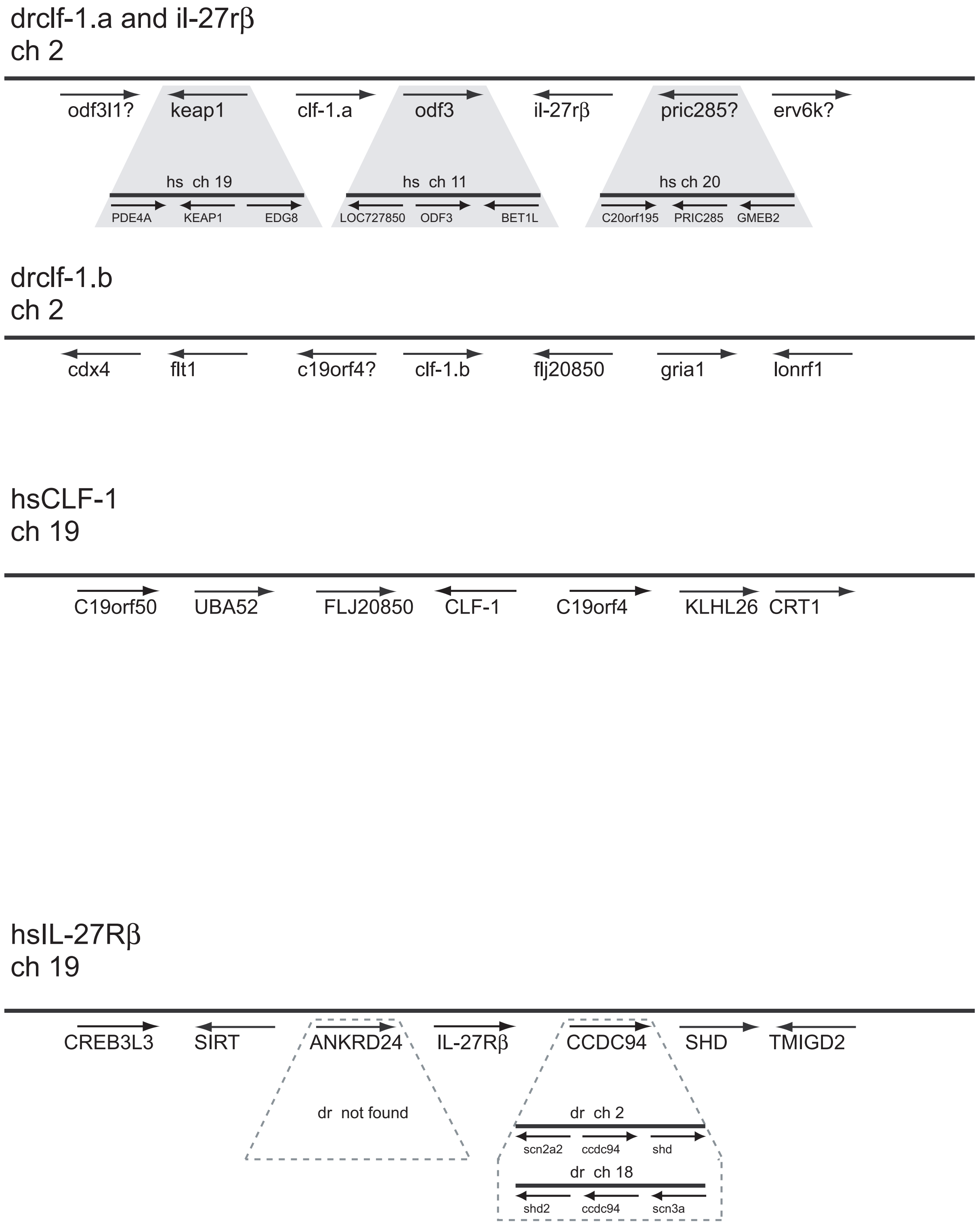

drclf-1.a and il-27rβ
ch 2
odf3l1?
keap1
clf-1.a
odf3
il-27rβ
pric285?
erv6k?
hs ch 19
PDE4A
KEAP1
EDG8
hs ch 11
LOC727850
ODF3
BET1L
hs ch 20
C20orf195
PRIC285
GMEB2
drclf-1.b
ch 2
cdx4
flt1
c19orf4?
clf-1.b
flj20850
gria1
lonrf1
hsCLF-1
ch 19
C19orf50
UBA52
FLJ20850
CLF-1
C19orf4
KLHL26
CRT1
hsIL-27Rβ
ch 19
CREB3L3
SIRT
ANKRD24
IL-27Rβ
CCDC94
SHD
TMIGD2
dr not found
dr ch 2
scn2a2
ccdc94
shd
dr ch 18
shd2
ccdc94
scn3a

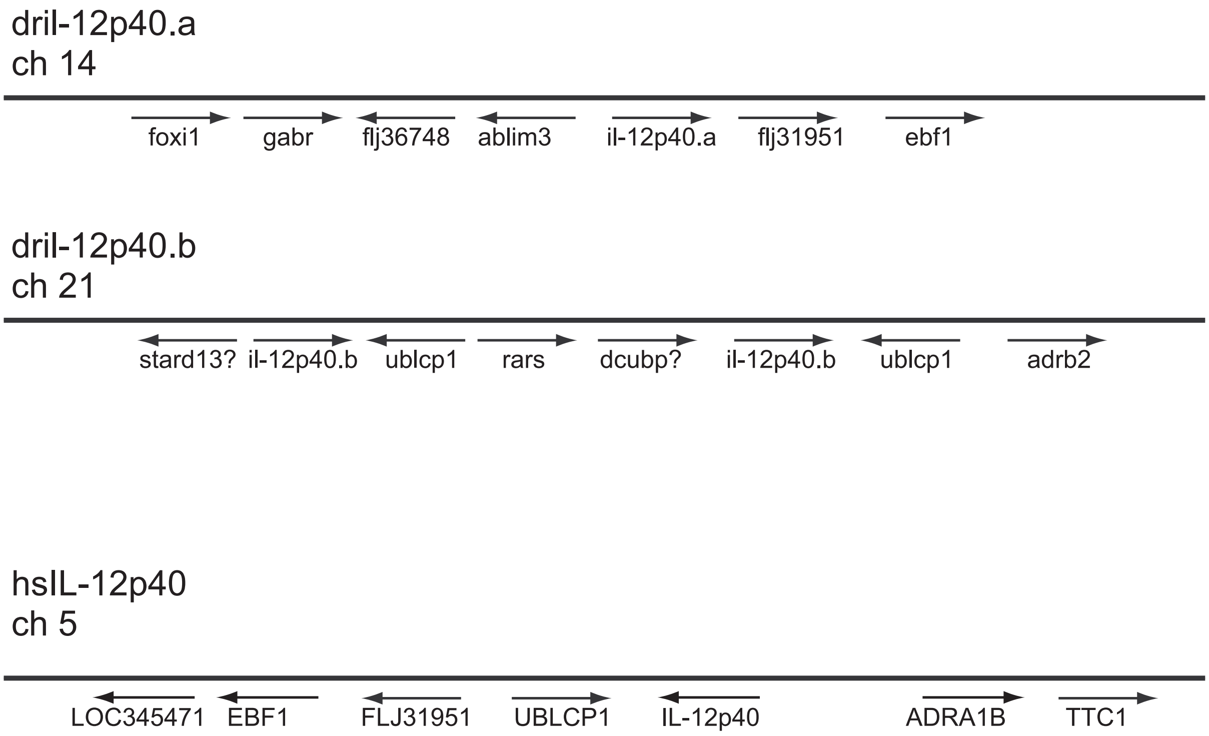
dril-12p40.a
ch 14
foxi1
gabr
flj36748
ablim3
il-12p40.a
flj31951
ebf1
dril-12p40.b
ch 21
stard13?
il-12p40.b
ublcp1
rars
dcubp?
il-12p40.b
ublcp1
adrb2
hsIL-12p40
ch 5
LOC345471
EBF1
FLJ31951
UBLCP1
IL-12p40
ADRA1B
TTC1

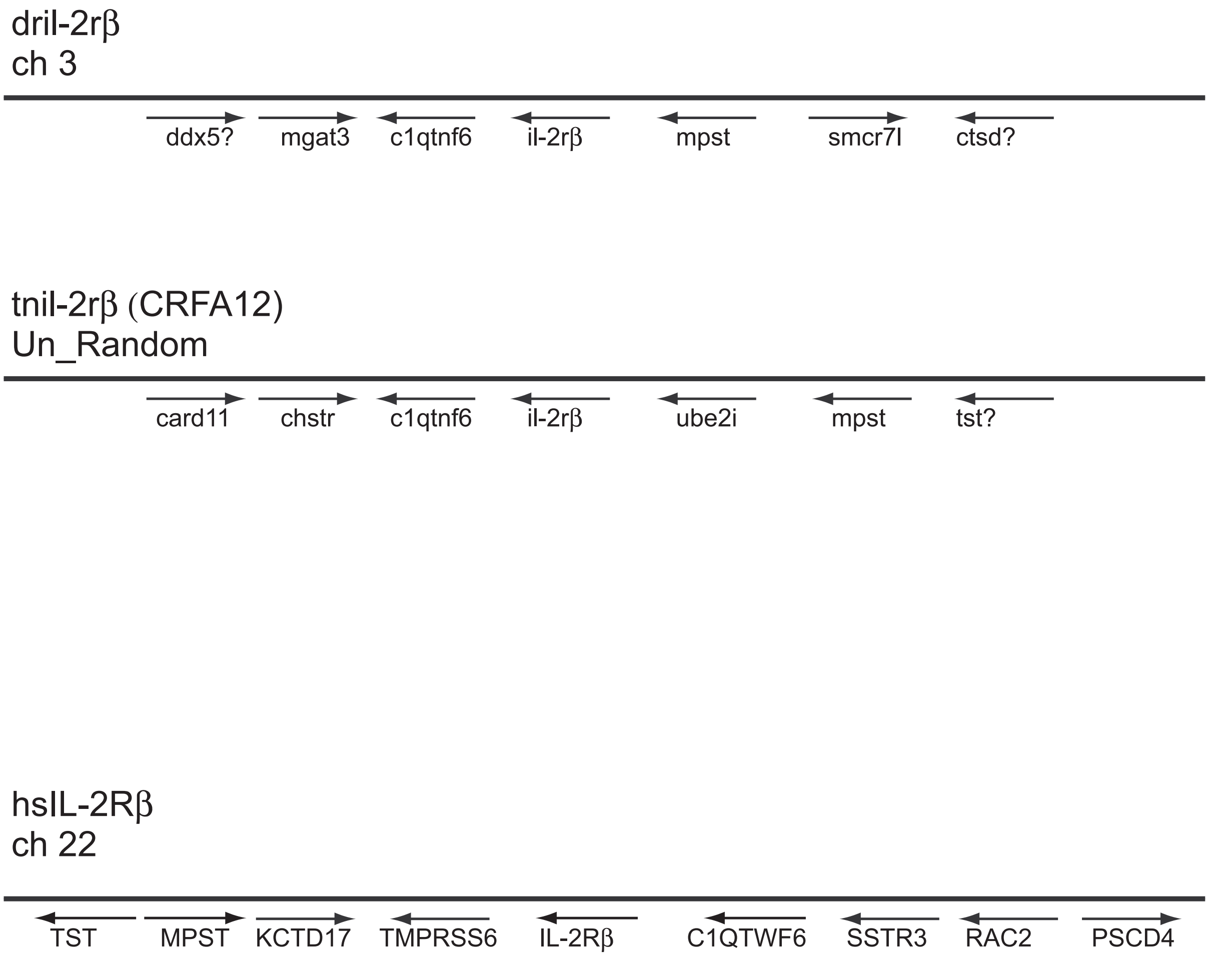
dril-2rβ
ch 3
ddx5?
mgat3
c1qtnf6
il-2rβ
mpst
smcr7l
ctsd?
tnil-2rβ (CRFA12)
Un_Random
card11
chstr
c1qtnf6
il-2rβ
ube2i
mpst
tst?
hsIL-2Rβ
ch 22
TST
MPST
KCTD17
TMPRSS6
IL-2Rβ
C1QTWF6
SSTR3
RAC2
PSCD4

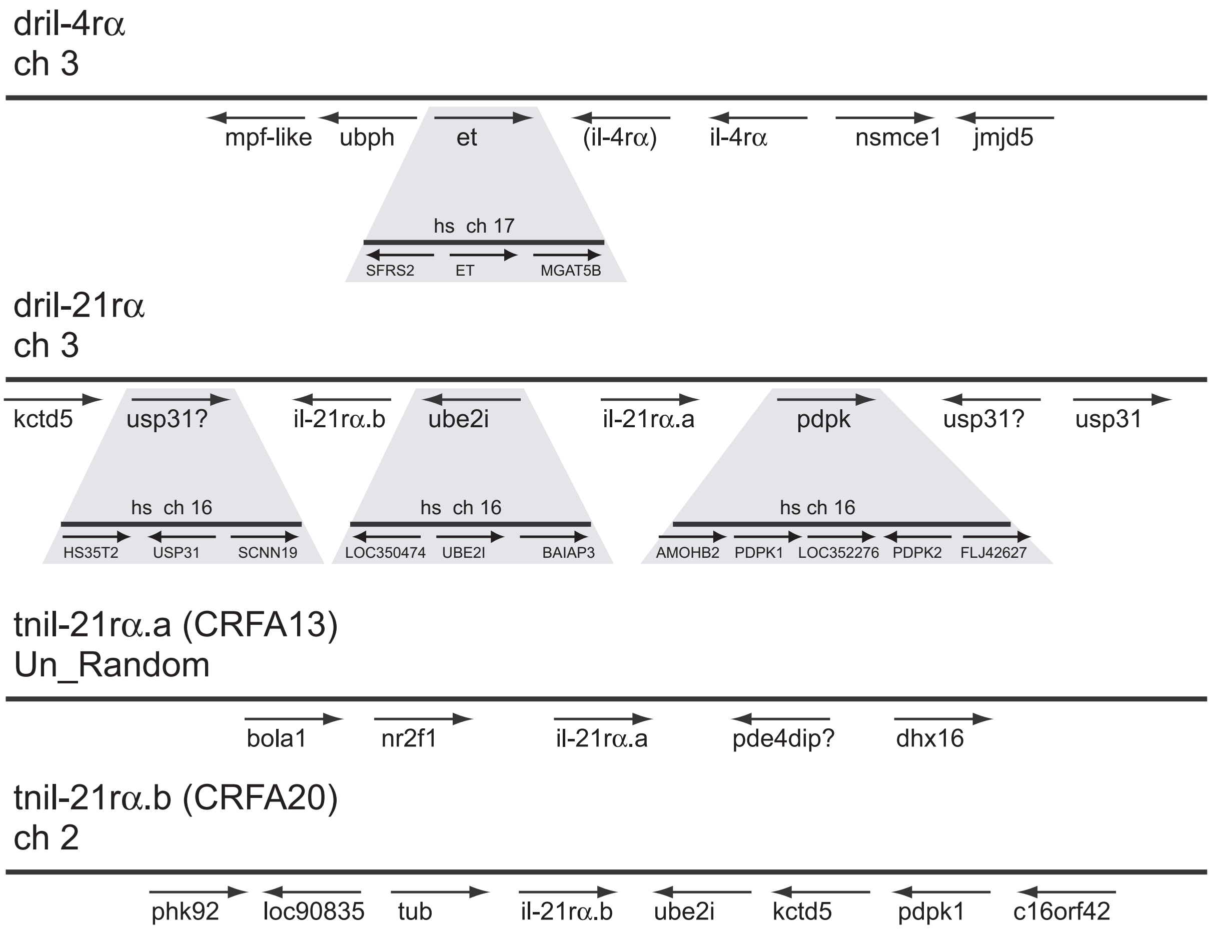
dril-4rα
ch 3
mpf-like
ubph
et
(il-4rα)
il-4rα
nsmce1
jmjd5
hs ch 17
SFRS2
ET
MGAT5B
dril-21rα
ch 3
kctd5
usp31?
il-21rα.b
ube2i
il-21rα.a
pdpk
usp31?
usp31
hs ch 16
HS35T2
USP31
SCNN19
hs ch 16
LOC350474
UBE2I
BAIAP3
hs ch 16
AMOHB2
PDPK1
LOC352276
PDPK2
FLJ42627
tnil-21rα.a (CRFA13)
Un_Random
bola1
nr2f1
il-21rα.a
pde4dip?
dhx16
tnil-21rα.b (CRFA20)
ch 2
phk92
loc90835
tub
il-21rα.b
ube2i
kctd5
pdpk1
c16orf42

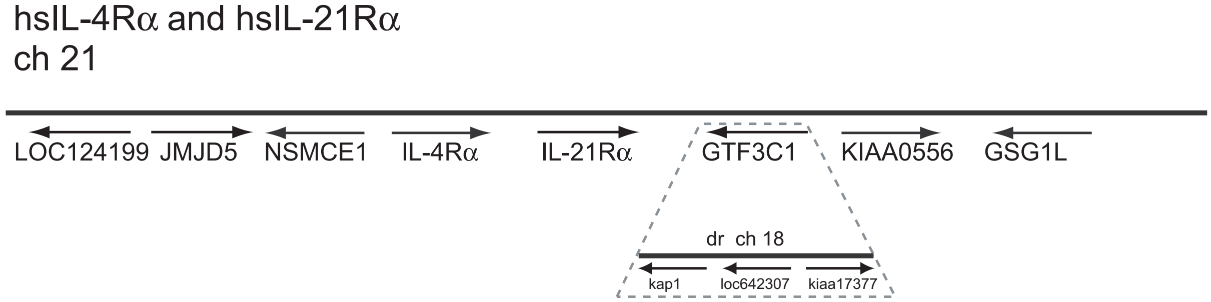
hsIL-4Rα and hsIL-21Rα
ch 21
LOC124199
JMJD5
NSMCE1
IL-4Rα
IL-21Rα
GTF3C1
KIAA0556
GSG1L
dr ch 18
kap1
loc642307
kiaa17377

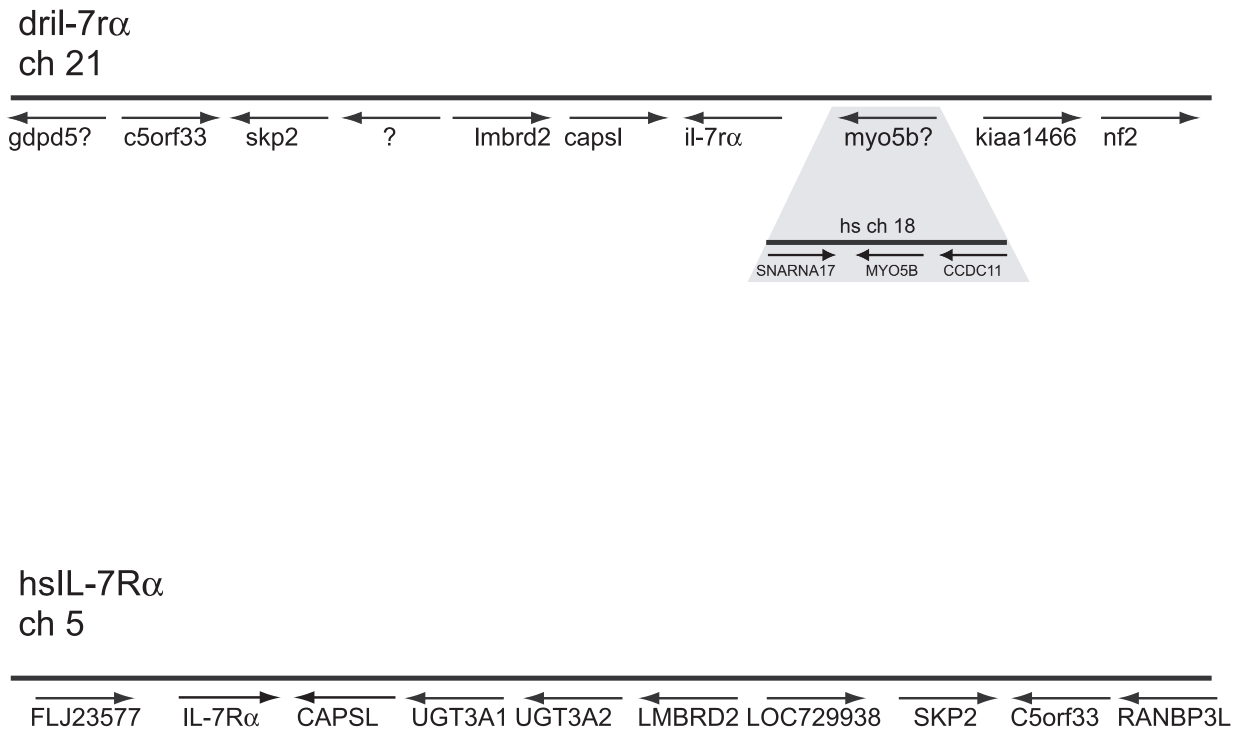

dril-7rα
ch 21
gdpd5?
c5orf33
skp2
?
lmbrd2
capsl
il-7rα
myo5b?
kiaa1466
nf2
hs ch 18
SNARNA17
MYO5B
CCDC11
hsIL-7Rα
ch 5
FLJ23577
IL-7Rα
CAPSL
UGT3A1
UGT3A2
LMBRD2
LOC729938
SKP2
C5orf33
RANBP3L

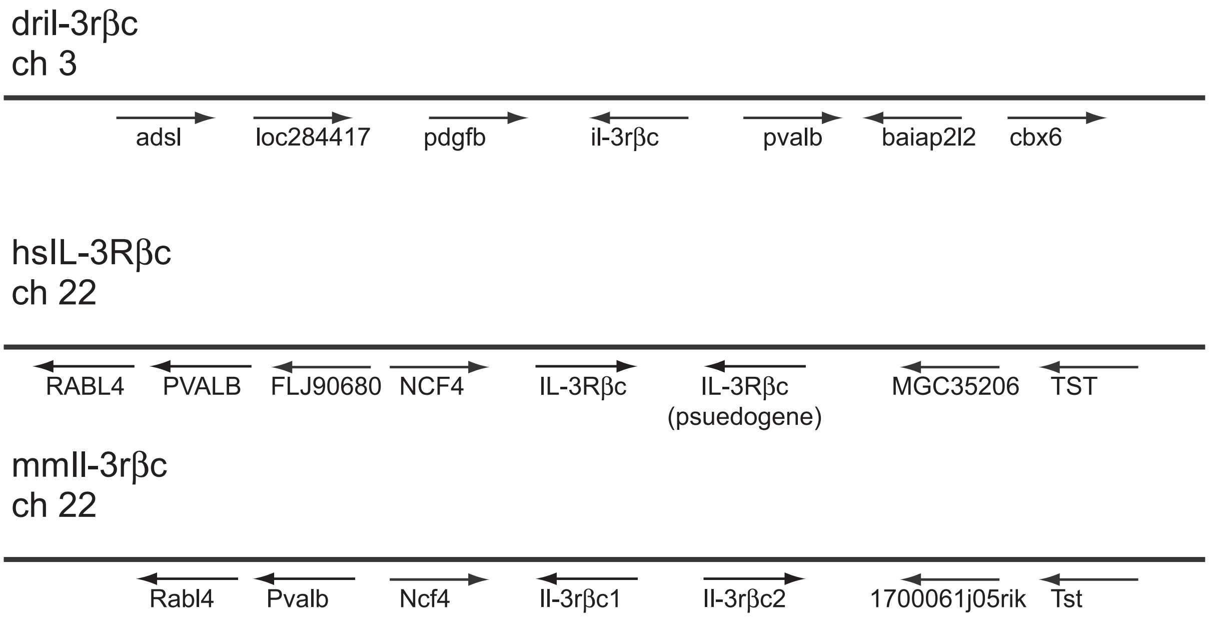

drIl-3rβc
ch 3
adsl
loc284417
pdgfb
il-3rβc
pvalb
baiap2l2
cbx6
hsIL-3Rβc
ch 22
RABL4
PVALB
FLJ90680
NCF4
IL-3Rβc
IL-3Rβc
(psuedogene)
MGC35206
TST
mmIl-3rβc
ch 22
Rabl4
Pvalb
Ncf4
Il-3rβc1
Il-3rβc2
1700061j05rik
Tst

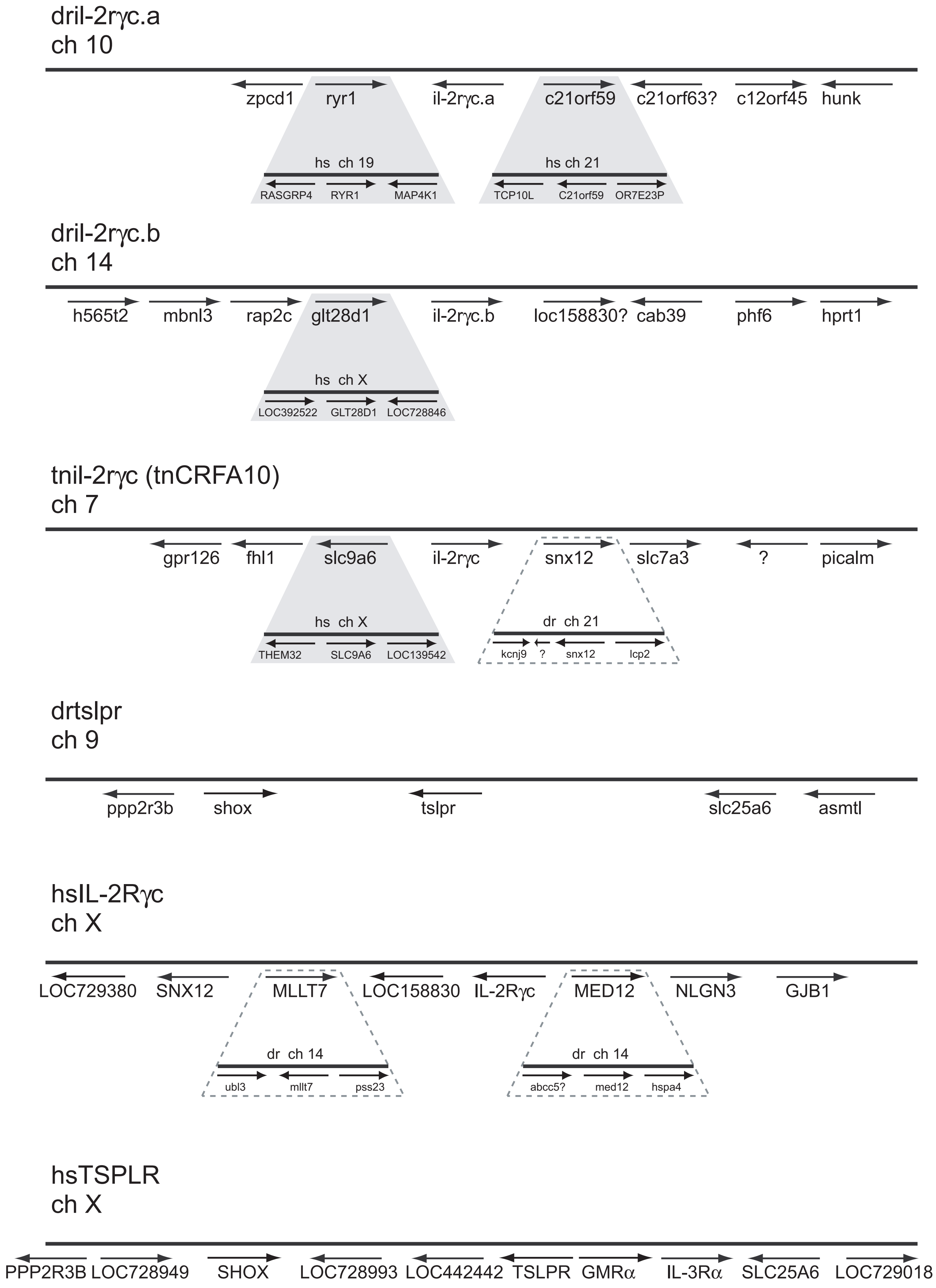

dril-2rγc.a
ch 10
zpcd1
ryr1
il-2rγc.a
c21orf59
c21orf63?
c12orf45
hunk
hs ch 19
RASGRP4
RYR1
MAP4K1
hs ch 21
TCP10L
C21orf59
OR7E23P
dril-2rγc.b
ch 14
h565t2
mbnl3
rap2c
glt28d1
il-2rγc.b
loc158830?
cab39
phf6
hprt1
hs ch X
LOC392522
GLT28D1
LOC728846
tnil-2rγc (tnCRFA10)
ch 7
gpr126
fhl1
slc9a6
il-2rγc
snx12
slc7a3
?
picalm
hs ch X
THEM32
SLC9A6
LOC139542
dr ch 21
kcnj9
?
snx12
lcp2
drtslpr
ch 9
ppp2r3b
shox
tslpr
slc25a6
asmtl
hsIL-2Rγc
ch X
LOC729380
SNX12
MLLT7
LOC158830
IL-2Rγc
MED12
NLGN3
GJB1
dr ch 14
ubl3
mllt7
pss23
dr ch 14
abcc5?
med12
hspa4
hsTSPLR
ch X
PPP2R3B
LOC728949
SHOX
LOC728993
LOC442442
TSLPR
GMRα
IL-3Rα
SLC25A6
LOC729018

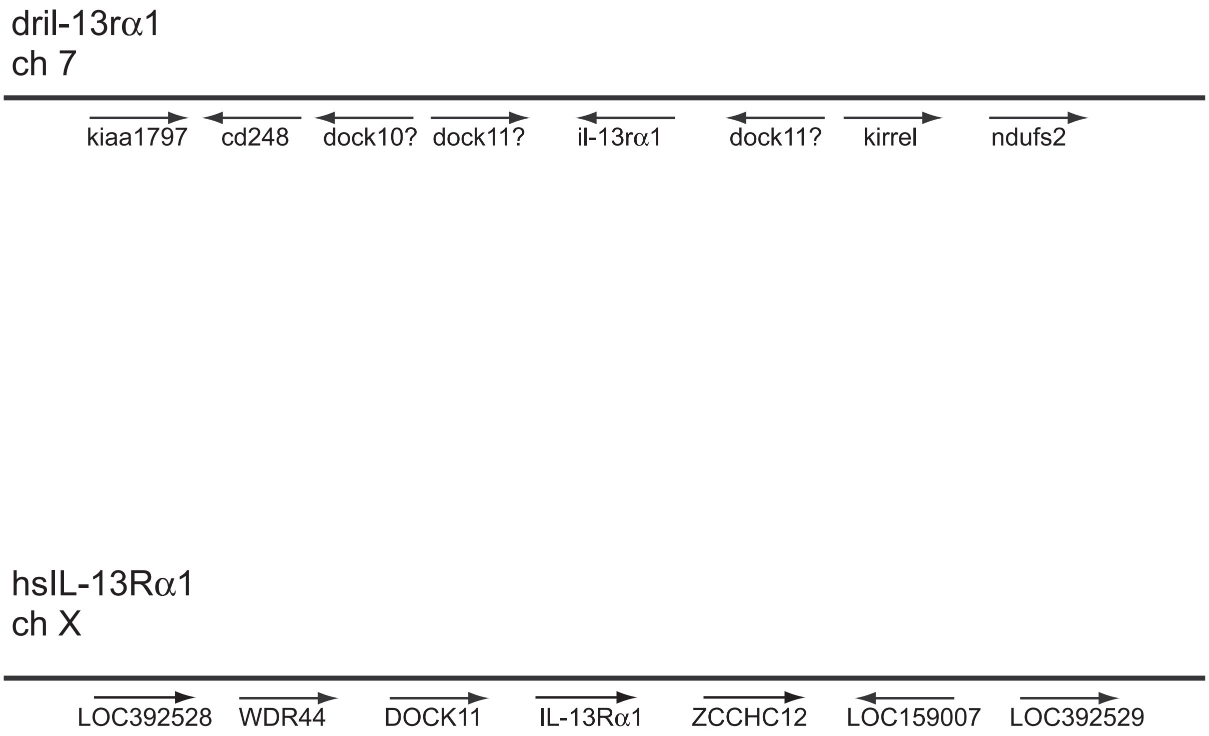

dril-13rα1
ch 7
kiaa1797
cd248
dock10?
dock11?
il-13rα1
dock11?
kirrel
ndufs2
hsIL-13Rα1
ch X
LOC392528
WDR44
DOCK11
IL-13Rα1
ZCCHC12
LOC159007
LOC392529

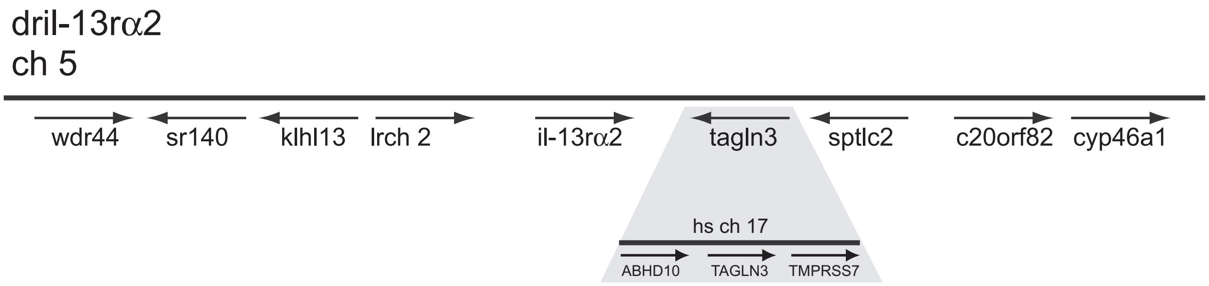

dril-13rα2
ch 5
wdr44
sr140
klhl13
lrch 2
il-13rα2
tagln3
sptlc2
c20orf82
cyp46a1
hs ch 17
ABHD10
TAGLN3
TMPRSS7

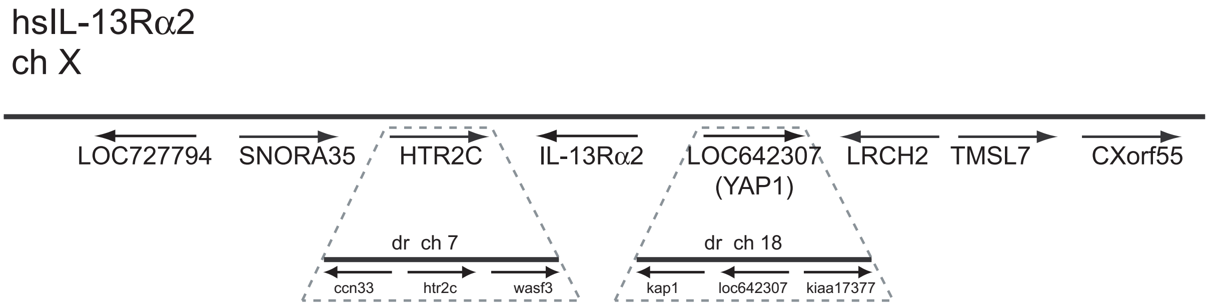

hsIL-13Rα2
ch X
LOC727794
SNORA35
HTR2C
IL-13Rα2
LOC642307
(YAP1)
LRCH2
TMSL7
CXorf55
dr ch 7
ccn33
htr2c
wasf3
dr ch 18
kap1
loc642307
kiaa17377

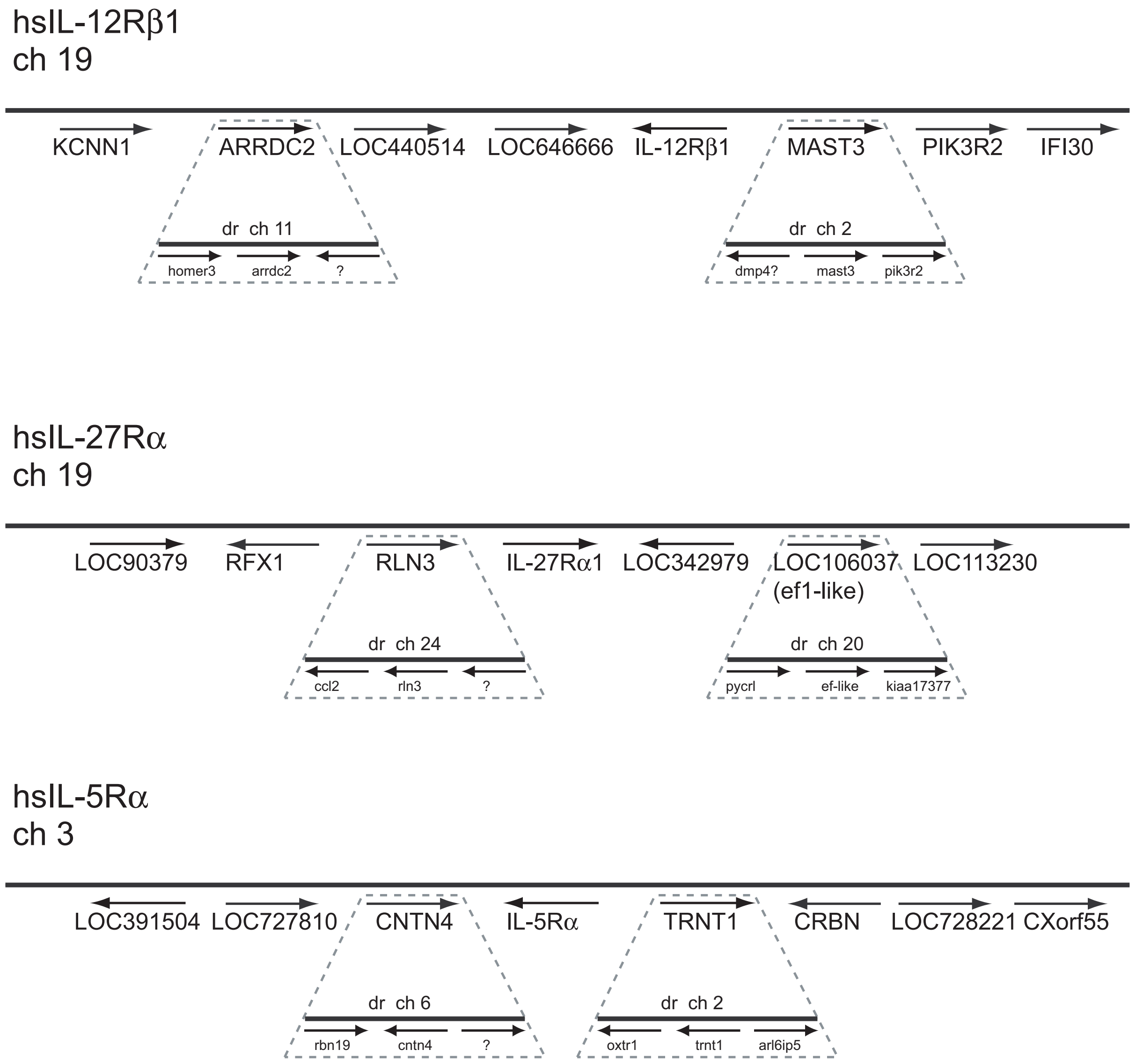
hsIL-12Rβ1
ch 19
KCNN1
ARRDC2
LOC440514
LOC646666
IL-12Rβ1
MAST3
PIK3R2
IFI30
dr ch 11
homer3
arrdc2
?
dr ch 2
dmp4?
mast3
pik3r2
hsIL-27Rα
ch 19
LOC90379
RFX1
RLN3
IL-27Rα1
LOC342979
LOC106037
(ef1-like)
LOC113230
dr ch 24
ccl2
rln3
?
dr ch 20
pycrl
ef-like
kiaa17377
hsIL-5Rα
ch 3
LOC391504
LOC727810
CNTN4
IL-5Rα
TRNT1
CRBN
LOC728221
CXorf55
dr ch 6
rbn19
cntn4
?
dr ch 2
oxtr1
trnt1
arl6ip5
