# Supplementary material for: Evolution of Class I cytokine receptors
Source: BMC Evol Biol. 2007 Jul 18;7:120. doi: 10.1186/1471-2148-7-120 (PMC1963337; doi:10.1186/1471-2148-7-120)
Supplement: Additional file 4 — Alignment of the CHD of group 1 receptors. Additional file is a pdf document that contains supplementary data. Included is an alignment of group 1 Class I cytokine receptors that were used to calculate the phylogenetic tree in Figure 3a. [file 1471-2148-7-120-S4.pdf]

CLUSTAL X (1.83) multiple sequence alignment

```
drslr            CRSSEQVTFRCWWSSGTFQNLSEPGALRIFYQT-----------KALSSDWYECPDYTQT
drghr            CFSRDMITFRCQWETGSSQNRTELEDLSFFYMLEKDP-------KKTYGKWIECPSYN-S
hsGHR            CRSPERETFSCHWTDEVHHGTKNLGPIQLFYTRRNTQ--------EWTQEWKECPDYVSA
mmGhr            CRSPELETFSCYWTEGDNPDLKTPGSIQLYYAKRESQRQAARIAHEWTQEWKECPDYVSA
drCRFA4          CRSPNMEVFSCWWRPVEKQSNVS---YTLLYSTG-------------ERAPQECPDYVSA
drprlr.a         CRSPEKETFTCWWEPGDDGGLPTT--YALYYRLEN------------TETVKECPDYKTA
drprlr.b         CRSPEKETFSCWWETGSSGGLPTR--YQLYYRKEN------------SLEVFECPDYHKA
hsPRLR           CRSPNKETFTCWWRPGTDGGLPTN--YSLTYHREG------------ETLMHECPDYITG
mmPrlr           CRSPDKETFTCWWNPGSDGGLPTN--YSLTYSKEG------------EKNTYECPDYKTS
hsTPOR_CHD2      CFTLDLKNVTCQWQQQDHASSQGFFYHSRARCCPRDRYP---IWENCEEEEKTNPGLQTP
mmTpor_CHD1      CFTLDLKMVTCQWQQQDRTSSQGFFRHSRTRCCPTDRDP---TWEKCEEEE-PRPGSQPA
hsEPOR           CFTERLEDLVCFWEEAASAGVGPG-NYSFSYQLEDEPWK--------LCRLHQAPTARGA
mmEpor           CFTQRLEDLVCFWEEAASSGMD-F-NYSFSYQLEGESRK--------SCSLHQAPTVRGS
hsTPOR_CHD1      CFSRTFEDLTCFWDEEEAAPSG---TYQLLYAYPREKPR--------ACPLSSQSMPHFG
mmTpor_CHD1      CFSQTFEDLTCFWDEEEAAPSG---TYQLLYAYRGEKPR--------ACPLYSQSVPTFG
hsCLF-3          CQKLIEHGVNTAEDLVREGEIAMLG--------------------GVGEENEKLWSFTK
mmClf-3          CQKLIEHGVNTADDLVREGEIAILG--------------------GIEEESDKLWNFTK
drclf-3          CQKLIEHGVSTADELLREGEAAIRC--------------------SINETEDKLGSFTK
drepor           CFVEGKKDFTCFWEKEDGTNYSQD-NYTFTYTYMNE-----------NKMDCAVSSLFLL
drtpor_CHD1      CFTRNLEDFTCFWDAPVEKSYDFLYIIEGLMEE--------------KRCDVKQQTFEEK
drtpor_CHD2      CHTSDLIQIICKWRGDLYKDNTYSFYYKQLNRSS-------------WSSWKLCPNCNNS
                 *       .

drslr            VKNECYFNKTFTRIWTSYCIQLRSVPQN--TTYDEACFTVENIVYPDPPVGLNWTLLNVS
drghr            QRNECYFDASHTFVWYTYVIQLRSVDN----VYEEMSFSIENIVFPDPPVGLNWTLLKMA
hsGHR            GENSCYFNSSFTSIWIPYCIKLTSNGG----TVDEKCFSVDEIVQPDPPIALNWTLLNVS
mmGhr            GKNSCYFNSSYTSIWIPYCIKLTTNGD----LLDQKCFTVDEIVQPDPPIGLNWTLLNIS
drCRFA4          GPNSCFFDAKHTQVWKVYCMNVTAHTRAGPVTSHRYCLDVADIVEPDPPFNLTYTLLNDS
drprlr.a         GENSCFFDKNATSLWVNYNITVVATNALGRNISEPVEVDVVYIVQPNTPENVKAVVIH--
drprlr.b         GINSCHFEKRHTAIWIIYNITVVASNALGKAYSEPVEVDVMDIVQPHPPESVNVSLMQ--
hsPRLR           GPNSCHFGKQYTSMWRTYIMMVNATNQMGSSFSDELYVDVTYIVQPDPPLELAVEVKQP-
mmPrlr           GPNSCFFSKQYTSIWKIYIITVNATNEMGSSTSDPLYVDVTYIVEPEPPRNLTLEVKQL-
hsTPOR_CHD2      QFSRCHFKSRNDSIIHILVEVTTAPGTVHSYLGSPFWIHQAVRL---PTPNLHWREIS--
mmTpor_CHD1      LVSRCHFKSRNDSVIHILVEVTTAQGAVHSYLGSPFWIHQAVLL---PTPSLHWREVS--
hsEPOR           VRFWCSLP--TADTSSFVPLELRVTAASG-APRYHRVIHINEVVLLDAPVGLVARLADE-
mmEpor           VRFWCSLP--TADTSSFVPLELQVTEASG-SPRYHRIIHINEVVLLDAPAGLLARRAEE-
hsTPOR_CHD1      TRYVCQFPD-QEEVRLFFPLHLWVKNVFLNQTRTQRVLFVDSVGLPAPPSIIKAMGGSQ-
mmTpor_CHD1      TRYVCQFPA-QDEVRLFFPLHLWVKNVSLNQTLIQRVLFVDSVGLPAPPRVIKARGGSQ-
hsCLF-3          KASHIQLDSLPEVPLLVDVPCLSAQLDDSILNIVKDHIFKHGTVASRPPVQIEELIEKP-
mmClf-3          KASHIQLDSLPEVPLLVDVPCLSAQLDDSILNIVKDHIFKHGTVASRPPVQIEELIEKP-
drclf-3          KALQIQLDSLPEVPSLVDVPCLSAQLDDSLLHMFRAHVARHGSVASHPPVQIEELVERP-
drepor           SSNRSVFFCKLPKALFFTSLDVQVLRDG--QMLYNRSLNVENILLTDPPRNVTVWSSGK-
drtpor_CHD1      ILNICTFPP--SDVYLYVETHIRVIDKDTNTTIYSRAVSVEDQLLLYPPSNISLHPTEE-
drtpor_CHD2      IHQCVLYGQKSNVFKFYLNTGLQPFSR------TFYAETFYMNIQTRPPEGLKVQIGEE-
                                                                ..  :
```

```
drslr            RSGLHFDVLVRWTPPPSADVKT--GWMSLVYQLQYR-----VKNNTY-------------
drghr            SSGLHCDVVLSWDPPPSAGVPLSDGWISLVYETQYR-----EKDSDQ-------------
hsGHR            LTGIHADIQVRWEAPRNADIQK--GWMVLEYELQYK-----EVNETK-------------
mmGhr            LTGIRGDIQVSWQPPPNADVLK--GWIILEYEIQYK-----EVNESK-------------
drCRFA4          TCGSSCSVLLSWMYPIAAEVHQ--GWITLVYELRYRN----LLQPNT-------------
drprlr.a         -EDRDPFVKVSWDKPRTADTRS--GWITLHYQLR-----VKQEEDKE-------------
drprlr.b         -SQNSQYVLVKWLPPHDVDTRS--GWVTIKYEVRSKILNSRDEEDSS-------------
hsPRLR           -EDRKPYLWIKWSPPTLIDLKT--GWFTLLYEIR-----LKPEKAAE-------------
mmPrlr           -KDKKTYLWVKWLPPTITDVKT--GWFTMEYEIR-----LKSEEADE-------------
hsTPOR_CHD2      ----SGHLELEWQHPSSWAAQET------CYQLRYT-----GEGHQD-------------
mmTpor_CHD1      ----SGRLELEWQHQSSWAAQET------CYQLRYT-----GEGRED-------------
hsEPOR           ----SGHVVLRWLPPPETPMTS-----HIRYEVDVSAGNGAGSVQR--------------
mmEpor           ----GSHVVLRWLPPPGAPMTT-----HIRYEVDVSAGNRAGGTQR--------------
hsTPOR_CHD1      ----PGELQISWEEP-APEISD-----FLRYELRYGPRDPKNSTGPTVIQLIATETCCPA
mmTpor_CHD1      ----PGELQIHWEAP-APEISD-----FLRHELRYGPTDSSNATAPSVIQLLSTETCCPT
hsCLF-3          -----GGIIVRWCKVDDDFTAQ-------DYRLQFR-----KCTSNH-------------
mmClf-3          -----GGIIVRWCKVDDDFTAQ-------DYRLQFR-----KCTANH-------------
drclf-3          -----GGVLVRWCKVDDDFSPQ-------DYRLQFR-----RSNSSQ-------------
drepor           ----EGQLNVSWLPPAVKYIDDS-----LIYEVRYA-----VEDSHMG------------
drtpor_CHD1      ----VGQMLVKWKRTSNEHEIR--------YSFKNT------------------------
drtpor_CHD2      ------RLCLTWDSPFLIISKH------LMYQIRYQ-----HHEENQ-------------
                        : : *                  :

drslr            ------WEMQELE---------------------------SGTQQSIYGLHTDKEYEVRV
drghr            ------WNTLESD---------------------------KNTQAYIYGLESNTEYELRV
hsGHR            ------WKMMDPI---------------------------LTTSVPVYSLKVDKEYEVRV
mmGhr            ------WKVMGPI---------------------------WLTYCPVYSLRMDKEHEVRV
drCRFA4          ------WKVKERL---------------------------RERQVELFDLPVG-SYECVV
drprlr.a         ------WVEYDAE---------------------------RQKNYNVFSLHSDREYMVQV
drprlr.b         ------WESYSAG---------------------------KQLEFSIYSPHPGTNYVVDV
hsPRLR           ------WEIHFAG---------------------------QQTEFKILSLHPGQKYLVQV
mmPrlr           ------WEIHFTG---------------------------HQTQFKVFDLYPGQKYLVQT
hsTPOR_CHD2      ------WKVLEPP---------------------------LGARGGTLELRPRSRYRLQL
mmTpor_CHD1      ------WKVLEPS---------------------------LGARGGTLELRPRARYSLQL
hsEPOR           ------VEILEG-----------------------------RTECVLSNLRGRTRYTFAV
mmEpor           ------VEVLEG-----------------------------RTECVLSNLRGGTRYTFAV
hsTPOR_CHD1      LQRPHSASALDQSPCAQPTMPWQDGPKQTSPSREASALTAEGGSCLISGLQPGNSYWLQL
mmTpor_CHD1      LWMPNPVPVLDQPPCVHPTASQPHGP--------APFLTVKGGSCLVSGLQASKSYWLQL
hsCLF-3          ------FEDVYVG---------------------------SETEFIVLHIDPNVDYQFRV
mmClf-3          ------FEDVYVG---------------------------SETEFIVLHIDPNVDYQFRV
drclf-3          ------YEDAYIG---------------------------KDTEFLVLHLDPHVDHLFRV
drepor           -------KVEETK---------------------------ASTMLVLRGLQPDTRYKVWV
drtpor_CHD1      ------STVKSVS----------------------------ENDYKLVSLVPGENCTVQM
drtpor_CHD2      ------WKGFKAS---------------------------GSKTSTCLDVHRGGRYTIQV
```

```
drslr            RCKMS---AFNNFGEFS
drghr            RAKMR---SFH-FGDFS
hsGHR            RSKQR---NSGNYGEFS
mmGhr            RSRQR---SFEKYSEFS
drCRFA4          RCRSA---NSRHWSEWS
drprlr.a         RCKP----DHGFWSEWT
drprlr.b         RCKL----DQGLWSDWS
hsPRLR           RCKP----DHGYWSAWS
mmPrlr           RCKP----DHGYWSRWG
hsTPOR_CHD2      RARLNGPTYQGPWSSWS
mmTpor_CHD1      RARLNGPTYQGPWSAWS
hsEPOR           RARMAEPSFGGFWSAWS
mmEpor           RARMAEPSFSGFWSAWS
hsTPOR_CHD1      RSEPDGISLGGSWGSWS
mmTpor_CHD1      RSQPDGVSLRGSWGPWS
hsCLF-3          CARGD---GRQEWSPWS
mmClf-3          CARGD---GRQEWSPWS
drclf-3          CARGE---GRTEWSPWS
drepor           RVKPDGVSYKGYWSSWT
drtpor_CHD1      RVKPSG-DFKRFWSDWS
drtpor_CHD2      RAQPNGSVYSGNWSDWS
                   .         :. :
```
