# Supplementary material for: Evolution of Class I cytokine receptors
Source: BMC Evol Biol. 2007 Jul 18;7:120. doi: 10.1186/1471-2148-7-120 (PMC1963337; doi:10.1186/1471-2148-7-120)
Supplement: Additional file 5 — Alignment of the CHD of group 2 receptors. Additional file is a pdf document that contains supplementary data. Included is an alignment of group 2 Class I cytokine receptors that were used to calculate the phylogenetic tree in Figure 3b. [file 1471-2148-7-120-S5.pdf]

CLUSTAL X (1.83) multiple sequence alignment

```
hsIL-12Rb2          -CIQKGEQG----TVACTWERGR-DTHLYTEYTLQLSG------------PKNLTWQKQC
mmIl-12rb2          -CVQEGENG----TVACSWNSGK-VTYLKTNYTLQLSG------------PNNLTCQKQC
hsLIFR_CHD1         -CVTNNLQ-----VWNCSWKAPS-GTGRGTDYEVCIEN--------RSRSCYQLEK--TS
mmLifr_CHD1         -CTTNNMR-----VWDCTWPAPL-GVSPGTVKDICIKD--------RFHSCHPLET--TN
hsOSMR_CHD0.5       ------------------------------------------------------------
mmOsmr_CHD0.5       ------------------------------------------------------------
hsIL-12b1           -CYRISSD-----RYECSWQYEG-PTAGVSHFLRCCLS------SG--RCCYFAAG--SA
mmIl-12b1           -CYRVSKT-----DYECSWQYDG-PEDNVSHVLWCCFVPPNHTHTGQERCRYFSSG--PD
hsOBR_CHD1          -CWLKGDL-----KLFICYVESL-FKNLFRNYNYKVHLLYVLPEVLEDSPLVPQKGSFQM
mmObr_CHD1          -CWMKGDL-----TLFICHMEPL-PKNPFKNYDSKVHLLYDLPEVIDDSPLPPLKDSFQT
hsOBR_CHD2          -CETDGYL-----TKMTCRWSTS-TIQSLAESTLQLRYHRSSLYCSDIPSIHPIS---EP
mmObr               -CETDGYL-----TKMTCRWSPS-TIQSLVGSTVQLRYHRRSLYCPDSPSIHPTS---EP
hsGCSFR             -CLMNLTTS----SLICQWEPGP-ETHLPTSFTLKSFK-----------SRGNCQTQGDS
mmGcsfr             -CLMHLTTN----SLVCQWEPGP-ETHLPTSFILKSFR-----------SRADCQYQGDT
hsIL-23R            -CVIYEYSG----NMTCTWNAGK-LTYIDTKYVVHVKSLETEEEQQ--------------
mmIl-23r            -CVIYEYSG----NMTCTWNTGK-PTYIDTKYIVHVKSLETEEEQQ--------------
hsOSMR_CHD2         -CETEDFK-----TLHCTWDPGT-DTALGWSKQPSQSYTLFESFSG--------------
mmOsmr_CHD2         -CETRDFK-----TLDCSWEPGV-DTTLTWRKQRFQNYTLCESFSK--------------
hsLIFR_CHD2         -CETHDLK-----EIICSWNPGR-VTALVGPRA--TSYTLVESFSGK-----------YV
mmLifr_CHD2         -CETHDLK-----EIICSWNPGR-ITGLVGPRN--TEYTLFESISGK-----------SA
drlifr.b_CHD2       -CITRDLS-----SVECQWKLGR-KTQLYG-TRA-TNYTLNGRKC---------------
drlifr.a_CHD2       -CETRDLA-----SAKCSWKKGR-DTRLKKRNSR-TNYTLNGRVCG--------------
hsGP130             -CIVNEGK-----KMRCEWDGGR-ETHLETNFTLKS-EWATHK-----------------
mmGp130             -CIVNEGK-----NMLCQWDPGR-ETYLETNYTLKS-EWATEK-----------------
hsIL-27R            CYGVGPLG-----DLNCSWEPLG-DLGAPSELHLQSQKYRSNK-----------------
mmIl-27r            CYSVGPLG-----ILNCSWEPLG-DLETPPVLYHQSQKYHPNR-----------------
hsGLMR              -CVYYYRK-----NLTCTWSPGK-ETSYTQYTVKRTYAFGEKHDNC--------------
mmGlmr              -CVFYFDR-----NLTCTWRPEK-ETNDTSYIVTLTYSYGK-------------------
drlifr.a_CHD0.5     ------------------------------------------------------------
drlifr.b_CHD0.5     ------------------------------------------------------------
drosmr_CHD0.5       ------------------------------------------------------------
drgp130             -CIALQRKKDISPDLNCYWEPGLRDPLLATNYTLYAQVGQDLYN----------------
dril-12rb2.a        -CIRKTEFG----NVSCTWTTGR-DTKIVTTCQLRVQGDPHHG-----------------
drgcsfr             -CALTLVKMP---SLRCDWNPVQEIKNLPINYTLHVFRAKSQK-----------------
drobr_CHD2          -CESEYSYVD---TMICKWNQSNWAQARLLYRQYRSKCTEEAEEDTS-------------
drosmr_CHD2         -CETADMHN-----IHCSWEILR-APNLNGSYRRTYTLLISDS-----------------
dril-23r            -CVTHQHS-----KFVNCYLTATTETYLFTNFRVTFRNRTSLLG----------------
dril-12rb2.b        -CVIEDLVK---EDIYCSWTISD-EPMITTVYTLHWQDYEGNVN----------------
drobr_CHD1          -CWLEGERE----NLICNAKTRRAAAAAASTLVSVSPHQLVVQMDVHS--------DETN
```

```
hsIL-12Rb2          KD---IYCDYLDFGINLTPESPESNFTAKVTAVNSLGSSS-----SLPSTFTFLDIVRPL
mmIl-12rb2          FSDNRQNCNRLDLGINLSPDLAESRFIVRVTAINDLGNSS-----SLPHTFTFLDIVIPL
hsLIFR_CHD1         IKIPALSHGDYEITINSLHDFGSSTSKFTLNEQNVSLIPD-----TPEILNLSADFSTST
mmLifr_CHD1         VKIPALSPGDHEVTINYLNGFQS---KFTLNEKDVSLIPE-----TPEILDLSADFFTSS
hsOSMR_CHD0.5       ------------------------------------------------------------
mmOsmr_CHD0.5       ------------------------------------------------------------
hsIL-12b1           TRLQFSDQAGVSVLYTVTLWVESWARNQTEKSPEVTLQLYNSVKYEPPLGDIKVSKLAGQ
mmIl-12b1           RTVQFWEQDGIPVLSKVNFWVESRLGNRTMKSQKISQYLYNWTKTTPPLGHIKVSQSHGQ
hsOBR_CHD1          VHCNCSVHECCECLVPVPTAKLNDTLLMCLKITSGGVIFQ-----SPLMSVQPINMVKPD
mmObr_CHD1          VQCNCSLRG-CECHVPVPRAKLNYALLMYLEITSAGVSFQ-----SPLMSLQPMLVVKPD
hsOBR_CHD2          KDCYLQSDGFYECIFQP--IFLLSGYTMWIRINHSLGSLD-----SPPTCVLPDSVVKPL
mmObr               KNCVLQRDGFYECVFQP--IFLLSGYTMWIRINHSLGSLD-----SPPTCVLPDSVVKPL
hsGCSFR             ILDCVPKDGQSHCCIPRKHLLLYQNMGIWVQAENALGTSM-----SPQLCLDPMDVVKLE
mmGcsfr             IPDCVAKKRQNNCSIPRKNLLLYQYMAIWVQAENMLGSSE-----SPKLCLDPMDVVKLE
hsIL-23R            ------YLTSSYINISTDSLQGGKKYLVWVQAANALGMEESK------QLQIHLDDIVIP
mmIl-23r            ------YLASSYVKISTDSLQGSRKYLVWVQAVNSLGMENSQ------QLHVHLDDIVIP
hsOSMR_CHD2         ---EKKLCTHKNWCNWQITQDSQETYNFTLIAENYLRKRSVN------ILFNLTHRVYLM
mmOsmr_CHD2         ---RCEVSNYRNSYTWQITEGSQEMYNFTLTAENQLRKRSVN------INFNLTHRVHPK
hsLIFR_CHD2         RLKRAEAPTNESYQLLFQMLPNQEIYNFTLNAHNPLGRSQST------ILVNITEKVYPH
mmLifr_CHD2         VFHRIEGLTNETYRLGVQMHPGQEIHNFTLTGRNPLGQAQSA------VVINVTERVAPH
drlifr.b_CHD2       -----EFNRCDPLELKKQITN------WTLIARNALGEKIVT------DTGDPTHRVWLK
drlifr.a_CHD2       -----EANEGKSWECSSEVWEK----NWTLVARNPIGTIQLT------DSAQITDRVHLL
hsGP130             FADCKAKRDTPTSCTVDYSTVYFVNIEVWVEAENALGKVTSDH-----INFDPVYKVKPN
mmGp130             FPDCQSKHG--TSCMVSYMPTYYVNIEVWVEAENALGKVSSES-----INFDPVDKVKPT
hsIL-27R            TQTVAVAAGRSWVAIPREQLTMSDKLLVWGTKA---GQPLWPP-----VFVNLETQMKPN
mmIl-27r            VWEVKVPSKQSWVTIPREQFTMADKLLIWGTQK---GRPLWSS-----VSVNLETQMKPD
hsGLMR              TTNSSTSENRASCSFFLPRITIPDNYTIEVEAENGDGVIKSHM-----TYWRLENIAKTE
mmGlmr              -SNYSDNATEASYSFPRSCAMPPDICSVEVQAQNGDGKVKSDI-----TYWHLISIAKTE
drlifr.a_CHD0.5     ------------------------------------------------------------
drlifr.b_CHD0.5     ------------------------------------------------------------
drosmr_CHD0.5       ------------------------------------------------------------
drgp130             --STCKQSLARSCTVNLSTYPVHMTLKVWVEVKNSLGSERSDE-----HEYDSLSLVKPN
dril-12rb2.a        --YESVMNSSGFCWATFPIKSRMSQLTVSLNVSNSLGSNT--------------SVQPS
drgcsfr             --VYAVPPGQHFYVVPRDAYGYFSELEISVTAANVLGNTTSDP-----LKLTPLNTVKFD
drobr_CHD2          LVKECPSKAGDHRQCTLSQISMIFCYKFWLEVEGGRGQSFP-------VYVTPIDYVKPS
drosmr_CHD2         ---RTVSCNFNPPSCSFKVVPHQITYNVTLLVRNSLGRESESD------IFNITDRVFPM
dril-23r            ----PYEINKGHISLQRSLFNESMTYNVHVIGHNALGQSLST------FNFSIWDIVIPS
dril-12rb2.b        ----SAESDCEMAIINRDKYTKGTQMTVWVTARNVLGYARSEE-----SVFNTGHIRRPD
drobr_CHD1          STAQCVGEETAICSVSLHGGDATVSLVIIISENGTTAQSQK-------MQVSTYELQAGD
```

```
hsIL-12Rb2          PPWDIRIKF-----QKASVSRCTLYW-RDEGLVLLNRLRYRPSNSRLWNMVNVTKAKGRH
mmIl-12rb2          PPWDIRINF-----LNASGSRGTLQW-EDEGQVVLNQLRYQPLNSTSWNMVNATNAKGKY
hsLIFR_CHD1         LYLKWNDRG-----SVFPHRSNVIWE-IKVLRKESMELVKLVTHNTTLNGKDTLHHWSWA
mmLifr_CHD1         LLLKWNDRG-----SALPHPSNATWE-IKVLQNPRTEPVALVLLNTMLSGKDTVQHWNWT
hsOSMR_CHD0.5       ----WTVHN-----LPYHQELKMVFQ-IQISRIETSNVIWVGNYSTTVK-WNQVLHWSWE
mmOsmr_CHD0.5       ----WTVPA-----LT-HEELNMIFQ-IEISRLNISNTIWVENYSTTVK-REEAVRWNWT
hsIL-12b1           LRMEWETPD-----NQVGAEVQFRHR-TPSSPWKLGDCGPQDDD-------------TES
mmIl-12b1           LRMDWNVS------EEAGAEVQFRRR-MPTTNWTLGDCGPQVNSGSGVL-GDICGSMSES
hsOBR_CHD1          PPLGLHMEI-----TD-DGNLKISWS-SPPLVPFPLQYQVKYSENSTTVIREADKIVSAT
mmObr_CHD1          PPLGLHMEV-----TD-DGNLKISWD-SQTMAPFPLQYQVKYLENS-TIVREAAEIVSAT
hsOBR_CHD2          PPSSVKAEI-----TINIGLLKISWE-KPVFPENNLQFQIRYGLSGKEVQWKMYEVYDAK
mmObr               PPSNVKAEI-----TVNTGLLKVSWE-KPVFPENNLQFQIRYGLSGKEIQWKTHEVFDAK
hsGCSFR             PPMLRTMDPSPEAAPPQAGCLQLCWEPWQPGLHINQKCELRHKPQRGEASWALVGPLPLE
mmGcsfr             PPMLQALDIGPDVVSHQPGCLWLSWKPWKPSEYMEQECELRYQPQLKGANWTLVFHLPSS
hsIL-23R            SAAVISRAETINATVPKTIIYWDSQTTIEK---VSCEMRYK--ATTNQTWNVKEFDTNFT
mmIl-23r            SASIISRAETTNDTVPKTIVYWKSKTMIEK---VFCEMRYK--TTTNQTWSVKEFDANFT
hsOSMR_CHD2         NPFSVNFENVN-ATNAIMTWKVHSIRNNFT---YLCQIELHGEGKMMQY-NVSIK--VNG
mmOsmr_CHD2         APQDVTLKIIG-ATKANMTWKVHSHGNNYT---LLCQVKLQYGEVIHEH-NVSVH--MSA
hsLIFR_CHD2         TPTSFKVKDIN-STAVKLSWHLPGNFAKIN---FLCEIEIKKSNSVQEQRNVTIKGVENS
mmLifr_CHD2         DPTSLKVKDIN-STVVTFSWYLPGNFTKIN---LLCQIEICKANSKKEVRNATIRGAEDS
drlifr.b_CHD2       APHNISHAAHARNATVHWSWSVT-EYASLL---MICQK------------TFSGK----G
drlifr.a_CHD2       APVNVMADPKAWNATLRWNWTVE-AYKKLE---MICQMKLI-SRQLSSTRNYSGT----G
hsGP130             PPHNLSVIN-SEELSSILKLTWTNPSIKSV-IILKYNIQYRTKDASTWSQIPPEDTAST-
mmGp130             PPYNLSVTN-SEELSSILKLSWVSSGLGGL-LDLKSDIQYRTKDASTWIQVPLEDTMSP-
hsIL-27R            APRLGPDVDFSEDDPLEATVHWAPPTWPSH-KVLICQFHYRRCQEAAWTLLEPELKTIP-
mmIl-27r            TPQIFSQVDISEEATLEATVQWAPPVWPPQ-KALTCQFRYKECQAEAWTRLEPQLKTDG-
hsGLMR              PPKIF-RVKPVLGIKRMIQIEWIKPELAPVSSDLKYTLRFRTVNSTSWMEVNFAKNRKDK
mmGlmr              PPIIL-SVNPIC--NRMFQIQW-KPREKTRGFPLVCMLRFRTVNSSRWTEVNFENCK---
drlifr.a_CHD0.5     ----WQSDS-------------SLFD-IEIFHTEFMNVVLNETVAVNTDPVTGQHSWTWH
drlifr.b_CHD0.5     ----WTDEN--------NEFEKVSYD-IEIIHTEQMRTVHFENIHVKPD-KSSHYHWKWT
drosmr_CHD0.5       ----WNINQ---------NKQRIEVYEIQVGRTKNMDIIYSVNVS------RGLHAVIWS
drgp130             PPNVEVKVEEDFPTSFVVKWEYDIEDTTMR---FSSAIRYCQVGSSDWKKVP-ENYTNVH
dril-12rb2.a        LPKIKHVDC----------SSRWCHLHTDNHSMNLVEVQYKSPKG------IWKKVQING
drgcsfr             PPSITRIEAHKYGCLKYSWSLSETQKWLQLTFIVQLRLKTVSNQPNKD---LVYTSRQLQ
drobr_CHD2          PPFDLEAIT-------LPSKTLSVRWKRPSLPVYGMQYELQFKALAGMANTQWKVIGPLL
drosmr_CHD2         PQRLEATAG------VFDSLVILHLNGSFSGLQLICQIELEPGGTVQES--VQDGSDSVQ
dril-23r            TPDIMQILLE-------NGSRFPTIYWNGSDNHFKPSLRYRRAHG-NHAWVLGNIAVVRI
dril-12rb2.b        PPYNMTHMF------------TPLEIFWDMDCDIMGTLDECHVQYHMQNHMLDWTEVDDC
drobr_CHD1          PSRELKPSP--------LSMKSPVFKHFGFPVSYVDNVRVVDKKKTQFCKLCYVLKVEGR
```

```
hsIL-12Rb2          DLLDLK----PFTEYEFQISSK-LHLYKGSWSDWS
mmIl-12rb2          DLRDLR----PFTEYEFQISSK-LHLSGGSWSNWS
hsLIFR_CHD1         SDMPLE-CAIHFVEIRCYIDNL-HFSGLEEWSDWS
mmLifr_CHD1         SDLPLQ-CATHSVSIRWHIDSP-HFSGYKEWSDWS
hsOSMR_CHD0.5       SELPLE-CATHFVRIKSLVDDA-KFPEPNFWSNWS
mmOsmr_CHD0.5       SDIPLE-CVKHFIRIRALVDDT-KSLPQSSWGNWS
hsIL-12b1           CLCPLEMNVAQEFQLRRRQ----LGSQGSSWSKWS
mmIl-12b1           CLCPSE-NMAQEIQIRRRRRLS-SGAPGGPWSDWS
hsOBR_CHD1          SLLVDSILP--GSSYEVQVRGK-RLDGPGIWSDWS
mmObr_CHD1          SLLVDSVLP--GSSYEVQVRSK-RLDGSGVWSDWS
hsOBR_CHD2          SKSVSLPVPDLCAVYAVQVRCK-RLDGLGYWSNWS
mmObr               SKSASLLVSDLCAVYVVQVRCR-RLDGLGYWSNWS
hsGCSFR             ALQYELCGLLPATAYTLQIRCI-RWPLPGHWSDWS
mmGcsfr             KDQFELCGLHQAPVYTLQMRCI-RSSLPGFWSPWS
hsIL-23R            YVQQSEFYLEPNIKYVFQVRCQ-ETGKRY-WQPWS
mmIl-23r            YVQQSEFYLEPDSKYVFQVRCQ-ETGKRN-WQPWS
hsOSMR_CHD2         EYFLS--ELEPATEYMARVRCA-DASHFWKWSEWS
mmOsmr_CHD2         NYLFS--DLDPDTKYKAFVRCA-SANHFWKWSDWT
hsLIFR_CHD2         SYLVALDKLNPYTLYTFRIRCS--TETFWKWSKWS
mmLifr_CHD2         TYHVAVDKLNPYTAYTFRVRCS--SKTFWKWSRWS
drlifr.b_CHD2       LASVVLEHLQPFTKYTAKVSCG-SHEHFFQWGDWS
drlifr.a_CHD2       LSSVVLEDLWPDVDYTVTVRCA-SKQSFWKWGDES
hsGP130             RSSFTVQDLKPFTEYVFRIRCM-KEDGKGYWSDWS
mmGp130             RTSFTVQDLKPFTEYVFRIRSI-KDSGKGYWSDWS
hsIL-27R            LTPVEIQDLELATGYKVYGRCR-MEKEEDLWGEWS
mmIl-27r            LTPVEMQNLEPGTCYQVSGRCQ-VENG-YPWGEWS
hsGLMR              NQTYNLTGLQPFTEYVIALRCA-VKES-KFWSDWS
mmGlmr              -QVCNLTGLQAFTEYVLALRFR-FNDS-RYWSKWS
drlifr.a_CHD0.5     SPLPLQ-----CTSHSVQIRAR-NQQSISPWS---
drlifr.b_CHD0.5     SPIPLQ-----CTSHSVRLRRR-DQLRISEWT---
drosmr_CHD0.5       SQLPLN-----CVNHAVRIRLITAASTFSSWSPWK
drgp130             ITSYRLQSLEPYTEYVVQMRSI--GEHYTIWSEWS
dril-12rb2.a        KKSLNISLLEPYTKYMFQIRRK-INQTMGLWSYWS
drgcsfr             LNPIEVCSLLHWTDYRSTVRVK--YYATSEWSEWS
drobr_CHD2          EPQAEIQLEESCVQFKVEVRCK-DVNDTGYWSDWS
drosmr_CHD2         LYLFRLQNLKPSTQYASRGRCA---VQGKHWSHWT
dril-23r            GCVFLQEPLEPLISYQFELRVCVSSANCSMWS---
dril-12rb2.b        QSMFRIEDPQPFTQYSFRVRCH-CGHEEKVMSNWS
drobr_CHD1          SWVALN-ELSSDIRYTVQVRCQ---NHLGYWSEWS
```
