# Supplementary material for: Evolution of Class I cytokine receptors
Source: BMC Evol Biol. 2007 Jul 18;7:120. doi: 10.1186/1471-2148-7-120 (PMC1963337; doi:10.1186/1471-2148-7-120)
Supplement: Additional file 6 — Alignment of the CHD of group 3 receptors. Additional file is a pdf document that contains supplementary data. Included is an alignment of group 3 Class I cytokine receptors that were used to calculate the phylogenetic tree in Figure 3c. [file 1471-2148-7-120-S6.pdf]

CLUSTAL X (1.83) multiple sequence alignment

```
drclf-1.b         CWSRN-TKDLSCRWSPGSQGETFINTKYVLKYKLKWYGKEKD-----------CEDY-TG
drclf-1.a         CWSRN-TKDLTCRWAPGGQGETFIKTKYTLKYKLRWYGREKE-----------CEDYSTG
hsCLF-1           CWSKN-MKDLTCRWTPGAHGETFLHTNYSLKYKLRWYGQDNT-----------CEEYHTV
mmClf-1           CWSRN-MKDLTCRWTPGAHGETFLHTNYSLKYKLRWYGQDNT-----------CEEYHTV
hsIL-6R           CFRKSPLSNVVCEWGPRSTPSLTTKAVLLVRKFQNSP-AEDFQ--------EPCQYSQES
mmIl-6r           CFRKNPLVNAICEWRPSSTPSPTTKAVLFAKKINTTNGKSDFQ--------VPCQYSQQL
hsIL-11R          CQAADYEN-FSCTWSPSQISG--LPTRYLTSYRKKTVLGADSQRRSPSTGPWPCPQDPLG
mmIl-11r          CQAVDYEN-FSCTWSPGQVSG--LPTRYLTSYRKKTLPGAESQRESPSTGPWPCPQDPLE
hsIL-27Rb         CRASRYPIAVDCSWTLPPAPNSTSPVSFIATYRLGMAARGHS---------WPCLQQTPT
mmIl-27rb         CHASRYPVAVDCSWTPLQAPNSTRSTSFIATYRLGVATQQQS---------QPCLQRSPQ
hsCNTFR           CRSNTYPKGFYCSWHLP------TPTYIPNTFNVTVLHGSKI---------MVCEKDPAL
mmCntfr           CRSNTYPKGFYCSWHLP------TPTYIPNTFNVTVLHGSKI---------MVCEKDPAL
drcntfr           CRSNTYPRGFYCSWHLQ------HPTFIPTDFEVDVQHNQRP---------LEVTRDTVH
hsIL-12p40        CEAKNYSGRFTCWWLTTISTDLTFSVKSSRGSSDPQGVTCGAATLSAERVRGDNKEYE-Y
mmIl-12p40        CEAPNYSGRFTCSWLVQRNMDLKFNIKSSSSSPDSRAVTCGTASLSAEKVTLDQRDYEKY
dril-27rb         CWSPSYPKRAICSWTLTSDP--ILPTQYIATYRNFSDPVSSAR---------QCQKWGDQ
dril-6ra          CNMKSHTSKVRCEWTSKQPVIPRPVCYLLLNRRFLKNTIHVN------------CSFSRS
dril-11R          CRVPN-HMKISCSWEQTKRTN--LPTSYRASYSQKGQGRKQIPS-------EPCEQKDTG
dril-12p40.a      CSARNYNGQFHCSWKWHHERT-HKAVVYFAAIRNSSDLNCTL-------------DSNIS
dril-12p40.b      CATNNYGGLFQCSWSWGENRHGHITHIEAIRPDGGSNISCSL-------------DPEGK
                  *          * *

drclf-1.b         QSYTCYVPRDLAIFTPYEAWVEATNQLGSATSDVIT-LDILDVVTTDPPPDVLVSRVGDL
drclf-1.a         EPYTCYIPRDLALFTPYEIWVEASNQLGTATSDVIY-LDILDVVTTDPPTDVTVSRVGDL
hsCLF-1           GPHSCHIPKDLALFTPYEIWVEATNRLGSARSDVLT-LDILDVVTTDPPPDVHVSRVGGL
mmClf-1           GPHSCHIPKDLALFTPYEIWVEATNRLGSARSDVLT-LDVLDVVTTDPPPDVHVSRVGGL
hsIL-6R           QKFSCQLAVPEGDSSFYIVSMCVASSVGSKFSKTQT-FQGCGILQPDPPANITVTAVARN
mmIl-6r           KSFSCQVEILEGDKVYHIVSLCVANSVGSKSSHNEA-FHSLKMVQPDPPANLVVSAIPGR
hsIL-11R          AARCVVHGAEFWS--QYRINVTEVNPLGAS-TRLLD-VSLQSILRPDPPQGLRVESVPGY
mmIl-11r          ASRCVVHGAEFWS--EYRINVTEVNPLGAS-TCLLD-VRLQSILRPDPPQGLRVESVPGY
hsIL-27Rb         STSCTITDVQLFSMAPYVLNVTAVHPWGSS-SSFVP-FITEHIIKPDPPEGVRLSPLAER
mmIl-27rb         ASRCTIPDVHLFSTVPYMLNVTAVHPGGAS-SSLLA-FVAERIIKPDPPEGVRLR-TAGQ
hsCNTFR           KNRCHIRYMHLFSTIKYKVSISVSNALGHN-ATAIT-FDEFTIVKPDPPENVVARPVPSN
mmCntfr           KNRCHIRYMHLFSTIKYKVSISVSNALGHN-TTAIT-FDEFTIVKPDPPENVVARPVPSN
drcntfr           KNRCHVKFPELFSSSPYYVNVTAVNSLGRA-STTIS-FEESSIVKPDPPEKVVAKPVANN
hsIL-12p40        SVECQEDSACPAAEESLPIEVMVDAVHKLKYENYTSSFFIRDIIKPDPPKNLQLKPLKNS
mmIl-12p40        SVSCQEDVTCPTAEETLPIELALEARQQNKYENYSTSFFIRDIIKPDPPKNLQMKPLKNS
dril-27rb         DRQCALEELEIFAREPTLINITAVNALGSA--TQIQPFIFEQIVKPDPPENVSGIIMPGR
dril-6ra          RCWCAFFVKEEYLRDLHRAQICVSNIAGSAMSPVLN-FNPHDTLRPDPPSRVLVSAVEGL
dril-11R          VNECTITDPPFWSSSKILVNITEINPLGSN--STIIQIDLRELLKPDPPEDVQVSQVEGE
dril-12p40.a      ELTCIDKDYCPYSEEPLSINLTLLVRNMFRLEEHHRTFFIRDIVKPD---KVGITKIQDN
dril-12p40.b      SITCLDQDYCPYAEELERINLTVYFRSSFVVESYNAKFYIMNIVKPD---MVAISRINHT
                                      :                .     : .*    :
```

```
drclf-1.b         EDQLSVRWGSPPALK--DYLFQAKYQIRYRVEESDEWKV---IDDAGNQ-TSCRLAGLRA
drclf-1.a         EDQLTVRWGTPPALK--DFLFQAKYQIRYRLEESSDWKV---VDDVGNQ-TSCRLAGLRP
hsCLF-1           EDQLSVRWVSPPALK--DFLFQAKYQIRYRVEDSVDWKV---VDDVSNQ-TSCRLAGLKP
mmClf-1           EDQLSVRWVSPPALK--DFLFQAKYQIRYRVEDSVDWKV---VDDVSNQ-TSCRLAGLKP
hsIL-6R           PRWLSVTWQDPHSWN--SSFYRLRFELRYRAERSKTFTT---WMVKDLQ-HHCVIHDAWS
mmIl-6r           PRWLKVSWQHPETWD--PSYYLLQFQLRYRPVWSKEFTV---LLLPVAQ-YQCVIHDALR
hsIL-11R          PRRLRASWTYPASWPC-QPHFLLKFRLQYRPAQHPAWST---VEPAG---LEEVITDAVA
mmIl-11r          PRRLHASWTYPASWRR-QPHFLLKFRLQYRPAQHPAWST---VEPIG---LEEVITDAVA
hsIL-27Rb         Q--LQVQWEPPGSWPF-PEIFSLKYWIRYKRQGAARFHR---VGPIEA--TSFILRAVRP
mmIl-27rb         R--LQVLWHPPASWPF-PDIFSLKYRLRYRRRGASHFRQ---VGPIEA--TTFTLRNSKP
hsCNTFR           PRRLEVTWQTPSTWPD-PESFPLKFFLRYRPLILDQWQH---VELSDG--TAHTITDAYA
mmCntfr           PRRLEVTWQTPSTWPD-PESFPLKFFLRYRPLILDQWQH---VELSDG--TAHTITDAYA
drcntfr           ARRLEVTWNSPTTWPD-VETFPLKYFLRYRPLIRDQWQH---VELSDS--TSHTITDAYA
hsIL-12p40        R-QVEVSWEYPDTWSTPHSYFSLTFCVQVQGK-SKREK-----------KDRVFTDKTS
mmIl-12p40        --QVEVSWEYPDSWSTPHSYFSLKFFVRIQRK-KEKMKE---TEEGCNQ-KGALLVEKTS
dril-27rb         K--VSVQWSPPHTWPD-PVNFLLKYKVRFHWGKPETART---LGPYES--NKMVLSGLVA
dril-6ra          KHVLKVSWSYPSSWKN--RFYVLQFHLRYRPQFAEQYQFGEISDKMERR-LSWMIYDALP
dril-11R          PTQLLVQWNCPPSWLVGMSAFSLTYLLRYRPIGSNYWSKVD-IESDTES-TSLIITDALI
dril-12p40.a      ----EFEWRPPQTWSFPCSFFPLSYEIKVVPRSHDCDYTGNRVEQNETN-KTHYKVNSKK
dril-12p40.b      ----CVEVGYPDSWNTPSSYFPLIFQVKEIRCRKRKKCDCSQLSSIETYRTQSLQLPVSK
                            * :       :   : ::

drclf-1.b         GTVYFVQVRCNPVGIYGSKKAGIWSDWS
drclf-1.a         GTVYFVQVRCNPVGILGSRKAGIWSDWS
hsCLF-1           GTVYFVQVRCNPFGIYGSKKAGIWSEWS
mmClf-1           GTVYFVQVRCNPFGIYGSKKAGIWSEWS
hsIL-6R           GLRHVVQLRAQ-----EEFGQGEWSEWS
mmIl-6r           GVKHVVQVRGK-----EELDLGQWSEWS
hsIL-11R          GLPHAVRVSARDFL-----DAGTWSTWS
mmIl-11r          GLPHAVRVSARDFL-----DAGTWSAWS
hsIL-27Rb         RARYYVQVAAQDLT-----DYGELSDWS
mmIl-27rb         HAKYCIQVSAQDLT-----DYGKPSDWS
hsCNTFR           GKEYIIQVAAKDN------EIGTWSDWS
mmCntfr           GKEYIIQVAAKDN------EIGTWSDWS
drcntfr           GKEYIIQLAAKDM------EIGTWSDWS
hsIL-12p40        ATVICRKNASISVRAQDRYYSSSWSEWA
mmIl-12p40        TEVQC-KGGNVCVQAQDRYYNSSCSKWA
dril-27rb         GRTYYIQISAKDIL-----DDGQSSEWS
dril-6ra          DTVYEVQLKAKDEF------DGSWSDWT
dril-11R          GHLHQIQIRAQDAL----INHSQWSEWS
dril-12p40.a      PYTFCIRAQDPLTK-------AVWSDWS
dril-12p40.b      GKMVCVRAKDEFCN-------SSWSEWS
                        :              .  * *:
```
