# Supplementary material for: Evolution of Class I cytokine receptors
Source: BMC Evol Biol. 2007 Jul 18;7:120. doi: 10.1186/1471-2148-7-120 (PMC1963337; doi:10.1186/1471-2148-7-120)
Supplement: Additional file 7 — Alignment of the CHD of group 4 receptors. Additional file is a pdf document that contains supplementary data. Included is an alignment of group 4 Class I cytokine receptors that were used to calculate the phylogenetic tree in Figure 3d. [file 1471-2148-7-120-S7.pdf]

CLUSTAL X (1.83) multiple sequence alignment

```
hsIL-21R               CYTDYLQTVICILEMWNLHPST---LTLTWQDQYEELKDEATSCSLHRSAHNATHATYTC
mmIl-21r               CYTDYLWTITCVLETRSPNPSI---LSLTWQDEYEELQDQETFCSLHRSGHNTTHIWYTC
hsIL-4Ra               CVSDYMSISTCEWKMNGPTNCS---TELRLLYQLVFL-LSEAHTCIPE---NNGGAGCVC
mmIl-4ra               CFSDYIRTSTCEWFLDSAVDCS---SQLCLHYRLMFFEFSENLTCIPR---NSASTVCVC
hsIL-2Rb               CFYNSRANISCVWSQ-DGALQD---TSCQVHAWPDRRRWNQTCELLPVSQASWACNLILG
mmIl-2rb               CFYNSRANVSCMWSH-EEALNV---TTCHVHAKSNLRHWNKTCELTLVRQASWACNLILG
hsIL-9R                CLTNNILRIDCHWSAPELGQGS---SPWLLFTSNQAPGGTHKCILRGS-----ECTVVLP
mmIl-9r                CLSNSIYRIDCHWSAPELGQES---RAWLLFTSNQVTEIKHKCTFWDS-----MCTLVLP
mmIl-3rbc_il3_CHD2     CFFDGIQSLHCSWEVWTQTTGS---VSFGLFYRP-----SPAAPEEKCSPVVKEPQ-ASV
mmIl-3rbc_CHD2         CFFDGIQSLHCSWEVWTQTTGS---VSFGLFYRP-----SPVAPEEKCSPVVKEPPGASV
hsIL-3Rbc_CHD2         CFFDGAAVLSCSWEVRKEVASS---VSFGLFYKP-----SPDAGEEECSPVLREGL-GSL
mmIl-3rbc_il3_CHD1     CYNDYTNRIICSWADTEDAQGL---INMTLLYHQLDKI-QSVSCELSEKLMWSECPSSHR
mmIl-3rbc_CHD1         CYNDYTNHIICSWADTEDAQGL---INMT-LYHQLEKK-QPVSCELSEKLMWSECPSSHR
hsIL-3Rbc_CHD1         CYNDYTSHITCRWADTQDAQRL---VNVTLIRRVNEDLLEPVSCDLSDDMPWSACP-HPR
hsIL-7R                -----------CYSQLEVNGSQ---HSLTCAFEDPDVNITNLEFEICGALVEVKCLNFRK
mmIl-7r                -----------CHSQLEVDGSQ---HLLTCAFNDSDINTANLEFQICGALLRVKCLTLNK
dril-2rb               CVNDYMTNISCVWWNYSNFSNQQCELEVNCKISPSSQSKSSRCELVPQNNLSNSPRSCFL
dril-21r.a             CTTDFISALNCSASDLTGTASCDVVASCRDEFSTVNGSCSIRSP-QSWCTMEPYELESIM
dril-3rbc_CHD2         CVIEGETTVMCTWQMRTEYFQF-------MTYHMLCHDNTGHHPCCKTPQLKSSNDELIE
dril-3rbc_CHD1         CYNDYKSYTECTWETDPRANFTLYYYDKNKNLETLCLLNKPHVVLPNGKLSHVCRYNTVR
dril-7ra               ----------CTSTLTLMQNIL-------YCFPNDEPIEEVIFATLCKMKEKSKCTNATL
dril-4ra               CFNDYEAEMTCSFSSESLRNCS------GYNMNVTQKLAYEINRYSCVFERSHHNAICEC
dril-21r.b             SDHISRTARSCRFAFYNIRKMLQNAVARVVFNEPKRANVTPLLIRLHWLPVAARIKFKAL


hsIL-21R               HMDVFHF--------MADDIFS---VNITDQSGNYSQECGSFLLAE----SIKPAPP--F
mmIl-21r               HMRLSQF--------LSDEVFI---VNVTDQSGNNSQECGSFVLAE----SIKPAPP--L
hsIL-4Ra               HLLMDDV--------VSADNYT---LDLWAGQQLLWK--GSFKPSE----HVKPRAPGNL
mmIl-4ra               HMEMNRP--------VQSDRYQ---MELWAEHRQLWQ--GSFSPSG----NVKPLAPDNL
hsIL-2Rb               -APDSQK--------LTTVDIVTLRVLCREGVRWRVMAIQDFKPFE----NLRLMAPISL
mmIl-2rb               SFPESQS--------LTSVDLLDINVVCWEEKGWRRVKTCDFHPFD----NLRLVAPHSL
hsIL-9R                --PEAVL--------VPSDNFTITFHHCMSGREQVSLVDPEYLPRR----HVKLDPPSDL
mmIl-9r                --KEEVF--------LPFDNFTITLHRCIMGQEQVSLVDSQYLPRR----HIKLDPPSDL
mmIl-3rbc_il3_CHD2     YTRYRCS--------LPVPEPS-AHSQYTVSVKHLEQGKFIMSYYH----IQMEPP---I
mmIl-3rbc_CHD2         YTRYHCS--------LPVPEPS-AHSQYTVSVKHLEQGKFIMSYNH----IQMEPP---T
hsIL-3Rbc_CHD2         HTRHHCQ--------IPVPDPA-THGQYIVSVQPRRAEKHIKSSVN----IQMAPP---S
mmIl-3rbc_il3_CHD1     CVPRRCV--------IPYTRFSNGDNDYYSFQPDRDLGIQLMVPLA----QHVQPPPPKD
mmIl-3rbc_CHD1         CVPRRCV--------IPYTRFSITNEDYYSFRPDSDLGIQLMVPLA----QNVQPPLPKN
hsIL-3Rbc_CHD1         CVPRRCV--------IPCQSFVVTDVDYFSFQPDRPLGTRLTVTLT----QHVQPPEPRD
hsIL-7R                LQEIYFI--------ETKKFLLIGKSNICVKVGEKSLTCKKIDLTT----IVKPEAPFDL
mmIl-7r                LQDIYFI--------KTSEFLLIGSSNICVKLGQKNLTCKNMAINT----IVKAEAPSDL
dril-2rb               FCENAYF--------FFLSKMV-----LSVSCNGSLISSLHYKPGR----HIKTQPP--D
dril-21r.a             SFDTNCF--------ITVTQMD----KEGDMEIPTQRYSKNIVLYK----STKPKQPFNL
dril-3rbc_CHD2         FMCSVNV--------IDPNQLT--------VELKQVLFTRTFKTAH----HIKLPQPDKL
dril-3rbc_CHD1         FGLNTNH--------TLYFKVQCGNTPTSLRVAQHVEKSFTVMCMI----SGKVRAPVNL
dril-7ra               GPKYFSF--------ENLENIVKYELQVHLRDG---IIEKEIDLTK----MVKIPAPELK
dril-4ra               KIEVEGF--------ITEEIFS-----TTLLKGTKVLLQRDFKTID----YIKPKTPVLF
dril-21r.b             MFAYKATSGFAPSYLLSLLQISLVTHDPLVLCDWLFAACVTLSIAGGTHLITNVKPPAPF
```

```
hsIL-21R                NVTVTFSG-QYNISWRSDYED-PAFYMLKGKLQYELQYRNRGDPWAVSPRRKLISVDSRS
mmIl-21r                NVTVAFSG-RYDISWDSAYDE-PSNYVLRGKLQYELQYRNLRDPYAVRPVTKLISVDSRN
hsIL-4Ra                TVHTNVSD-TLLLTWSNPY---PPDNYLYNHLTYAVNIWSENDPADFRIYN--VTYLEPS
mmIl-4ra                TLHTNVSD-EWLLTWNNLY---PSNNLLYKDLISMVNISREDNPAEFIVYN--VTYKEPR
hsIL-2Rb                QVVHVETH-RCNISWEIS----QASHYFERHLEFEARTLSPGHTWEEAPLLT---LKQKQ
mmIl-2rb                QVLHIDTQ-RCNISWKVS----QVSHYIEPYLEFEARRRLLGHSWEDASVLS---LKQRQ
hsIL-9R                 QS-NISSG-HCILTWSIS----PALEPMTTLLSYELAFKKQEEAWEQAQHRD---HIVGV
mmIl-9r                 QS-NVSSG-RCVLTWGIN----LALEPLITSLSYELAFKRQEEAWE-ARHKD---RIVGV
mmIl-3rbc_il3_CHD2      LNQTKNRD-SYSLHWET-----QKIP-KYIDHTFQVQYKKKSESWKDSKTEN---LGRVN
mmIl-3rbc_CHD2          LNLTKNRD-SYSLHWET-----QKMAYSFIEHTFQVQYKKKSDSWEDSKTEN---LDRAH
hsIL-3Rbc_CHD2          LNVTKDGD-SYSLRWET-----MKMRYEHIDHTFEIQYRKDTATWKDSKTET---LQNAH
mmIl-3rbc_il3_CHD1      IHISPSGD-HFLLEWSVSLGDSQVSWLSSKDIEFEVAYKRLQDSWEDASSLH---TSNFQ
mmIl-3rbc_CHD1          VSISSSED-RFLLEWSVSLGDAQVSWLSSKDIEFEVAYKRLQDSWEDAYSLH---TSKFQ
hsIL-3Rbc_CHD1          LQISTDQD-HFLLTWSVALGSPQSHWLSPGDLEFEVVYKRLQDSWEDAAILL---SNTSQ
hsIL-7R                 SVVYREGANDFVVTFNTS----HLQKKYVKVLMHDVAYRQEKDENKWTHVN----LSSTK
mmIl-7r                 KVVYRKEANDFLVTFNAP----HLKKKYLKKVKHDVAYRPARGESNWTHVS----LFHTR
dril-2rb                KPVVNGSN----VSWSKG----SNFPKSIKKHEFQLQFKDAHTSWEMAKPGQ--LSQENY
dril-21r.a              TIRNIDGG--FNLTWDVAY----TDNVLYEKLIYRVRVLSKSSSPKENIYTLQ--QNQQS
dril-3rbc_CHD2          NTMEVDDA--LQLNWSIT------AVKRGIEFTAEIKLSTNKESMIFNCTER-----VNT
dril-3rbc_CHD1          TEMMTDGG-GRLLSWKSPY---PASSNITRSLMYQLQYRRHMHDWTTVDKIN-----VSE
dril-7ra                SATFLKETDEIFIWFEHR-------HDYVRQCQFQVEIRGEHEPISLLVDYK-------N
dril-4ra                VKKTENGN--FHVTWVDSY---EQRNVFTDNLFITLTYRIKGEIETNSTKVAN---TVGF
dril-21r.b              NLTLLENG-TYKFFWKSGY--EAYRYWRVLPLRYQFLYYKDGEHHNVSAKHH---VYKEE
                                    . :

hsIL-21R                VSLLPLEFRKDSSYELQVRAGPMPGSSYQ-----------GTWSEWS
mmIl-21r                VSLLPEEFHKDSSYQLQVRAAPQPGTSFR-----------GTWSEWS
hsIL-4Ra                LRIAASTLKSGISYRARVRAWAQ---CYN-----------TTWSEWS
mmIl-4ra                LSFPINILMSGVYYTARVRVRSQ---ILT-----------GTWSEWS
hsIL-2Rb                EWICLETLTPDTQYEFQVRVKPLQG-------------EFTTWSPWS
mmIl-2rb                QWLFLEMLIPSTSYEVQVRVKAQRN-------------NTGTWSPWS
hsIL-9R                 TWLILEAFELDPGFIHEARLRVQMATLE--DDVVEEERYTGQWSEWS
mmIl-9r                 TWLILEAVELNPGSIYEARLRVQMT-LESYEDKTEGEYYKSHWSEWS
mmIl-3rbc_il3_CHD2      S-MDLPQLEPDTSYCARVRVKP--ISDYD-----------GIWSEWS
mmIl-3rbc_CHD2          S-MDLSQLEPDTSYCARVRVKP--ISNYD-----------GIWSKWS
hsIL-3Rbc_CHD2          S-MALPALEPSTRYWARVRVRTS-RTGYN-----------GIWSEWS
mmIl-3rbc_il3_CHD1      VNLEPKLFLPNSIYAARVRTRLSAGSSLS-----------GRPSRWS
mmIl-3rbc_CHD1          VNFEPKLFLPNSIYAPRVRTRLYPGSSLS-----------GRPSRWS
hsIL-3Rbc_CHD1          ATLGPEHLMPSSTYVARVRTRLAPGSRLS-----------GRPSKWS
hsIL-7R                 LTLLQRKLQPAAMYEIKVRSIP-DHYFKG------------FWSEWS
mmIl-7r                 TTIPQRKLRPKAMYEIKVRSIPHNDYFKG------------FWSEWS
dril-2rb                TQLNHDLLTVGEEYQARVRVKPVEPKTDG--------HFRGEWSEWS
dril-21r.a              MVILSEKLQPGLQHVADVQVAVNPEWFPS------------MWSEWS
dril-3rbc_CHD2          YRIPFKFLHPSTDYLAQIRFVPIPKKDYR-----------LQPSDWS
dril-3rbc_CHD1          HTIIKESLFPGYHYEARVRARGPVG----------------LWSNWS
dril-7ra                ISMSRDRLGGDGVYSTRVRAKPINYFAGD-------------WSEWS
dril-4ra                CDIVGSLLQPKTEYILTAKMSSNYNGQK-------------IYSDQS
dril-21r.b              IEIENKKLDPGTSYSAVVRTGIETHPKYS-----------GTWSDWS
                          :    .          :                        *  *
```
