# Supplementary material for: Evolution of Class I cytokine receptors
Source: BMC Evol Biol. 2007 Jul 18;7:120. doi: 10.1186/1471-2148-7-120 (PMC1963337; doi:10.1186/1471-2148-7-120)
Supplement: Additional file 8 — Alignment of the CHD of group 5 receptors. Additional file is a pdf document that contains supplementary data. Included is an alignment of group 5 Class I cytokine receptors that were used to calculate the phylogenetic tree in Figure 4. [file 1471-2148-7-120-S8.pdf]

CLUSTAL X (1.83) multiple sequence alignment

```
hsIL-13Ra1      -----CIWHNLS-----------YMKCSWLPGRNTSPDTNYTLYYWHRSL--EKIHQCEN
mmIl-13ra1      -----CIWHNLS-----------YMKCSWLPGRNTSPDTHYTLYYWYSSL--EKSRQCEN
hsIL-5ra        -----CTTNTTEDNYSRLRSYQVSLHCTWLVGTDAPEDTQYFLYYRYGSW--TEECQEYS
mmIl-5ra        -----CTTHTVVSSHTHLRPYQVSLRCTWLVGKDAPEDTQYFLYYRFGVL--TEKCQEYS
hsIL-13Ra2      -----CVYYNWQ-----------YLLCSWKPGIGVLLDTNYNLFYWYEGL--DHALQCVD
mmIl-13ra2      -----CIYYNWQ-----------YLVCSWKPGKTVYSDTNYTMFFWYEGL--DHALQCAD
hsIL-2Rgc       -----CFVFNVE-----------YMNCTWN-SSSEPQPTNLTLHYWYKNS--DNDKVQKC
mmIl-2rgc       -----CFVFNIE-----------YMNCTWN-SSSEPQATNLTLHYRYKVS--DNNTFQEC
dril-13ra2      -----CIYYGKE-----------HMECTWKSGPVQPPNSEHYLYYWHREM--EETKECPE
hsGMRa          -----CFIYNAD-----------LMNCTWARGPTAPRDVQYFLYIRNSKR--RREIRCPY
mmGmra          -----CEIRAAR-----------FLSCAWREGPAAPADVRYSLRVLNSTG--HDVARCMA
hsIL-3Ra        -----CWIHDVD-----------FLSCSWAVGPGAPADVQYDLYLNVANR--RQQYECLH
mmIl-3ra        -----CWVHEGQ------------LSCQWERGPKATGDVHYRMFWRDVRLGPAHNRECPH
dril-2rgc.a     -----CLIINLD-----------YVNCTWS-----EHQYNYSLKGGFSYF---DIEDCPE
dril-2rgc.b     -----CKIINVD-----------YVECIWQRN---LSTMNYTFSSMFANDG--LDRGCPK
huTSLPR         ---IQIIYFNLET-----------VQVTWN--ASKYSRTNLTFHYRFNGD--EAYDQCTN
mmTslpr         ---VTVVCHDLET-----------VEVTWGSGPD-HHSANLSLEFRYGTG---ALQPCPR
drtslpr         ------------------------------------------------------------
dril-13ra1      LVKDRDFAYSHN-----------KLMCTWR---PAVDVKDLGFYYWDSSL--ESLMRCIP


hsIL-13Ra1      I---------FRE-GQYFGCSFDLTKVKDSSFEQH------SVQIMVKDNAGKIKPSFNI
mmIl-13ra1      I---------YRE-GQHIACSFKLTKVEP-SFEHQ------NVQIMVKDNAGKIRPSCKI
hsIL-5ra        K---------DTL-GRNIACWFPRTFILSKGRDWL------SVLVNGSSKHSAIRPFDQL
mmIl-5ra        R---------DAL-NRNTACWFPRTFINSKGFEQL------AVHINGSSKRAAIKPFDQL
hsIL-13Ra2      Y---------IKADGQNIGCRFP--YLEASDYKDF------YICVNGSSENKPIRSSYFT
mmIl-13ra2      Y---------LQHDEKNVGCKLS--NLDSSDYKDF------FICVNGSSKLEPIRSSYTV
hsIL-2Rgc       S---------HYLFSEEITSGCQLQKKEIHLYQTF------VVQLQ--DPREPRRQATQM
mmIl-2rgc       S---------HYLFSKEITSGCQIQKEDIQLYQTF------VVQLQ--DPQKPQRRAVQK
dril-13ra2      Y---------IVSSNNERGCRFP--RQSLLEFSKF------NMCVNGSSSTGSLRPAYFS
hsGMRa          Y---------IQDSGTHVGCHLDN---LSGLTSRN------YFLVNGTSREIGIQFFDSL
mmGmra          D---------PGD-DVITQCIAN---DLSLLGSEA------YLVVTGRSGAGPVRFLDDV
hsIL-3Ra        YK--------TDAQGTRIGCRFDDISRLSSGSQSS------HILVRGRSAAFGIPCTDKF
mmIl-3ra        YHSLDVNTAGPAPHGGHEGCTLDLDTVLGSTPNSPDLVPQVTITVNGSGRAGPVPCMDNT
dril-2rgc.a     Y---------ETANGVNVACMLP----YKERTQRFN------TLKTSLYRDDGSLVTEQE
dril-2rgc.b     Y----------ILEQNYTGCRIP----LKDQNLRFN------KFIVTIYTEGKHTISKTF
huTSLPR         Y---------LLQEGHTSGCLLDAEQRDDILYFSIR--------------NGTHPVFTAS
mmTslpr         Y--------FLSGAGVTSGCILPAARAGLLELALR--------------DGGGAMVFKAR
drtslpr         ---------------------------PDRIVFNIS------------------KTPYGVK
dril-13ra1      D-----------DTMKMGGCVIHNKRLKEINVFSQ-----MFYLFNGTYNGTVVNNTFRD
```

```
hsIL-13Ra1       VPLTSRVKPDPPHIKNLSFH-NDDLYVQWENPQ-NFISRCLFYEVEVNNSQTETH---NV
mmIl-13ra1       VSLTSYVKPDPPHIKHLLLK-NGALLVQWKNPQ-NFRSRCLTYEVEVNNTQTDRH---NI
hsIL-5ra         FALHAIDQINPPLNVTAEIE-GTRLSIQWEKPVSAFPIHCFDYEVKIHNTRNG-------
mmIl-5ra         FSPLAIDQVNPPRNVTVEIE-SNSLYIQWEKPLSAFPDHCFNYELKIYNTKNG-------
hsIL-13Ra2       FQLQNIVKPLPPVYLTFTRESSCEIKLKWSIPLGPIPARCFDYEIEIREDDTT-------
mmIl-13ra2       FQLQNIVKPLPPEFLHISVENSIDIRMKWSTPGGPIPPRCYTYEIVIREDDIS-------
hsIL-2Rgc        LKLQNLVIPWAPENLTLHKLSESQLELNWNNRF--LNH-CLEHLVQYRTDWDHS------
mmIl-2rgc        LNLQNLVIPRAPENLTLSNLSESQLELRWKSRH--IKERCLQYLVQYRSNRDRS------
dril-13ra2       IEIQNYVKPAAVSSLDVLET-DGWLKLEWAPPSGQVPEHCLDYEVESSTLMANGKE-LKQ
hsGMRa           LDTKKIERFNPPSNVTVRCN-TTHCLVRWKQPRTYQKLSYLDFQYQLDVHRKNTQPGTEN
mmGmra           VATKALERLGPPRDVTASCN-SSHCTVSWAPPSTWASLTARDFQFEVQWQSAEPGS----
hsIL-3Ra         VVFSQIEILTPP-NMTAKCN-KTHSFMHWKMRS--HFNRKFRYELQIQKRMQP-------
mmIl-3ra         VDLQRAEVLAPP-TLTVECN-GSEAHARWVARN-RFHHGLLGYTLQVNQSSRS-------
dril-2rgc.a      HMLKEYVKLNPPTNLSVVEKKDAELWLYWNVTK---NDNCIESEVRYRTDQNN-------
dril-2rgc.b      DSLERNVLLNPPYNLSVMWNEHSDTLSLYWNSSAPLRKTCIVYMVRNQKDPSQ-------
huTSLPR          RWMVYYLKPSSPKHVRFSWH-QDAVTVTCSDLS----YGDLLYEVQYRSPFDT-------
mmTslpr          QRASAWLKPRPPWNVTLLWTPDGDVTVSWPAHS----YLGLDYEVQHRESNDDED-----
drtslpr          VIWRVANNNADPQCFNTEVQYKRQCEKDWERSH----------RIKLQNNQEE-------
dril-13ra1       ESAMQYVRLKKP-QLTVQRVGDSLMFQTNASDLDEFEESCYKYNYTYNECGKE-------


hsIL-13Ra1       FYVQEAKCENPEFERNVENTSCFMVPGVLPDTLNTVRIRVKTNKLCYEDDKLWSNWS
mmIl-13ra1       LEVEEDKCQNSESDRNMEGTSCFQLPGVLADAVYTVRVRVKTNKLCFDDNKLWSDWS
hsIL-5ra         -YLQIEKLMTNAFISIIDDLS-----------KYDVQVRAAVSSMCREAG-LWSEWS
mmIl-5ra         -HIQKEKLIANKFISKIDDVS-----------TYSIQVRAAVSSPCRMPG-RWGEWS
hsIL-13Ra2       --LVTATVENETYTLKTTNET----------RQLCFVVRSKVNIYCSDDG-IWSEWS
mmIl-13ra2       --WESATDKNDMKLKRRANES----------EDLCFFVRCKVNIYCADDG-IWSEWS
hsIL-2Rgc        -WTEQSVDYRHKFSLPSVDGQ----------KRYTFRVRSRFNPLCGSAQ-HWSEWS
mmIl-2rgc        -WTELIVNHEPRFSLPSVDEL----------KRYTFRVRSRYNPICGSSQ-QWSKWS
dril-13ra2       TEILENLDLGTSYELPREAES----------KKTCFNLRSKVNMYCADGG-FWSDWS
hsGMRa           LLINVSGDLENRYNFPSSEPR----------AKHSVKIRAADVRIL-----NWSSWS
mmGmra           TPRKVLVVEETRLAFPSPAPHG----------GHKVKVRAGDTRMK-----HWGEWS
hsIL-3Ra         ---VITEQVRDRTSFQLLNPG-----------TYTVQIRARERVYE-----FLSAWS
mmIl-3ra         ---EPQEYNVSIPHFWVPNAG-----------AISFRVKSRSEVYPR----KLSSWS
dril-2rgc.a      -WKDTTPGVRTTYSLPFPSNK-----------QYKFQVRARITSSCGQSK-FWSEWS
dril-2rgc.b      ----SLNVTDTSYSVSPVSQN----------KLYVFQVRSTVADSCGSSD-FWSDWS
huTSLPR          ---EWQSKQENTCNVTIEGLD--------AEKCYSFWVRVKAMEDVYGPDTYPSDWS
mmTslpr          ---AWQTTSGPCCDLTVGGLDP--------ARCYDFRVRASPRAAHYGLEAQPSEWT
drtslpr          ----------NHLNLSTLSTK----------MNYDFRIRMKY--ECLEKD--WSNWT
dril-13ra1       ---SRNFTEHISKSTKQFDQY----------CKYKARVQIHFSSNCGRG---FSELS
                                                      ::              .  :
```
